# A pangenome of tetraploid wheat reveals the genetic architecture underlying domestication and genomic diversity for breeding

**DOI:** 10.1038/s41588-026-02678-9

**Published:** 2026-07-22

**Authors:** Jianxin Bian, Guang Yang, Dong Xu, Yan Zhang, Yanzhe Jia, Guifen Zhang, Zhen Qin, Shuangxing Zhang, Yiqiao Wang, Mengmeng Jiang, Yan Pan, Bin Chen, Fangyu Liu, Yuqing Lu, Shuai Ding, Jiabo Wang, Can Yuan, Yongming Chen, Shoucheng Liu, Hang He, Shuai Wang, Qidi Zhu, Shisheng Chen, Qing Sang, Xing Wang Deng, Hude Mao, Xiaojun Nie, Baoxing Song

**Affiliations:** 1 State Key Laboratory of Wheat Improvement, Peking University Institute of Advanced Agricultural Sciences, Shandong Laboratory of Advanced Agricultural Sciences at Weifang, Weifang, China; 2 State Key Laboratory for Crop Stress Resistance and High-Efficiency Production, College of Agronomy, Northwest A&F University, Yangling, China; 3 Tea Research Institute, Chongqing Academy of Agricultural Sciences, Chongqing, China; 4 Oil Crop Research Institute, Chongqing Academy of Agricultural Sciences, Chongqing, China; 5 National Key Facility for Crop Gene Resources and Genetic Improvement, Institute of Crop Sciences, Chinese Academy of Agricultural Sciences, Beijing, China; 6 Key Laboratory of Qinghai-Tibetan Plateau Animal Genetic Resource Reservation and Utilization, Sichuan Province and Ministry of Education, Southwest Minzu University, Chengdu, China; 7 Peking–Tsinghua Center for Life Sciences, School of Life Sciences and School of Advanced Agricultural Sciences, Peking University, Beijing, China; 8 School of Agriculture, Henan Institute of Science and Technology, State Key Laboratory of High-Efficiency Production of Wheat-Maize Double Cropping, Xinxiang, China; 9 Innovative Center of Molecular Genetics and Evolution, School of Life Sciences, Guangzhou University, Guangzhou, China

**Keywords:** Plant breeding, Plant genetics

## Abstract

Tetraploid wheat (*Triticum turgidum* L., BBAA), a key pasta crop, serves as an untapped genetic resource with rich genomic diversity for hexaploid bread wheat improvement. Here we de novo assembled 12 genomes spanning all 10 recognized tetraploid wheat (genome BBAA) subspecies, and a graph-based pangenome was constructed. Chromosome rearrangements drove subgenome asymmetry and shaped genomic divergence, with an average of 0.25 million structural variations per accession, predominantly attributable to transposon activity. Using 736 globally distributed tetraploid wheat accessions, we identified locally adapted subgroups with untapped breeding potential and discovered a novel retrotransposon‑induced loss‑of‑function *Btr1-A* allele responsible for convergent adaptation of non-brittle rachis. Genome-wide association studies identified 287 loci associated with 32 traits. A homeodomain-leucine zipper transcription factor HAT14-B that enhances both spikelet number and grain size was identified. This subspecies-wide pangenome enriches Triticeae AB subgenome resources and facilitates the discovery and application of agronomically important genetic variations.

## Abstract (web summary)

A pangenome of tetraploid wheat constructed from 12 de novo genome assemblies spanning 10 subspecies, integrating with whole-genome sequencing data, highlights genetic variation associated with agricultural traits.

## Main

Tetraploid wheat (*Triticum turgidum* L., BBAA, 2*n* = 4*x* = 28) contributes two subgenomes (B and A) to the origin of hexaploid bread wheat (*Triticum aestivum* L., BBAADD, 2*n* = 6*x* = 42, also known as common wheat), and serves as a valuable genetic reservoir for its improvement^1,2^. Domesticated tetraploid wheat is a primary crop for non-bread-wheat-based food products, including pasta, bulgur and couscous, with an annual production of 37–40 million tons (refs. ^3,4^). Tetraploid wheat (*T. turgidum* L.) encompasses a spectrum of breeding-improved cultivars, domesticated landraces, as well as wild types^5^, which are absent from bread wheat. This diversity makes tetraploid wheat an ideal system for investigating the genetic basis of wheat domestication.

Tetraploid wheat (*T. turgidum* L.) is thought to have originated from the Near East^6^ about 800,000 years ago^7^ and consists of ten distinct subspecies distributed across diverse geographical regions. The evolutionary history of tetraploid wheat is approximately 80 times longer than that of bread wheat^7^, and has much greater genetic diversity^8^. Numerous key functional genes associated with breeding values have been identified in tetraploid wheat, and exploited for breeding and genetically validated in bread wheat^9–11^. Exploring untapped genomic resources in tetraploid wheat can broaden the genetic basis of bread wheat and advance future breeding amid rising global food demand. Though available genomes of wild emmer wheat (*Triticum dicoccoides*) Zavitan^12^, durum wheat cultivar Svevo^13^ and Langdon genome^14^ have facilitated key findings, a comprehensive graph-based pangenome and a high-resolution variation map remain essential for efficient identification of allelic variants and candidate genes for bread wheat improvement.

Here we de novo assembled 12 tetraploid wheat genomes, covering all 10 known subspecies^2^, to establish a comprehensive graph-based pangenome. This genome panel facilitated the investigation of genomic variations and evolutionary relationships, including genes, transposable elements (TEs) and structural variants (SVs). Using 736 tetraploid wheat accessions, we constructed a genetic-variation atlas and detected 287 loci underlying 32 agronomic traits via genome-wide association studies (GWAS). The graph-based pangenome offers a fundamental platform for identifying elite genes such as *HAT14* for wheat improvement.

This extensive genomic resource not only provides new insights into the evolutionary and genetic architecture of tetraploid wheat but also offers a powerful foundation for the genetic improvement of wheat and other Triticeae crops, with potentially broad implications for global food security and sustainable agriculture.

## Results

### High-quality genome assemblies and annotations

**Fig. 1:**
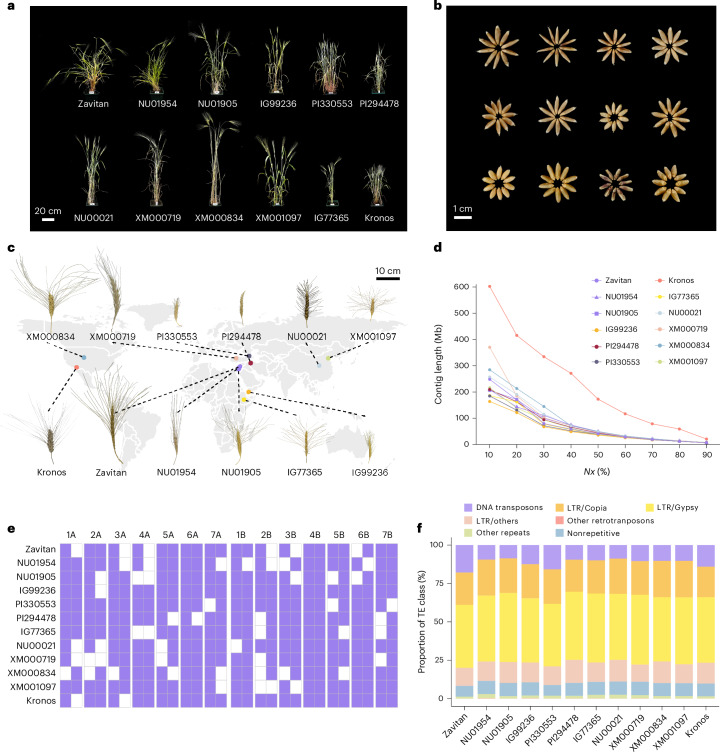
Phenotypes and genome characterization. **a**, Plant structures of the 12 sequenced tetraploid wheat accessions. **b**, Seed morphology of the 12 sequenced accessions. **c**, Geographical distribution and spike morphology. **d**, Evaluation of the continuities for the 12 genome assemblies. *Nx* (%), length of the shortest contig in the group of the longest contigs that represent *x%* of the assembly length. **e**, Presence and absence of telomeres in each genome assembly. Purple squares indicate the presence of telomeres and white squares represent their absence. **f**, Proportion of annotated TEs in each genome. LTR/unknown was included in LTR/others class. The map in **c** was plotted using the R package rworldmap (GPL v.2 license, https://github.com/AndySouth/rworldmap), with base map data from Natural Earth (http://www.naturalearthdata.com).

To construct a tetraploid wheat pangenome, we collected 12 accessions covering all 10 known tetraploid wheat (*T. turgidum* L.) subspecies (Table 1 and Supplementary Table 1). Phenotypes of the 12 accessions are diverse, including plant height, tiller angle, seed size and shape, awn length and spike morphology (Fig. 1a–c). For durum cultivar (cv.) Kronos, we assembled a highly contiguous genome with a contig N50 of 172.76 megabases (Mb) using 998.37 gigabases (Gb) of PacBio HiFi reads, 576.75 Gb of Oxford Nanopore Technology (ONT) reads (length ≥100 kilobases (kb)) and 1.04 terabases (Tb) of Hi-C reads (Supplementary Fig. 1 and Supplementary Note 1). On the basis of contig N50 saturation analysis (Supplementary Fig. 2), we produced an average of 556.46-Gb PacBio HiFi and 439.39-Gb Hi-C reads for de novo assembly of the other 11 tetraploid wheat accessions. Contig N50 scores for these 11 genomes were 35.79–49.81 Mb, averaging 43.2 Mb (Fig. 1d) and genome length ranged between 10.45 Gb and 10.65 Gb (Supplementary Table 1). All 12 accessions were anchored to the chromosome level using Hi-C reads, achieving an average anchoring rate of 98.81% (Table 1 and Supplementary Fig. 3). Meanwhile, an average of 23 telomeres for each assembly were identified, of which accession IG99236 (*Triticum dicoccon*) has the most detectable telomeres (*n* = 27), whereas accession XM000834 (*T. turgidum*) has the least (*n* = 20; Fig. 1e). These assemblies achieved a benchmarking universal single-copy orthologs (BUSCO) completeness score of 99%, a quality value (QV) of 69.41 and a long terminal repeat assembly index (LAI) of 19.19, demonstrating high quality and contiguity. Coupled with good synteny with published assemblies^12–14^, these metrics indicate the high quality and high contiguity of the genomic resources (Table 1, Supplementary Figs. 4–6 and Supplementary Note 2).

**Table 1:** Assembly and annotation statistics for the 12 tetraploid wheat genomes

Accession name	Type	Subspecies	Assembly size (Gb)	Contig N50 (Mb)	Repeat (%)	Genome BUSCO (%)	QV	LAI	No. of HC genes
Kronos	Free-threshing tetraploid wheat	*Triticum durum*	10.82	172.76	91.67	99.0	66.02	21.79	85,172
XM001097	Free-threshing tetraploid wheat	*Triticum turanicum*	10.49	38.24	91.68	98.9	71.19	18.72	85,640
NU00021	Free-threshing tetraploid wheat	*Triticum carthlicum*	10.50	48.26	91.42	99.0	71.07	18.67	84,714
IG77365	Free-threshing tetraploid wheat	*Triticum aethiopicum*	10.45	37.87	91.50	99.1	69.97	18.65	84,531
XM000719	Free-threshing tetraploid wheat	*Triticum polonicum*	10.65	46.46	91.28	99.0	69.61	18.81	89,423
XM000834	Free-threshing tetraploid wheat	*Triticum turgidum*	10.51	49.32	91.56	98.9	67.34	18.88	84,608
PI294478	Domesticated tetraploid wheat	*Triticum ispahanicum*	10.51	43.62	91.56	99.0	70.51	18.68	85,045
PI330553	Domesticated tetraploid wheat	*Triticum karamyschevii*	10.47	38.44	92.97	99.0	68.15	19.53	78,276
IG99236	Domesticated tetraploid wheat	*Triticum dicoccon*	10.46	35.79	91.41	99.0	70.42	19.11	84,451
NU01905	Wild emmer wheat	*Triticum dicoccoides*	10.61	46.26	91.44	99.0	70.19	18.67	84,777
NU01954	Wild emmer wheat	*Triticum dicoccoides*	10.56	49.81	91.48	99.1	69.22	18.81	83,408
Zavitan	Wild emmer wheat	*Triticum dicoccoides*	10.51	41.17	92.95	99.0	69.15	18.81	78,379

TEs account for 90.92–92.81% of these tetraploid wheat genomes, with long terminal repeat retrotransposons (LTR‑RTs) as the predominant class (Fig. 1f and Supplementary Table 2). Centromere regions across all 12 accessions were identified by CENH3 chromatin immunoprecipitation and sequencing (ChIP–seq)^15^ and confirmed by repeat-sequence analysis (Supplementary Table 3 and Supplementary Figs. 7 and 8), ranging from 6.38 Mb (chromosome 5B of IG99236) to 20.47 Mb (chromosome 6 A of Zavitan) in length. Using ab initio prediction, homology-search and transcriptome-based evidence, 78,276 to 89,423 high-confidence (HC) predicted genes (with an average functional annotation rate of 94.17%) were identified for the 12 new assemblies as well as the published Svevo genome^13^ (Table 1 and Supplementary Tables 4 and 5). Collectively, these genome assemblies represent a high-quality and comprehensive genomic resource for tetraploid wheat.

### Pangenome construction and analysis

**Fig. 2:**
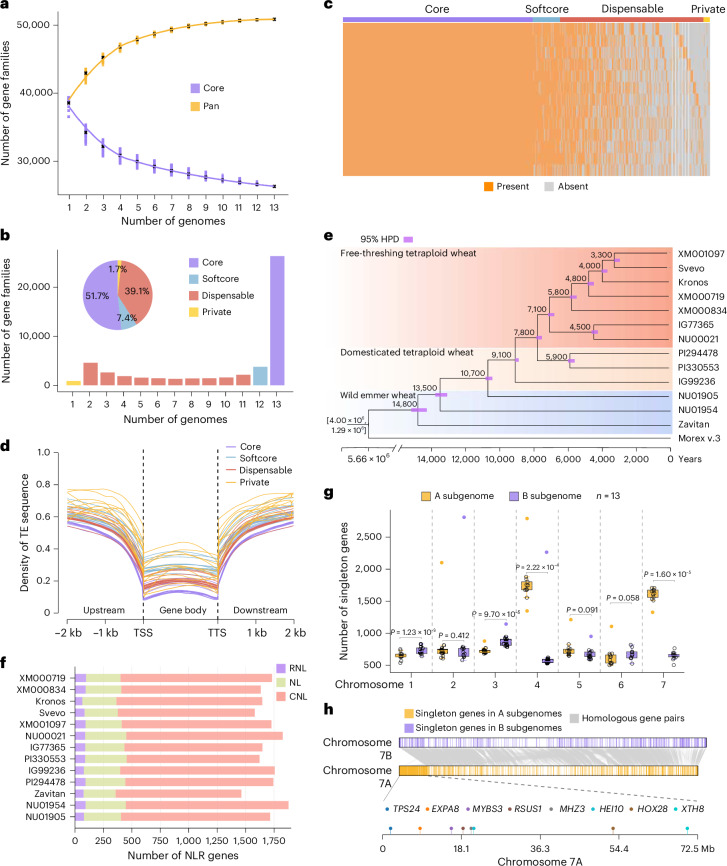
Pangenome analysis of 13 tetraploid wheat accessions. **a**, Pan-core gene family curve in representatives. **b**, Numbers of core, softcore, dispensable and private gene families identified in the pangenome. The histogram shows the frequency of gene families and the pie chart depicts the proportions of core, softcore, dispensable and private gene families. **c**, Presence and absence information of all gene families. **d**, Density of TE sequences in gene bodies and 2-kb flanking regions. **e**, Phylogenetic tree of 13 tetraploid wheat accessions with barley (Morex v.3) as the outgroup. The timeline at the bottom is labeled in descending order, marking key time points from 5.66 million years ago (Ma) to present. **f**, Gene numbers of three NLR subfamilies in 13 tetraploid wheats. **g**, Number of singleton genes in each genome. Statistical tests were conducted using two-sided Mann–Whitney *U*-test, with a Bonferroni-corrected significance threshold (*n* = 13). Center line, median; box lower and upper edges, 25% and 75% quartiles, respectively; whiskers, 1.5× interquartile range (IQR); colored dots, outliers. **h**, Singleton genes on chromosomes 7A and 7B in cv. Kronos. CNL, coiled-coil-domain-containing CC–NBS–LRR-type genes; HPD, highest posterior density interval; NL, NL-type gene that contains at least one NBS (nucleotide-binding site) and LRR (leucine-rich repeat) domain; RNL, RESISTANCE TO POWDERY MILDEW 8 (RPW8)-domain-containing RPW8–NBS-LRR-type genes. Source data

Presence/absence variation (PAV) of genes can result in variable phenotypes. We evaluated accession representativeness by profiling core and pangenome gene dynamics with incremental genome addition, confirming that the tetraploid wheat pangenome approached a plateau (Fig. 2a). We clustered the genes and defined 50,787 distinct gene families. Among them, 26,267 core gene families (51.7%, 64,893 genes per accession) existed in all 13 accessions; 3,770 softcore gene families (7.4%, 6,077 genes per accession) were present in 12 accessions; 19,879 dispensable gene families (39.1%, 11,383 genes per accession) occurred in 2–11 accessions; and 871 private gene families (1.7%, 147 genes per accession) were specific to a single accession (Fig. 2b,c).

Furthermore, 90.81% of the core genes had conserved protein domains, much higher than that of the softcore (88.67%), dispensable (85.11%) and private genes (85.27%) (Supplementary Fig. 9a). A *K*_a_/*K*_s_ analysis also showed that core (median value of *K*_a_/*K*_s_ 0.28) genes underwent stronger purifying selection than softcore (median value of *K*_a_/*K*_s_ 0.36), dispensable (median value of *K*_a_/*K*_s_ 0.42) and private (median value of *K*_a_/*K*_s_ 0.51) genes (Supplementary Fig. 9b). In addition, TE densities within and flanking private, dispensable and softcore genes were higher than that for core genes, indicating that the PAV of these genes were more likely to be related to TE activity^16,17^ (Fig. 2d and Supplementary Fig. 9c). Enrichment analysis showed that core genes were over-represented for certain fundamental biological functions, such as ATP binding (GO:0005524), DNA integration (GO:0015074) and protein glycosylation (GO:0006486). In contrast, softcore, dispensable and private genes were enriched in specific pathways, including root development (GO:0048364), shoot system development (GO:0048367), response to oxidative stress (GO:0034599), recognition of pollen (GO:0048544), endosperm development (GO:0009960) and cadmium ion transmembrane transporter activity (GO:0015086) (Supplementary Fig. 10). This analysis reveals extensive diversity in the gene space of tetraploid wheat, providing a foundation for dissecting the genetic architecture underlying trait variation.

Pangenomes enable tracing of evolutionary histories and genetic divergence among subspecies. Phylogenetic analysis was first performed using 842 single-copy genes present in 13 tetraploid wheat genomes, with barley (Morex v.3) as the outgroup. All accessions were classified into three major subpopulations, including wild emmer wheat, domesticated tetraploid wheat and free-threshing tetraploid wheat (Fig. 2e). Divergence time estimation revealed that domesticated tetraploid wheat diverged from wild emmer wheat approximately 10,700 years ago and free-threshing tetraploid wheat diverged from domesticated tetraploid wheat around 7,800 years ago (Fig. 2e), consistent with published studies^18–23^. High-quality de novo assemblies of tetraploid wheat genomes enabled a more detailed investigation of the close ancestor for bread wheat. Integration of 34 representative bread wheat accessions found that the A and B subgenomes of these hexaploids were genetically closer to that of free-threshing tetraploid wheat, with the closest relationships to Persian wheat (*Triticum carthlicum*) and Ethiopian wheat (*Triticum aethiopicum*; Supplementary Fig. 11).

Dynamic expansion and contraction of gene families are crucial evolutionary drivers underlying crop environmental adaptation. We characterized these variations across 13 tetraploid wheat genomes. In the transition from wild emmer to domesticated tetraploid wheat, 561 gene families expanded and 340 contracted. Additionally, 675 gene families exhibited distinct changes between domesticated and free‑threshing tetraploid wheat (Supplementary Table 6). Disease-resistance genes generally show substantial changes in copy number^24,25^ and several of these have been identified in tetraploid wheat and genetically validated in bread wheat^9,11^. Enrichment analysis revealed that disease resistance-related genes were enriched in contracted gene families from domesticated tetraploid wheat to free-threshing tetraploid wheat (Supplementary Fig. 12). We further analyzed nucleotide-binding sites and leucine-rich-repeat^26,27^ (NLR, also known as NBS–LRR) genes. An average of 1,687 NLR genes were identified per genome, wherein coiled-coil domain-containing CC–NBS–LRR-type (CNL) genes were the most abundant, followed by NL genes (only including at least one NBS and LRR domain) and RESISTANCE TO POWDERY MILDEW 8 (RPW8)-domain-containing RPW8–NBS–LRR-type (RNL) genes (Fig. 2f, Supplementary Fig. 13a and Supplementary Table 7). This pattern is similar to that observed in *Panicum hallii* and rice (*Oryza sativa*) but different from the observations in Brassicaceae family species^26–29^. Additionally, the number of predicted NLR genes in the B subgenomes (56%) was greater than that in A subgenomes (44%).

To investigate the diversity of NLR genes across tetraploid wheat subspecies, we constructed a pan-NLRome containing 308 core, 66 softcore, 435 dispensable and 12 private gene families (Supplementary Figs. 13b,c). The large proportion of private and dispensable NLR genes indicated high diversity among the subspecies. Upon comparing bread wheat NLR genes to the tetraploid pan-NLRomes, 970 gene families were absent from the hexaploid wheat genomes (Supplementary Fig. 13d,e), suggesting that tetraploid wheats harbor highly dynamic NLR gene sets that represent valuable disease-resistance gene resources for hexaploid wheat^30,31^.

### Subgenome asymmetry in tetraploid wheat

Pangenome data enable characterization of asymmetric evolution between subgenomes. We observed marked differences between A and B tetraploid wheat subgenomes, including chromosome length, TE distribution and gene abundance (Supplementary Fig. 14). To further characterize the asymmetric evolution across all 13 genomes, we identified the reciprocal best hit, single-side best hit and singleton (no corresponding syntenic homologs gene between subgenomes) genes between A and B subgenomes (Supplementary Table 8). On average, each genome contained 12,125 singleton genes (6,962 in A and 5,163 in B) (Fig. 2g). Analysis of singleton distributions revealed differences in gene counts between A and B subgenomes on chromosomes 1, 3, 4 and 7 in both domesticated tetraploid wheat and free-threshing tetraploid wheat, with disparities particularly pronounced on chromosomes 4 and 7. Subgenome-specific singleton genes were enriched in the terminal regions of the long arm of chromosome 4A (4AL) and the short arm of chromosome 7 (7AS), as observed in all the assemblies, suggesting that the 7BS–4AL translocation predates the subspeciation of tetraploid wheat^32,33^ (Supplementary Fig. 15). This ancestral rearrangement likely amplified subgenome divergence by increasing the number of singleton genes. Functional annotation revealed that those singletons include gene for disease resistance, such as *TPS24*, *chi11*, *Pht1* and *SIK1*; and grain development, for example *RSUS1*, *MHZ3*, *HEI10* and *DDM1a* (Fig. 2h and Supplementary Table 9), suggesting that the translocation potentiated subgenome-specific evolution and diversification in polyploid wheat.

### Structural variation among tetraploid wheat

**Fig. 3:**
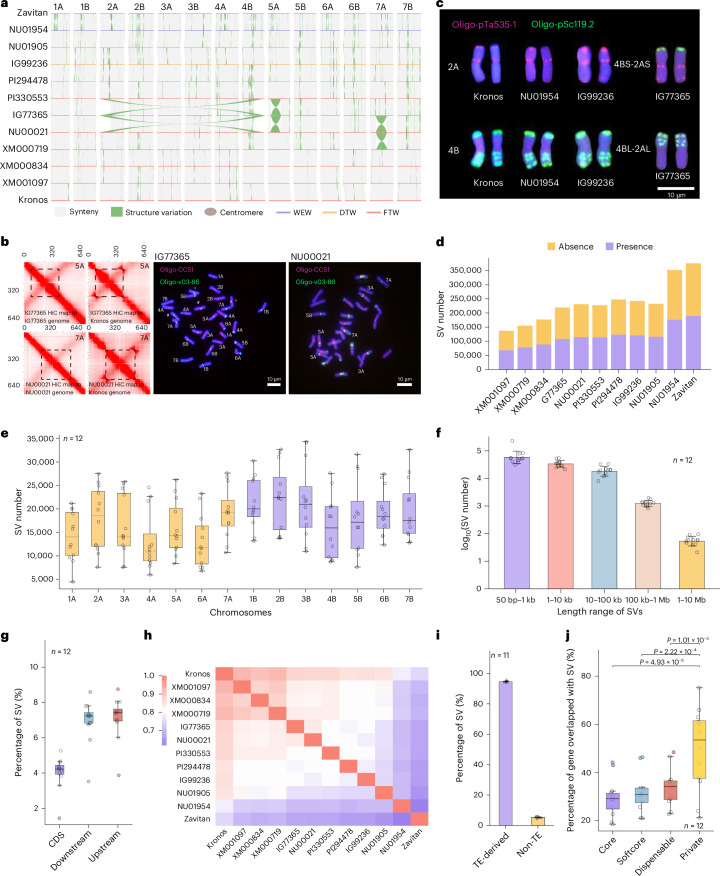
Structural variants across tetraploid wheat genomes. **a**, Whole-genome synteny of the 12 newly assembled genomes. **b**, Hi-C (left) and fluorescence in situ hybridization (right) assays confirm the inversion of the near-centromeric region on chromosome 5A of accession IG77365 and on chromosome 7A of accession NU00021. The axes on the heatmap represent megabases. Fluorescence in situ hybridization assays were repeated twice. **c**, Fluorescence in situ hybridization for chromosomes 2A and 4B of accession IG77365. **d**, Number of SVs (deletions and insertions) observed in each accession, using the Kronos genome as a reference. **e**, Number of SVs in each chromosome across 11 newly assembled and Svevo genomes (*n* = 12), with Kronos as reference. **f**, Distribution of SV length across 11 newly assembled and Svevo genomes (*n* = 12), with Kronos as reference. Error bars are mean ± s.e.m. **g**, Percentage of SVs located in different regions of annotated protein-coding genes (*n* = 12). **h**, Heatmap of IBS values based on SVs among 12 genomes. **i**, The proportion of TE-related SVs and non-TE SVs in tetraploid wheat (*n* = 11). Error bars are mean ± s.e.m. **j**, Number of core, softcore, dispensable and private genes that overlap with SVs (*n* = 12). Statistical significance was determined by one-way analysis of variance followed by Fisher’s least-significant difference multiple-comparisons tests. Center line, median; box lower and upper edges, 25% and 75% quartiles, respectively; whiskers, 1.5× IQR; colored dots, outliers (**e**, **g** and **j**). DTW, domesticated tetraploid wheat; FTW, free-threshing tetraploid wheat; WEW, wild emmer wheat; CDS, coding sequence. Source data

We further characterized genomic diversity at base-pair resolution by comparing 12 newly assembled genomes and found an average of 83.51% matched sequences and as high as 12.58% of gap sequences (Supplementary Fig. 16). By visualizing the genome alignments (Fig. 3a), we observed three large karyotype variations, including a translocation between chromosomes 2A and 4B of accession IG77365 (*T. aethiopicum*), an 317.34-Mb inversion on chromosome 5A of accession IG77365^34^ and a 355.54-Mb inversion on chromosome 7A of accession NU00021 (*T. carthlicum*). These large karyotype variations were verified by fluorescence in situ hybridization and Hi-C heatmaps (Fig. 3b,c, Supplementary Note 3 and Supplementary Figs. 17 and 18). On average, each accession had 233,976 SVs, including 116,990 deletions and 116,986 insertions (Fig. 3d and Supplementary Table 10). The number of SVs located in B subgenomes was larger than in A subgenomes, underscoring asymmetry across subgenomes (Fig. 3e). PCR validation of 30 randomly selected SVs validated the accuracy of these assignments (Supplementary Fig. 19 and Supplementary Table 11).

These SVs account for 11.29% on average of the genome sequences (ranging from 0.78 Gb to 1.77 Gb), with an average length of 3,450 base pairs (bp) (Fig. 3f). We also observed that on average 6.01% of all SVs were located upstream or downstream of protein-coding genes and 3.31% of SVs overlapped with coding regions. These overlapping SVs might have substantial functional relevance by affecting gene structure or expression (Fig. 3g). Identity-by-state (IBS) values based on SVs also showed higher potential lineage homology among free-threshing tetraploid wheat but lower in wild emmer wheat (Fig. 3h). We observed that an average of 94.7% of SVs overlapped with TEs (Fig. 3i), which is consistent with the hypothesis that most SVs are derived from TE activation^35^. Changes in SV number were also observed among core, softcore dispensable and private genic regions (Fig. 3j), demonstrating that SVs confer gene PAVs across different accessions. The extensive sequence diversity and SV landscape in tetraploid wheat preclude accurate variant detection using a single reference genome.

### Population structure and subspecies introgression

**Fig. 4:**
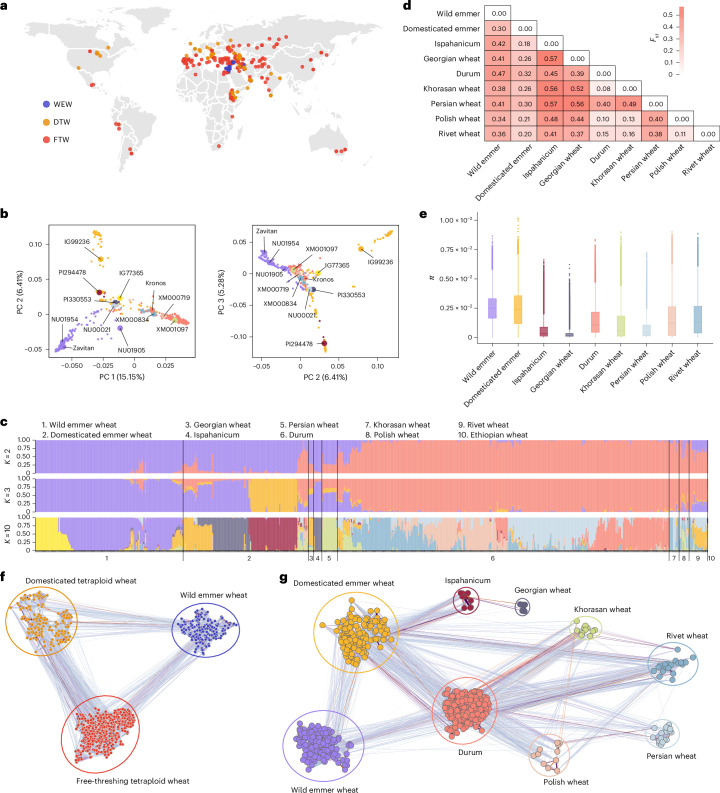
Population structure and evolutionary analysis. **a**, Geographic distribution of 736 sequenced accessions. **b**,**c**, Principal component (PC) analysis (**b**) and ancestry coefficient analysis (**c**) of 736 sequenced accessions. The optimal *K* value is 10. In **b**, the de novo assembled accessions are highlighted. **d**, Population divergence (*F*_st_) among different tetraploid wheat subspecies. **e**, Nucleotide diversity of nine subspecies (*n* = 736). Center line, median; box lower and upper edges, 25% and 75% quartiles, respectively; whiskers, 1.5× IQR. **f**,**g**, Evolution networks for three major subpopulations (**f**) and nine subspecies (**g**) of tetraploid wheat. Clustered phylogenetic consensus genotype networks of 1,000 maximum-likelihood tree topologies were inferred from a repeated random-haplotype sampling procedure including heterozygous sites. Nodes represent individual genotypes and are color-coded by subspecies. Edges represent minimal evolutionary distances in the repeated random-haplotype sampling trees deduced by the minimum spanning tree algorithm and are color-coded based on distance size. Lines with edge weights lower than the average were removed. The map in **a** was plotted using the R package rworldmap (GPL v.2 license, https://github.com/AndySouth/rworldmap), with base map data from Natural Earth (http://www.naturalearthdata.com).

By integrating the linear reference-genome sequences of Kronos and all SVs identified from the 12 tetraploid wheat genomes, including the published Svevo genome^13^, a comprehensive graph-based pangenome for tetraploid wheat was constructed. Further, we constructed a genetic-variation map by deep whole-genome sequencing of 736 accessions (621 were generated in this study and 115 were publicly available^8^), resulting in 97.11 million single nucleotide polymorphisms (SNPs), 4.36 million small indels (indels, length <50 bp) and 0.59 million SVs (length ≥50 bp) (Fig. 4a and Supplementary Tables 12 and 13), dramatically increasing the availability of tetraploid wheat genomic resources. Phylogenetic analysis suggests that these 736 accessions could be divided into three major subpopulations, namely wild emmer wheat, domesticated tetraploid wheat and free-threshing tetraploid wheat (Supplementary Fig. 20). Principal component analysis and ancestry coefficient analysis also supported the results of three major subpopulations (Fig. 4b,c).

We observed a high degree of differentiation between Ispahanicum (*Triticum ispahanicum*), Georgian (*Triticum karamyschevi*), Persian (*T.* *carthlicum*) tetraploid wheat and other subspecies, suggesting geographic isolation or ecological adaptation (Fig. 4d). Analysis of nucleotide diversity (*π*) showed that wild emmer wheat and domesticated emmer wheat (*T. dicoccon*) had higher genetic diversity than the other subspecies; the Ispahanicum and Georgian wheat had the lowest diversity (Fig. 4e). We further investigated the evolution and introgression of those subspecies via an evolution network, with the resulting topology supporting a two-step evolution process for tetraploid wheat (Fig. 4f), from wild emmer wheat to domesticated and free-threshing tetraploid wheat. There are fewer edges for Ispahanicum and Georgian accessions in the network, suggesting less potential introgression from them to free-threshing tetraploid wheats and that they made a smaller contribution to the improvement of cultivated tetraploid wheat (Fig. 4g and Supplementary Note 4). Gene-flow analysis suggested more potential introgression regions arising from wild emmer wheat moved into Persian wheat than other free-threshing tetraploid wheats (Supplementary Note 5 and Supplementary Fig. 21). Except for Persian wheat, the proportion of genome introgression among free-threshing tetraploid wheats was larger than in other types of tetraploid wheats, with an average of 0.36 (Supplementary Table 14). Introgression introduces new genetic variation and contributes to the genomic diversity (Supplementary Figs. 22 and 23a). Genes within the inferred introgression regions showed enrichment in certain pathways, including response to nitrate (GO:0010167) and plant-type cell wall organization (GO:0009664) (Supplementary Table 15). The *GW2* gene is a key determinant of grain weight in rice and bread wheat^36–38^. *TrdurKRO6B01G046970*, an ortholog of *TaGW2-B* from bread wheat, is in the putative introgression region (*f*_d_ ≈ 0.44) on chromosome 6B that originated from wild emmer wheat and moved into domesticated emmer wheat (Supplementary Fig. 23b). These results highlight the potential of introgression for improving agricultural traits.

### Genetic variation underlying domestication and improvement

**Fig. 5:**
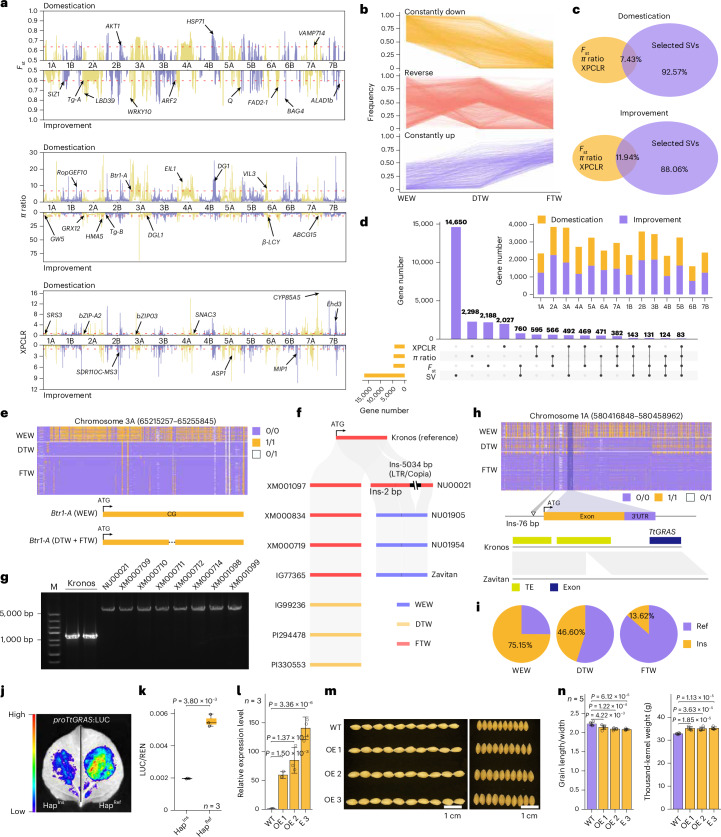
Structural variants reveal selection footprints and multiple alleles of *Btr1*-*A*. **a**, *F*_ST_, nucleotide diversity ratio (domestication, *π*_WEW_/*π*_DTW_; improvement, *π*_DTW_/*π*_FTW_) and cross-population composite-likelihood ratio approach (XPCLR) analyses of selection. Dashed lines indicate genome-wide thresholds for selection signals. The upper section of each plot represents the domestication analysis; the lower section denotes the improvement analysis. **b**, Frequency pattern of selection-related SVs. The orange and purple lines represent the gradual decrease and increase in SV frequency during the evolutionary process, respectively. **c**, Overlap of selection signals identified via SV-based, SNP-based and indel-based methods. **d**, Overlap of selected genes across four different methods. **e**, Variation heatmap within 20-kb upstream and downstream of *Btr1-A*. **f**, *Btr1-A* synteny across 12 genomes, wherein *Btr1-A* has a single coding region. **g**, PCR verification of the 5,034-bp TE insertion in the *Btr1-A* coding region found in eight Persian wheats. **h**, Variation heatmap within 20-kb upstream and downstream of *TtGRAS-A*. UTR, untranslated region. **i**, Allele frequency of the selected SV (at position 580,435,572 on chromosome 1A) upstream of *TtGRAS-A* among three subpopulations. Ref, reference (Kronos); Ins, insertion. **j**,**k**, Chemiluminescence (**j**) and LUC/REN (**k**) for dual-reporter assay of Hap^Ref^ and Hap^Ins^ *TtGRAS-A* promoter variants (*n* = 3) transiently expressed in *N. benthamiana* leaves. Statistical significance was determined by two-sided *t*-tests. Center line, median; box lower and upper edges, 25% and 75% quartiles, respectively; whiskers, 1.5× IQR; colored dots, outliers. **l**, *TtGRAS-A* expression in transgenic wheat lines cv. Fielder with heterologous *TtGRAS-A* expression with three independent biological replicates. Error bars are mean ± s.e.m. **m**,**n**, Grain phenotypes (**m**) and quantification (**n**) of grain length/width and thousand-kernel weight for transgenic wheat lines heterologously expressing *TtGRAS-A* in the cv. Fielder wild-type background (WT) with five independent biological replicates. Error bars are mean ± s.e.m. Statistical significance was determined by one-way analysis of variance followed by Fisher’s least-significant difference multiple-comparisons tests (**l**,**n**). Source data

To detect putative selection footprints in tetraploid wheat genomes, we compared genetic diversity to identify bottlenecks during domestication (wild emmer versus domesticated tetraploid wheat) and improvement (domesticated versus free-threshing tetraploid wheat) (Supplementary Fig. 24). Wild emmer wheat exhibited the highest genetic diversity (*π* = 2.54 × 10^−3^), followed by domesticated tetraploid wheat (*π* = 2.44 × 10^−3^) and free-threshing tetraploid wheat (*π* = 1.62 × 10^−3^), indicating a sharp decline in genetic diversity under artificial selection. From a combined SNP- and indel-based selective-sweep analysis, we identified 0.93-Gb and 1.17-Gb selected regions corresponding to domestication and improvement processes, which contained 4,675 and 7,210 genes, respectively (Fig. 5a and Supplementary Table 16). Linkage-disequilibrium analysis revealed that haplotype decay for variants including SVs, SNPs and indels occurred more rapidly than for SNPs alone, suggesting that SVs harbor substantial hidden genetic variation (Supplementary Fig. 25). We compared SV frequencies between wild and landrace accessions to identify putative selected SVs during domestication (Fig. 5b) and between landrace and cultivars for possible selected SVs during improvement. This identified 157,468 SVs associated with 15,642 genes and 151,898 SVs associated with 15,001 genes (Fig. 5b and Supplementary Table 17) exhibiting selection signals during domestication and improvement, of which 133,607 SVs were subjected to selection during both domestication and improvement. Several well-known domesticated genes in the Triticeae tribe were also found, such as *Rht-B1*, *GW8-B1* and *Yr15*^39–41^. Only 7.48% and 11.97% of selection SVs, during domestication and improvement, respectively, were shared by SNP- and indel-based selection regions (Fig. 5c). A total of 25,379 non-redundant selected genes were identified, of which 2,204 were shared between SNP/indel and SV analysis (Fig. 5d), suggesting the additional power of SVs for selection footprint identification. Enrichment analysis showed selected genes were mainly involved in biological processes, including response to heat (GO:0009408), carbohydrate metabolic process (GO:0005975), positive regulation of long-day photoperiodism and flowering (GO:0048578) and carbon utilization (GO:0015976) (Supplementary Table 18).

Non-brittle rachis phenotype of wheat is a key trait for domestication. Mutations in *Btr1*-*A* and *Btr1-B* are chiefly responsible for the non-brittle rachis phenotype in tetraploid wheat^12^. Several loss-of-function mutations of *Btr1-A* have been observed in domesticated einkorn^8^, barley^42^ and tetraploid wheat^12^. Consistent with ref. ^12^, we observed the hallmark 2-bp deletion introducing a frame shift in the *Btr1-A* coding region of cultivated tetraploids (Fig. 5e). The Persian domesticated tetraploid accession NU00021, with the non-brittle-rachis phenotype, lacks this translation-termination mutation, but has a 5,043-bp LTR/Copia-type TE insertion in the coding region based on the tetraploid wheat pangenome, which likely resulted in loss of its function (Fig. 5f). We also found that both LTRs within this element were identical, hinting that the LTR insertion likely occurred in a recent^43^ phylogenetic branch of the tetraploid wheat population. Analysis of *Btr1-A* haplotypes across the 736 accessions indicated all sampled Persian accessions contain this LTR insertion (Fig. 5g). These observations suggest an independent loss-of-function led to convergent evolution of the non-brittle-rachis phenotype^44^. The gene flow and evolution networks suggest that domesticated tetraploid wheat may have multiple origins and Persian wheat may have undergone a different domestication path and history.

In addition, we identified a 76-bp SV-specific locus underlying selection, which is located 1.28-kb upstream of the translational start codon of a *GRAS* gene, assigned *TtGRAS-A* (*TrdurKRO1A01G076930*). The frequency of the SV-insertion allele decreases from wild (0.75) to domesticated (0.47) and free-threshing tetraploid wheat (0.14) (Fig. 5h,i). This SV on chromosome 1A is statistically significantly associated with thousand-kernel weight and the ratio of grain length/grain width (Supplementary Fig. 26). To examine the regulatory role of this SV, a transient dual-luciferase reporter assay in *Nicotiana benthamiana* leaves suggested the *TtGRAS-A* SV-deletion allele has higher expression than the SV-insertion allele (Fig. 5j,k). Heterologous expression of *TtGRAS-A* in hexaploid wheat cv. Fielder from the strong, constitutive *Ubiquitin* promoter increased grain size, thousand-kernel weight and effective tiller number per plant (Fig. 5l–n and Supplementary Fig. 27). These results indicate that the long deletion upstream of *TtGRAS-A* may enhance its expression and thereby improve yield-related traits.

### Genetic dissection of 32 traits

We phenotyped 373 resequenced tetraploid wheat accessions for 32 agronomically important traits covering key breeding targets: 7 plant-architecture, 8 grain-related and 17 abiotic stress-related traits—to identify variants associated with them (Supplementary Tables 19 and 20). Heritability estimates indicated that SVs based on the graph-based pangenome can explain additional phenotypic variance (Supplementary Fig. 28 and Supplementary Table 21). To dissect the genetic components underlying these traits, we performed SV-based and SNP + indel-based GWAS. A total of 287 loci were associated with 32 traits. These loci contain 783 unique protein-coding genes (Supplementary Fig. 29 and Supplementary Table 22). Of these, 34 SNP + indel-based loci were shared with SV-based approaches and 44 loci were specifically identified by SVs. For instance, a 82-bp SV in the intron of *TrdurKRO3A01G024420* was associated with grain length/width, of which the rice ortholog *RDR4* regulates spike length and grain plumpness^45^ (Supplementary Fig. 30a). Additionally, *TrdurKRO2A01G031640*, an ortholog of rice *SNAC3*^46^, is located 99.3-kb downstream of an indel locus associated with root length under simulated drought treatment (Supplementary Fig. 30b).

### *HAT14-B*^*Hap*1^ enhances spikelet numbers and grain size

**Fig. 6:**
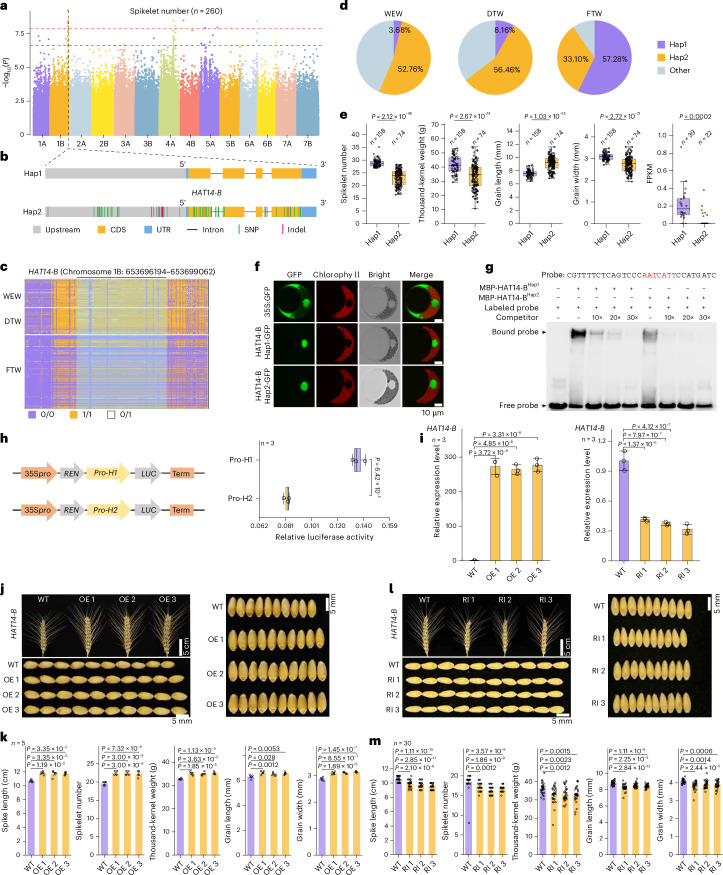
The HD-ZIP transcription factor HAT14-B positively regulates spikelet number and grain size. **a**, Indel-based GWAS of 260 resequenced accessions with spikelet number phenotype. The horizontal lines indicate the two-sided Bonferroni-corrected genome-wide statistical significance threshold (*P* = 2.37 × 10^−7^ (*α* = 1) and *P*= 1.83 × 10^−8^ (*α* = 0.05)). **b**, Gene structure and haplotype analysis of *HAT14-B*. **c**, Variation heatmap from 2-kb upstream to 2-kb downstream of *HAT14-B*. **d**, Proportions of WEW, DTW and FTW accessions with the two *HAT14-B* haplotypes. **e**, Box plots for spikelet number, thousand-kernel weight, grain length, grain width and *HAT14-B* expression for accession with *HAT14-*B^*Hap1*^ or *HAT14-*B^*Hap2*^. Statistical significance was determined by two-sided *t*-tests. FPKM, fragments per kilobase of transcript per million mapped reads. **f**, Subcellular localization of HAT14-B^Hap1^ and HAT14-B^Hap2^ fused to GFP in transiently transgenic wheat protoplasts with twice repeats. **g**,**h**, EMSA analysis of probes bearing the AT-containing motif (**g**) and promoter activity via dual-reporter assays (**h**) of *HAT14-B*^*Hap1*^ and *HAT14-B*^*Hap2*^. Values are mean ± s.e.m. from three independent biological replicates and EMSAs were repeated twice. Statistical significance was determined by two-sided *t*-tests. **i**, *HAT14-B* expression in transgenic wheat lines with heterologous *HAT14-B* expression (left) and RNAi knock-down lines (right), all in the wild-type cv. Fielder background. Values are mean ± s.e.m. from three independent biological replicates. Statistical significance was determined by one-way analysis of variance followed by Fisher’s least-significant difference multiple-comparisons tests. **j**,**k**, Representative spike and grain phenotypes (**j**) and quantification (**k**) of spike length, spikelet number, thousand-kernel weight, grain length and grain width for transgenic wheat lines heterologously expressing *HAT14-B*^*Hap1*^ in the wild-type cv. Fielder background. Values represent the mean ± s.e.m. from five independent biological replicates. **l**,**m**, Representative spike and grain phenotypes (**l**) and quantification (**m**) of spike length, spikelet number, thousand-kernel weight, grain length and grain width for transgenic *HAT14* RNAi lines in the wild-type cv. Fielder background. Values represent the mean ± s.e.m. from 30 independent biological replicates. Statistical significance was determined by one-way analysis of variance followed by Fisher’s least-significant difference multiple-comparisons test (**k**,**m**). Center line, median; box lower and upper edges, 25% and 75% quartiles, respectively; whiskers, 1.5× IQR; colored dots, outliers. OE, heterologous-expression lines; RI, RNAi lines; WT, wild type. Source data

Spikelet number is a critical agronomic trait that is closely associated with wheat grain yield. A 15-bp indel (*P* = 6.17 ×10^−10^) on chromosome 1B associated with spikelet number was found in exon 4 of *TrdurKRO1B01G083560* (Fig. 6a). The gene is an ortholog of the *Arabidopsis* homeodomain-leucine zipper (HD-ZIP) transcription factor *HAT14* (Supplementary Fig. 31) and herein designated as *HAT14-B*. This gene has two main haplotypes in tetraploid wheat, *HAT14-B*^*Hap1*^ and *HAT14-B*^*Hap2*^ (Fig. 6b and Supplementary Fig. 32). Haplotype-frequency analysis showed that *HAT14-B*^*Hap1*^ is present in 5.56% (6 of 163) of wild emmer wheat, 11.11% (12 of 147) of domesticated tetraploid wheat and 61.61% (244 of 426) of free-threshing tetraploid wheat accessions after removing the heterozygous sites (Fig. 6c,d), indicating a strong selection footprint from hulled tetraploid wheat to free-threshing tetraploid wheat (Fisher’s exact test, *P* < 2.2 × 10^−16^). *HAT14-B*^*Hap1*^ accessions have more spikelet number, higher thousand-kernel weight, higher grain width but lower grain length compared to *HAT14-B*^*Hap2*^ (Fig. 6e). In addition, *HAT14-B*^*Hap1*^ expression in spikes is statistically significantly higher than *HAT14-B*^*Hap2*^ (Fig. 6e). Variation in the *HAT14-B* coding region did not obviously alter the nuclear localization of the corresponding protein products when fused to GFP (Fig. 6f). HD-ZIP transcription factors bind as dimers to palindromic DNA sequences containing the AATNATT motif (AT-containing motif)^47^. HAT14-B^Hap2^ DNA-binding ability was attenuated compared to that of HAT14-B^Hap1^ in electrophoretic mobility shift assay (EMSA) experiments (Fig. 6g). Dual-luciferase assays showed relatively high activity from an approximately 2-kb *HAT14-B*^*Hap1*^ promoter fragment compared to the *HAT14-B*^*Hap2*^ promoter fragment (Fig. 6h). These results indicated that Hap1 is the elite allele of *HAT14-B*.

To explore the function of the elite *HAT14-B*^*Hap1*^ allele in spike development, we heterologously expressed its coding region in hexaploid wheat cv. Fielder (Fig. 6i). Transgenic lines had statistically significantly greater spike length, spikelet number, thousand-kernel weight, grain length and grain width compared to Fielder controls (Fig. 6j,k), affirming that the elite *HAT14-B*^*Hap1*^ allele is a robust candidate for regulating spikelet number and grain size. Furthermore, RNA-mediated interference (RNAi) lines against *HAT14-B*^*Hap1*^ had shorter spike length, fewer spikelet number, reduced thousand-grain weight and smaller grains compared to Fielder controls (Fig. 6l,m). However, the *HAT14-B*^*Hap1*^ transgenic and RNAi lines did not exhibit a significant difference in effective tiller number (Supplementary Fig. 33). We demonstrate that *HAT14-B*^*Hap1*^ enhances spikelet number and grain size in wheat. Those discoveries, enabled by the newly generated tetraploid wheat pangenome, underscore its utility for gene discovery and crop improvement.

## Discussion

Tetraploid wheat (*T. turgidum* L., BBAA), the donor of B and A subgenomes of hexaploid bread wheat, possesses abundant genetic and germplasm resources^48^. Tetraploid wheat serves as a vital gene pool with abundant untapped beneficial alleles and loci for improving bread wheat agronomic traits. Nevertheless, efficient genomic molecular breeding and gene editing are limited by insufficient comprehensive in silico resources. Here we performed de novo assembly of 12 high-quality genomes covering all 10 known tetraploid wheat subspecies to support the mining of novel alleles and SVs.

Pangenome analysis revealed substantial genomic diversity in tetraploid wheat subspecies. Phylogenetic analysis using orthologous genes clearly differentiated wild emmer, domesticated tetraploid and free-threshing wheat into separate clades and clarified the evolutionary trajectories of the ten subspecies. Disease resistance genes were enriched in contracted gene families during the transition from domesticated to free-threshing tetraploid wheat. We also identified numerous tetraploid wheat-specific NLR genes, offering valuable new genetic resources for disease-resistance breeding^49^.

Extensive SVs, SNPs and indels further shape the high genomic diversity of tetraploid wheat. Notably, pairwise accessions exhibit an average sequence divergence of 12.58%, reflecting its remarkable genetic variation. Leveraging these high-quality genomes, we constructed a graph-based pangenome. This study reports a comprehensive pangenome of tetraploid wheat (*T. turgidum* L., BBAA) covering all tetraploid wheat subspecies and dissecting their broad genetic diversity. This dataset represents the most complete atlas of tetraploid wheat genomic diversity to date, providing key resources for dissecting evolutionary mechanisms and enriching the genetic reservoir for bread wheat improvement.

Genomic signatures of domestication and improvement frequently offer practical insights for molecular breeding^50,51^. Population evolution and dynamics of tetraploid wheat have been examined, but the evolutionary relationships among different subspecies remain unclear^7,8,18,52^. We characterized the evolutionary history of ten tetraploid wheat subspecies, finding that Ispahanicum and Georgian subspecies bear high genetic differentiation and low introgression with other subspecies, likely due to geographic isolation. Persian wheat is also markedly divergent from other free-threshing tetraploids and harbors a loss-of-function *Btr1-A* allele without the canonical 2-bp deletion^18^, while having a 5,034-bp LTR/Copia insertion in the protein-coding region. A recent study indicates that independent loss-of-function alleles likely emerged in wild emmer populations^44^, highlighting the complexity of crop evolution. These findings underscore the potential of pangenomic approaches to reveal novel domestication variants and provide new targets for accelerated crop improvement and de novo domestication^53^. The development of new wheat varieties is urgently required to adapt to climate change and future planting. Pangenome-driven strategies facilitate the mining of untapped genetic variations for crop breeding. Unlike single-genome references, pangenomes enable comprehensive dissection of genetic mechanisms governing phenotypic diversity. Similar to earlier findings, SVs conferred extra heritability beyond SNPs and indels, highlighting the strengths of pangenome-supported SV-GWAS^54,55^.

Using the tetraploid wheat pangenome and GWAS, we identified the pleiotropic gene *HAT14-B*, which provides an important resource for breeding of high-yielding wheat varieties. The tetraploid wheat pangenome complements existing hexaploid wheat resources and will accelerate the identification of key trait genes as well as facilitate the breeding of improved wheat varieties by tapping into the rich genetic diversity of wheat ancestors.

Overall, this study provides a foundational genomic resource that will accelerate comparative genomics and breeding in wheat and pave the way for the effective deployment of tetraploid wheat diversity in improving wheat.

## Methods

### Plant material

A total of 622 accessions covering all of the ten subspecies belonging to tetraploid wheat were used in this study, representing a worldwide tetraploid wheat germplasm collection (Supplementary Tables 19 and 20 and Supplementary Note 1). We conducted de novo assembly for 12 accessions aiming to cover 10 subspecies from different areas (Supplementary Tables 1 and 12). Among them, three wild emmer accessions were de novo assembled and they harbor high genomic diversity of the wild subspecies, from different regions of Levant, indicated by the principal component analysis.

### Genome assembly

#### Step 1. Contig construction

We used a hybrid approach for initial contig assembly. For combined HiFi and ONT ultra-long reads (length >100 kb, QV > 10), we used both hifiasm (v.0.22.0-r689)^56^ and Verkko (v.2.0)^57^ assemblers. We also used hifiasm (v.0.22.0-r689) to conduct assembly using ONT data independently. Considering the high computational resource cost of NextDenovo (v.2.5.2)^58^ and the large genome size of Kronos, we implemented a chromosome-by-chromosome assembly strategy. This involved aligning ONT data to 14 chromosomes using minimap2 (v.2.24)^59^ (targeting only HiFi-assembled regions), followed by separate NextDenovo (v.2.5.2)^58^ assemblies for each chromosomal ONT subset. To filter organelle genomes, the chloroplast and mitochondrial genomes of *Triticum urartu*, *Triticum monococcum*, *Triticum zhukovskyi*, *T. turgidum* (cv. Svevo), *Triticum timopheevii* and *Triticum macha* were downloaded from the National Center for Biotechnology Information. We used minimap2 (v.2.24)^59^ with -cx asm20 to align the contigs of each species onto the reference organelle genomes. Contigs that are composed of ≥95% organelle sequence and ≤5% divergence were filtered to obtain nuclear genomes.

#### Step 2. Hi-C scaffolding

Chromosomal contigs were integrated through hierarchical scaffolding. We first applied YAHS (v.1.2a.1)^60^ for initial scaffolding of different assembly outputs. Subsequently, RagTag (v.2.1.0)^61^ was used to refine the NextDenovo (v.2.5.2)^58^ assembly results. To address gaps in the primary framework, we extracted two types of sequences: (1) unaligned ONT contigs; and (2) 2-Mb flanking sequences at scaffold gaps. These components underwent dedicated NextDenovo (v2.5.2)^58^ assembly to generate supplementary contig sets.

#### Step 3. Gap filling

The Hi-C scaffold hifiasm-derived hybrid assembly (HiFi + ONT) was designated as the reference framework. We used the genoAli command of AnchorWave (v.1.2.6)^62^ to establish a global collinearity map between an alternative assembly and the reference genome for each chromosome. For gap filling, the interval between each pair of adjacent collinear anchor pairs was systematically evaluated: reference-genome intervals containing gaps while absent from corresponding alternative assembly regions underwent sequence replacement using the contiguous sequence of the alternative assembly. This gap-filling protocol was iteratively applied across four alternative assemblies to maximize gap-closure efficiency.

#### Step 4. Sequence polishing

Three rounds of polishing were performed using NextPolish (v.1.4.1)^63^, exclusively using HiFi reads for error correction.

### Assembled genome evaluation

To evaluate the completeness of each genome assembly, BUSCO (v.5.4.6)^64^ was used with the OrthoDB database poales_odb10 (poales, 2020-09-10). We evaluated the consensus QV and completeness of genome assemblies on the basis of the *k*-mers spectrum of HiFi reads and Illumina whole-genome sequencing reads, using Merqury (v.1.3)^65^ with default parameters. To evaluate assembly continuity, LAI was calculated for each genome assembly using LTR_retriever (v.2.9.0)^66^.

### Repeat annotation

Tandem repeats of all assemblies were identified using Tandem Repeats Finder (TRF) (v.4.09)^67^. We also identified candidate telomeres on scaffolds based on TRF (v.4.09) results using the plant telomeric-repeat motif (CCCTAAA)*n* or (TTTAGGG)*n*. TEs were identified by the Extensive de novo TE Annotator (EDTA) (v.2.0.1)^68^. In brief, structure-intact TEs (including LTR-RT, DNA transposon, Helitron) predicted by EDTA (v.2.0.1)^68^ were firstly softmasked.

The unknown TE sequences in the fragmented TE library predicted by EDTA (v.2.0.1)^68^ were classified using the Transposable Element Representation Learner (TERL)^69^. The known and classified fragmented TE library was then used to mask the genome with RepeatMasker (http://repeatmasker.org/; v.4.1.1). Finally, we merged all TE annotations, removed redundancy and obtained the final TE annotation and the softmasked genome of each assembly.

### Genome annotation

To minimize discrepancies arising from the use of different annotation tools, we used a unified annotation pipeline for all 12 genomes assembled de novo. The annotation integrated evidence from RNA-seq, Iso-seq, homologous protein alignments and ab initio gene prediction.

Stranded RNA-seq reads were aligned to softmasked genome using STAR^70^ (v.2.7.10b; --outSAMattrIHstart 0 --outSAMstrandField intronMotif --alignIntronMin 20 --alignIntronMax 20000) and transcripts were assembled using StringTie^71^ (v.3.0.0; -m 150 -t -f 0.3 -rf). Iso-seq data were processed by first mapping redundant isoforms to the softmasked genome using pbmm2 (https://github.com/PacificBiosciences/pbmm2; v.1.17.0), followed by collapsing into transcripts using Iso-seq (https://github.com/PacificBiosciences/IsoSeq; v.4.3.0). Transcript loci were predicted by GMAP^72^ (v.2024-11-20; -L 10000000). For coding-region prediction, transcript assemblies from RNA-seq and Iso-seq were merged using StringTie^71^ (v.3.0.0; -m 150) and passed to TransDecoder (https://github.com/TransDecoder/TransDecoder; v.5.7.1), resulting in transcriptome-derived gene annotations.

For protein homology evidence, gene structures from a diverse panel of species, including *Arabidopsis thaliana* (TAIR10), *Sorghum bicolor* (v.3), *Zea mays* (B73 NAM V5), *O. sativa* (IRGSP-1.0.60), *Secale cereale*, *Brachypodium distachyon* (v.3.0), *Aegilops speltoides*, *Hordeum vulgare*, *T. aestivum* (IWGSC v2.1), *T. dicoccoides*, *T. turgidum* (Svevo.v1) and *T. urartu* were projected onto softmasked genomes using miniprot (v.0.12)^73^.

Ab initio gene predictions were produced with Augustus^74^ (v.3.5.0; --softmasking = on –genemodel = complete --alternatives-from-evidence = on) and GeneMark-ET^75^ (v.4.72_lic). We merged the transcriptome-derived gene annotations and homology evidence using the cuffcompare program from cufflinks^76^ (v.2.2.1) and StringTie^71^ (v.3.0.0) and passed the resulting GTF file to TransDecoder (v.5.7.1) to generate training data for Augustus^74^ (v.3.5.0). Augustus^74^ (v.3.5.0) gene prediction was performed using a model specifically trained from the software and three hints files generated by the above-mentioned RNA-seq, Iso-seq and homology-based predictions. GeneMark-ET^75^ (v.4.72_lic) was used with the option -ET and intron coordinates were calculated using the above-mentioned RNA-seq alignments.

All the gene evidence from transcriptomics, protein alignments and ab initio predictions was integrated using EvidenceModeler^77^ (v.2.1.0; --segmentSize 100000 --overlapSize 10000), with weights assigned using the parameters in ref. ^78^, transcript (merged from RNA-seq and Iso-seq, 10), homology (8), Augustus (5) and GeneMark (2). The resulting annotations were refined through two rounds of PASA (https://github.com/PASApipeline/PASApipeline; v.2.5.3).

Gene models were classified as high-confidence (HC) or low confidence (LC) genes according to criteria used by the IWGSC (https://urgi.versailles.inra.fr/download/iwgsc/IWGSC_RefSeq_Annotations/v1.0/iwgsc_refseqv1.0_README.pdf) and ref. ^79^. DIAMOND (v.2.1.8)^80^ was used to compare predicted protein sequences against three curated datasets: UniMag (38,182 reviewed Magnoliopsida proteins, downloaded from Uniprot (SwissProt), June 2025), UniPoa (2,848,452 Poaceae proteins downloaded from Uniprot (SwissProt and trEMBL), June 2025)^81^ and PTREP (https://trep-db.uzh.ch/index.php; Rel-19, June 2025). Protein-encoding gene models were considered complete when translational start and stop codons were present. A HC protein sequence is complete with a hit in the UniMag database (HC1), or with hits in UniPoa and not in TREP (HC2). An LC protein sequence is incomplete and has a hit in the UniMag (LC1) or complete gene models with no hits to any of the three databases (LC2) or incomplete gene models with hits in Poaceae protein but no hits in the TE database TREP (LC3) or incomplete gene models with no hits to any of the three databases (LC4). TREP genes have no hits to UniMag proteins but with hits to TREP entries. We also promoted gene models with intron chains supported by Iso-seq to HC (HC3). InterProScan (v.5.73)^82^ was used to predict potential protein domains and perform gene ontology annotations for HC genes of each accession with -goterms -iprlookup -pa -dp.

### Centromeric region identification

Adapters and low-quality reads in ChIP–seq data for 12 accessions were removed using Trimmomatic (v.0.39)^83^ with ILLUMINACLIP: adapter.fa:2:30:10 MINLEN:36 SLIDINGWINDOW: 4:20. Quality-controlled reads were aligned to each genome using Bowtie2^84^ (v.2.5.1; --very-sensitive --no-mixed --no-discordant -k 10 -X 1000) and were further filtered by SAMtools^85^ (v.1.10; -F 1804 -f 2 -q 30). MACS3 (v.3.0.1)^86^ was used to call CENH3 ChIP–seq peaks using input as a control with -f BAMPE. Adjacent CENH3-enriched regions within 1,000 kb were merged and the most enriched regions were determined as potential centromere regions. For visualization, aligned and filtered BAM files were converted to normalized coverage files (bigwig) using Deeptools (v.3.5.5)^87^ with default parameters. The structure of tandem repeat sequences in each centromere region was confirmed using StainedGlass^88^ (v.0.6; window = 10,000).

### Pangenome analysis

Orthofinder^89^ (v.2.5.5) was used to construct the tetraploid wheat gene families, with -M msa -S blast -T iqtree. All gene families were classified into four categories: core (gene families present in all accessions), softcore (gene families present in 12 accessions), dispensable (gene families present in 2–11 accessions) and private (gene families present in one accession). To characterize the changes in the number of pan genes (dispensable and private genes) and core genes, we conducted 500 random samplings for each genome count scenario (ranging from 2 to 13) and plotted the variation curves using ggplot2^90^. The ratio of non-synonymous to synonymous substitutions (*K*_a_/*K*_s_) between each gene in the pangenome was estimated by the Nei–Gojobori approach^91^ built in the Bioperl Statistical module using barley as an outgroup based on sequence alignments performed with BLAST+ (v.2.16.0)^92^.

On the basis of the genome-annotation file, TE annotation results and pangenome data, the distribution characteristics of TEs in and around (±2 kb) all protein-coding genes in the pangenome were analyzed. For each gene, the transcriptional start/termination sites (TSS/TTS) were used as the centre to examine the 2-kb upstream and downstream regions. A sliding-window approach with adjustable window size (200 bp) and step size (20 bp) was used to calculate TE density. If a window extended beyond the chromosome boundaries, its TE density was marked as an invalid value. For the gene-body region, a fixed number of 100 windows was used. If the gene length was <2 kb, the number of valid windows was set to (gene length)/20, with the remaining windows marked as invalid. If the gene length was ≥2 kb, the window size was adjusted to (gene length)/100, generating 100 windows of TE density values. Finally, the average value for each window position was calculated across all genes, excluding invalid values from the computation.

### Phylogenetic tree construction and divergence time estimation

We constructed a phylogenetic tree of 13 wheat subspecies with barley cv. Morex v.3^93^ as the outgroup using single-copy gene families. Protein sequences of all single-copy genes (corresponding to the longest transcript) were aligned by MAFFT^94^ (v.7.526) and used to construct the phylogenetic trees by IQ-TREE2^95^ (v.2.3.5) with -AUTO. Phylogenetic trees were constructed using Astral^96^ (v.5.7.8) with -t 3.

Divergence time was estimated using MCMCtreeR^97^ (v.4.10.7), referenced in the divergence times of barley versus *T. turgidum* (10.3–13.6 Ma) from fossil records available at Timetree 5^98–101^ and wild emmer wheat versus cultivated tetraploid wheat (10,400 years ago). The phylogenetic tree of 13 tetraploid wheat and 34 hexaploid wheat accessions was constructed with barley as the outgroup. Sequence data for 34 hexaploid wheat accessions were downloaded from published studies^24,25,79,102–107^.

### *NLR* genes identification and classification

On the basis of the genome sequences of 13 tetraploid wheat and 34 hexaploid wheat accessions, the nucleotide-binding site leucine-rich repeat (NLR or NBS–LRR) sites were identified by NLR-Annotator^108^ (v.2.0). Subsequently, we compared the coordinates of identified NLR sites with the genome-annotation file in GFF3 format and genes with NLR sites were identified as *NLR* genes. In the case of two or more annotated transcripts for a gene, the longest transcript was prioritized for subsequent analysis. Furthermore, protein domains and integrated domains were predicted to divide the *NLR* genes into different classes using InterProScan^82^ (v.5.73; -dp -iprlookup). With the classification of *Arabidopsis* as reference^27^, *NLR* genes were classed into three classes based on the presence of coiled-coil, or resistance to powdery mildew 8 domains: coiled-coil domain-containing CNL-type (*CNL*) genes, *NL* genes (only including at least one NBS and LRR domain) and RESISTANCE TO POWDERY MILDEW 8 (RPW8)-domain-containing RNL-type (*RNL*) genes. Gene families (containing *NLR* genes) were filtered out to form the pangenome of *NLR* genes in tetraploid wheats. Further, compared to hexaploid wheats, the tetraploid gene families with *NLR* genes that could not correspond to the hexaploid genes were defined as specific NLR gene families of tetraploids. The distribution of the specific NLR gene families of tetraploids and hexaploids was plotted as a heatmap using the pheatmap R package (https://cran.r-project.org/web/packages/pheatmap/index.html).

### Subgenomic asymmetry analysis

GeneTribe^109^ (v.1.2.1) was used to identify syntenic homologous gene pairs between A and B subgenomes based on the longest transcript and protein sequence for each chromosome separately. On the basis of alignment results, GeneTribe^109^ (v.1.2.1) classified wheat gene relationships between A and B genes into three types: reciprocal best hit refers to two genes in a syntenic block being each other’s best match; singleton denotes genes with no homologous matches; single best hit represents a one-way best match in a syntenic block. Single best hit and singleton genes can reflect the biased genomic distribution of A and B subgenomes among tetraploid wheats and singleton genes showed the specific genes in subgenomes. The R package RIdeogram^110^ was used to map both asymmetric and symmetric genes on chromosomes. The functions of singleton genes were aligned to *O. sativa* L. proteins (v.IRGSP-1.0, Oryza_sativa - Ensembl Genomes 61) with BLAST+ (v.2.16.0)^92^ and the best hit (order of prioritization: high identity, high score, low *E*-value) was used to define the function of it.

### Graph-based pangenome construction

Using the Kronos genome as reference, the other 11 genomes were aligned using AnchorWave^62^ (v.1.2.6) with the proali command, -R 1 -Q 1. Alignment results were converted to VCF format using the MAFToGVCFPlugin of TASSEL^111^ (v.5.2.93) and all VCF files were merged by BCFtools^112^ (v.1.20). Then, all variants of VCF files were normalized using the left alignment algorithm implemented in vt^113^ (v.0.57721) tool. PanPop^114^ (v.NC2024) was used to filter the redundant variants and merge the similar multiallelic sites. From filtered results, we used the vg toolkit^115^ (v.1.56.0) for graph-based genome construction. IBS analyses were performed using PLINK^116^ (v.1.9) based on all SVs.

### Variant calling and filtering

Clean whole-genome sequencing reads for 621 accessions generated in this study and 115 from published data^8^ were mapped to the Kronos genome using BWA-MEM2 (v.2.3)^117^ with default parameters. SNPs and small indels (length <50 bp) were genotyped using the LUSH-DNASeq-pipeline^118^ (v.2.2.0). Only bi-allelic variants were retained and SNPs were filtered using QD < 2.0, SOR > 3.0, FS > 60.0, MQ < 40.0, MQRankSum < −12.5, ReadPosRankSum < −8.0. Indels were filtered using QD < 2.0, SOR > 10.0, FS > 200.0, MQRankSum < −12.5, ReadPosRankSum < −8.0.

To identify SVs in the 736 accessions, Illumina short reads were mapped to the tetraploid wheat graph-based pangenome using the vg giraffe (v.1.56.0)^119^ pipeline with default parameters. SVs were genotyped using Paragraph^120^ (v.2.3) with default parameters. VCF files were merged using BCFtools^112^ (v.1.20) and bi-allelic SVs were retained. SnpEff^121^ (v.5.2) was used for annotation and predicting the effects of identified SNPs and small indels. The variants with a missing rate >20% were discarded. Finally, all VCFs were imputed using Beagle^122^ (v.5.4) with default parameters.

### Phylogenetic tree and population structure analysis

First, variants were removed by linkage-disequilibrium-based pruning with a window size of 50 SNPs, window step of ten SNPs and *r*^2^ threshold of 0.2 using PLINK^116^ (v.1.9). Then, we selected 100,000 random variants for phylogeny construction. RAxML^123^ (v.8.2.12) was used to construct the maximum-likelihood tree with 100 bootstraps in the GTRGAMMA model. Phylogenetic trees were visualized and annotated using Interactive Tree of Life (iTOL) (https://itol.embl.de). Ancestry coefficient analysis was conducted using ADMIXTURE^124^ (v.1.3.0), initially with *K* ranging from 2 to 20, of which *K* = 10 was subsequently chosen. Linkage-disequilibrium decay distance was calculated using PopLDdecay^125^ (v.3.42). Principal component analysis was performed with GCTA^126^ (v.1.94.1).

### Genomic selective-sweeping analysis

Nucleotide diversity (*π*), population divergence (*F*_st_) and XPCLR scores were used to identify selective sweeps based on high-quality SNPs and small indels. For *π* and *F*_st_, VCFtools^127^ (v.0.1.16) was used with 500-kb sliding windows and 50-kb step size. XPCLR scores were calculated using XPCLR^128^ (v.1.1.2) with the same windows and step size. The R package GenWin^129^ was used to normalize and detect the boundary of selection regions. To identify selected SVs, we compared the allele frequency of each SV between wild emmer wheat and domesticated tetraploid wheat to identify selected SVs during domestication and between domesticated tetraploid wheat and free-threshing tetraploid wheat for selected SVs during improvement. The SV with an allele frequency ≥0.5 in at least one population and ≤0.5 at least one population simultaneously was used to determine its statistical significance. Fisher’s exact test was used to determine significance and the significance threshold for selected SVs was set as *P* < 0.05. If both the *P* value between wild emmer wheat versus domesticated emmer wheat and domesticated emmer wheat versus free-threshing was *<*0.05, the SV was identified as the selected SV during domestication and improvement continuously. Candidate genes were extracted using BEDTools^130^ (v.2.31.1).

### Genome-wide association studies

All high-quality SVs, SNPs and small indels with minor allele frequency (MAF) ≥ 0.05 were retained. All 373 accessions (WEW, DEW and DW) and 241 accessions (DEW and DW) were used for GWAS analysis using GEMMA^131^ (v.0.98.3) software with a mixed linear model, respectively. Kinship matrices were calculated using the command gemma -gk 2. The threshold for GWAS was determined by a uniform threshold of 0.05/*n* and 1/*n*, where *n* represents the number of input variants. Candidate quantitative trait loci (QTLs) and genes were identified through a multistep procedure^132,133^. First, within a 1-Mb region corresponding to the linkage-disequilibrium decay distance, the signal with the lowest *P* value was designated as the leading SNP, small indel or SV. Next, regions extending 1 Mb upstream and downstream of the leading variant, containing at least two statistically significant loci, were defined as candidate QTLs. Finally, genes located within the linkage-disequilibrium regions corresponding to these QTLs were considered as candidate genes.

### Heritability estimation

The proportion of phenotypic variance explained by genetic variants was estimated using LDAK^134^ (v.5.2) with the LDAK-thin model:

$$E\left[{h}_{j}^{2}\right]={\rm{tau}}_{i}{w}_{j}{[{f}_{j}(1-{f}_{i})]}^{(1+\rm{alpha})}$$

where *w*_*j*_ is the weighting for each variant, *f*_*j*_ is its MAF and tau are the corresponding coefficients and are estimated from the variant. All SNPs, indels and SVs were respectively first pruned to exclude nearby variants in perfect linkage disequilibrium using —window-prune 0.98 –window-kb 100. The best power parameter alpha was estimated for each trait by trying multiple values between −0.5 and 1. To filter out negative estimates, we added —constrain YES.

### Transgenic wheat plants construction

For heterologous expression, complementary DNA prepared from seedling leaves of the tetraploid wheat cultivars Kronos and Zavitan was used as template to amplify the open reading frames of *HAT14-B* and *TtGRAS*. These were then inserted into the pMWB122 vector to generate the *Ubi*_*pro*_*:HAT14* and *Ubi*_*pro*_*:TtGRAS* expression constructs driven by the *Ubiquitin* promoter from maize^135^. For RNAi lines, the 125-bp fragment with the approximately 95% highest *HAT14* A/B/D sequence similarity was inserted into the pWMB006 vector. The resulting DNA fragments were then inserted into the pCAMBIA3301 vector, generating RNAi constructs. All vectors carrying the desired constructs were introduced into *Agrobacterium tumefaciens* EHA105 and transformed into wheat cv. Fielder via *Agrobacterium*-mediated transformation^136^. Putative transformants were recovered on media containing phosphinothricin at 10 mg l^−1^, survivors were advanced to soil and transcript levels were quantified by quantitative reverse transcription PCR (qRT–PCR) (primers are listed in Supplementary Table 23). The *T*_2_ generation of transgenic lines was used for phenotyping. Phenotypic evaluation of transgenic lines was conducted (Supplementary Note 1).

### RNA isolation and qRT–PCR

For gene-expression assays, total RNA was isolated using the HiPure Plant RNA Mini Kit (R4151; Guangzhou Magen Biotechnology) and 2 μg of this RNA were reverse transcribed into cDNA using the HiScript II 1st Strand cDNA Synthesis Kit (R4151; Vazyme Biotech). The qRT–PCR was conducted with the ChamQ Universal SYBR qPCR Master Mix (Q711-02; Vazyme Biotech) in accordance with the instructions provided by the manufacturer. Relative expression levels were calculated on the basis of the 2^−ΔΔCt^ method^137^ and were normalized to *Actin1* (*TraesCS1A02G020500*). The experiments incorporated three technical replicates, with each replicate consisting of three plants. All primers used for qRT–PCR are listed in Supplementary Table 23.

### Subcellular localization

For the localization assays of *HAT14-B*^*Hap1*^ and *HAT14-B*^*Hap2*^, the pJIT163:GFP expression vector, containing the cauliflower mosaic virus (CaMV) *35S* promoter and harboring the single open reading frame of either *HAT14-B*^*Hap1*^ or *HAT14-B*^*Hap2*^, was introduced into wheat cv. Fielder mesophyll protoplasts through polyethylene glycol-mediated transformation^138^. The empty vector was transformed independently as a control. After incubation for 18 h at 23 °C in darkness, fluorescence was detected by a confocal microscope (FluoView FV3000, Olympus).

For GFP detection, the excitation laser wavelength was 488 nm with emission detected between 500 nm and 540 nm. For chlorophyll autofluorescence, excitation used a 640 nm laser and emission was detected from 650 nm to 750 nm. Laser intensity was controlled by adjusting the acousto-optical tunable filter to 1–4% and visualized with the image-analysis software supplied by the manufacturer.

### Dual-luciferase reporter assays

Dual-luciferase reporter assays were carried out^138^. The reporter construct was created by inserting approximately 2-kb promoters of *HAT14-B* and *TtGRAS* into the pGreenII 0800-LUC vector^139^. The effector constructs were generated by cloning the coding sequences of *HAT14-B*^*Hap1*^ into the pGreenII 62-SK vector^139^, which was driven by the CaMV *35S* promoter. *A. tumefaciens* GV3101 was transformed with each respective construct and recombinant strains were then used to co-infiltrate the leaves of 4-week-old *N. benthamiana* plants with the reporter and effector plasmids.

Luciferase levels were measured using a Dual-Luciferase Reporter Assay System (Promega) according to the manufacturer’s instructions. The normalized data are presented as the ratio of the luminescent signal intensity of the LUC reporter to that of the internal control REN reporter (*35S*_*pro*_*:REN*) from three independent biological samples.

### Electrophoretic mobility shift assay

The coding regions of *HAT14-B*^*Hap1*^ and *HAT14-B*^*Hap2*^ were cloned into pMAL-c5X to generate MBP–HAT14-B fusion vectors, respectively. The construct was validated by PCR and transferred into *Escherichia coli* BL21 (DE3) for recombinant protein expression. Biotin-labeled or unlabeled probes containing the AT-containing motif were synthesized by Sangon Biotech and unlabeled probes were used as competitors. Electrophoretic mobility shift assays were performed using the LightShift Chemiluminescent EMSA Kit (Thermo Fisher Scientific) following the manufacturer’s protocol. Primers are listed in Supplementary Table 23.

### Statistical analyses

Statistical details (for example, tests used, exact *n* values and significance thresholds) are reported in the figure legends. R (v.4.2.1) was applied for analysis unless otherwise indicated and any additional software used is documented in the relevant subsections.

### Reporting summary

Further information on research design is available in the Nature Portfolio Reporting Summary linked to this article.

## Online content

Any methods, additional references, Nature Portfolio reporting summaries, source data, extended data, supplementary information, acknowledgements, peer review information; details of author contributions and competing interests; and statements of data and code availability are available at 10.1038/s41588-026-02678-9.

## Supplementary information

Supplementary Information Supplementary Notes 1–5 and Figs. 1–34.

Reporting Summary

Peer Review File

Supplementary Tables Supplementary Tables 1–23.

Supplementary Data Statistical source data for Supplementary Figs. 14, 26, 27, 30 and 33.

## Source data

Source Data Fig. 2 Source data for Fig. 2g.

Source Data Fig. 3 Source data for Fig. 3e–g,i,j.

Source Data Fig. 5 Source data for Fig. 5k,l,n.

Source Data Fig. 6 Source data for Fig. 6e,h,i,k,m.

Source Data Fig. 5 Unmodified gels for Fig. 5g.

Source Data Fig. 6 Unmodified gels for Fig. 6g.

## Data Availability

Genome assemblies and HC gene annotations generated in this study have been archived in the Tetraploid Wheat Pan-Genome Database (http://omicsplant.cn/TetraploidWheatPangenomeDBs/) and are available via Zenodo at 10.5281/zenodo.18466336 (ref. ^140^). All raw sequencing reads generated from HiFi, ONT, Hi-C, RNA-seq, Iso-seq, ChIP–seq and whole-genome resequencing, as well as assembled genome sequences, have been deposited at the National Genomics Data Center under accession PRJCA043839 (GSA: CRA029005) (https://ngdc.cncb.ac.cn/bioproject/browse/PRJCA043839). Seed of 12 tetraploid wheat accessions assembled in this study are available from the corresponding author upon request for research purposes and their germplasm identifiers in gene bank are provided in Supplementary Table 1. Source data are provided with this paper.

## Code availability

Code associated with this project is available via GitHub at https://github.com/Lucio-Yang/Pan-genome and via figshare at 10.6084/m9.figshare.32213433 (ref. ^141^).
