# Supplementary material for: A pangenome of tetraploid wheat reveals the genetic architecture underlying domestication and genomic diversity for breeding
Source: Nat Genet. 2026 Jul 22;58(8):2027–39. doi: 10.1038/s41588-026-02678-9 (PMC13447107; doi:10.1038/s41588-026-02678-9)
Supplement: Supplementary file 1 [file 41588_2026_2678_MOESM10_ESM.pdf]

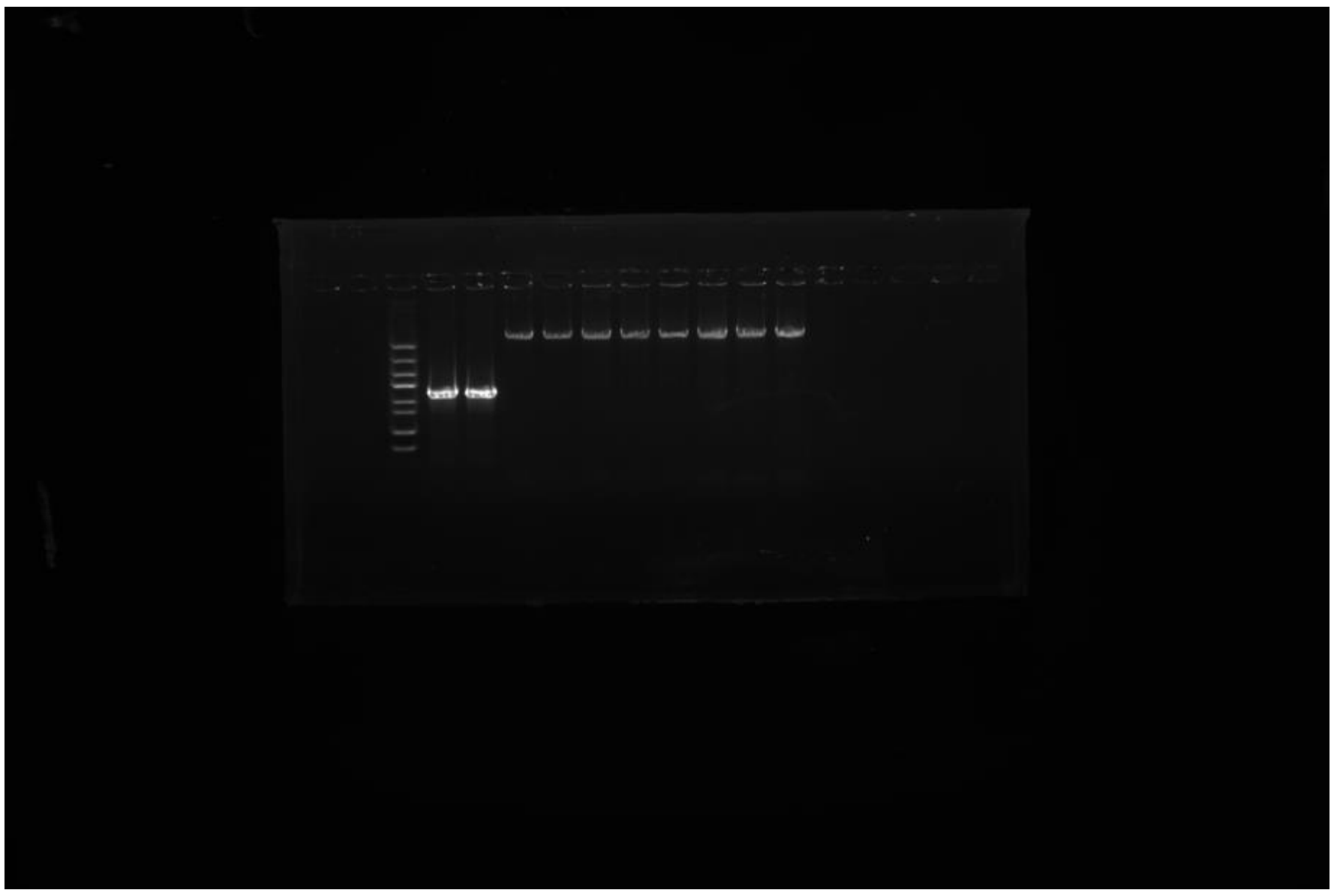

Fig 5g. Full-length unprocessed PCR gels verifying the 5,034-bp TE insertion within the *Btr1-A* coding region across eight Persian wheat accessions.
