# Supplementary material for: A pangenome of tetraploid wheat reveals the genetic architecture underlying domestication and genomic diversity for breeding
Source: Nat Genet. 2026 Jul 22;58(8):2027–39. doi: 10.1038/s41588-026-02678-9 (PMC13447107; doi:10.1038/s41588-026-02678-9)
Supplement: Supplementary file 2 [file 41588_2026_2678_MOESM11_ESM.pdf]

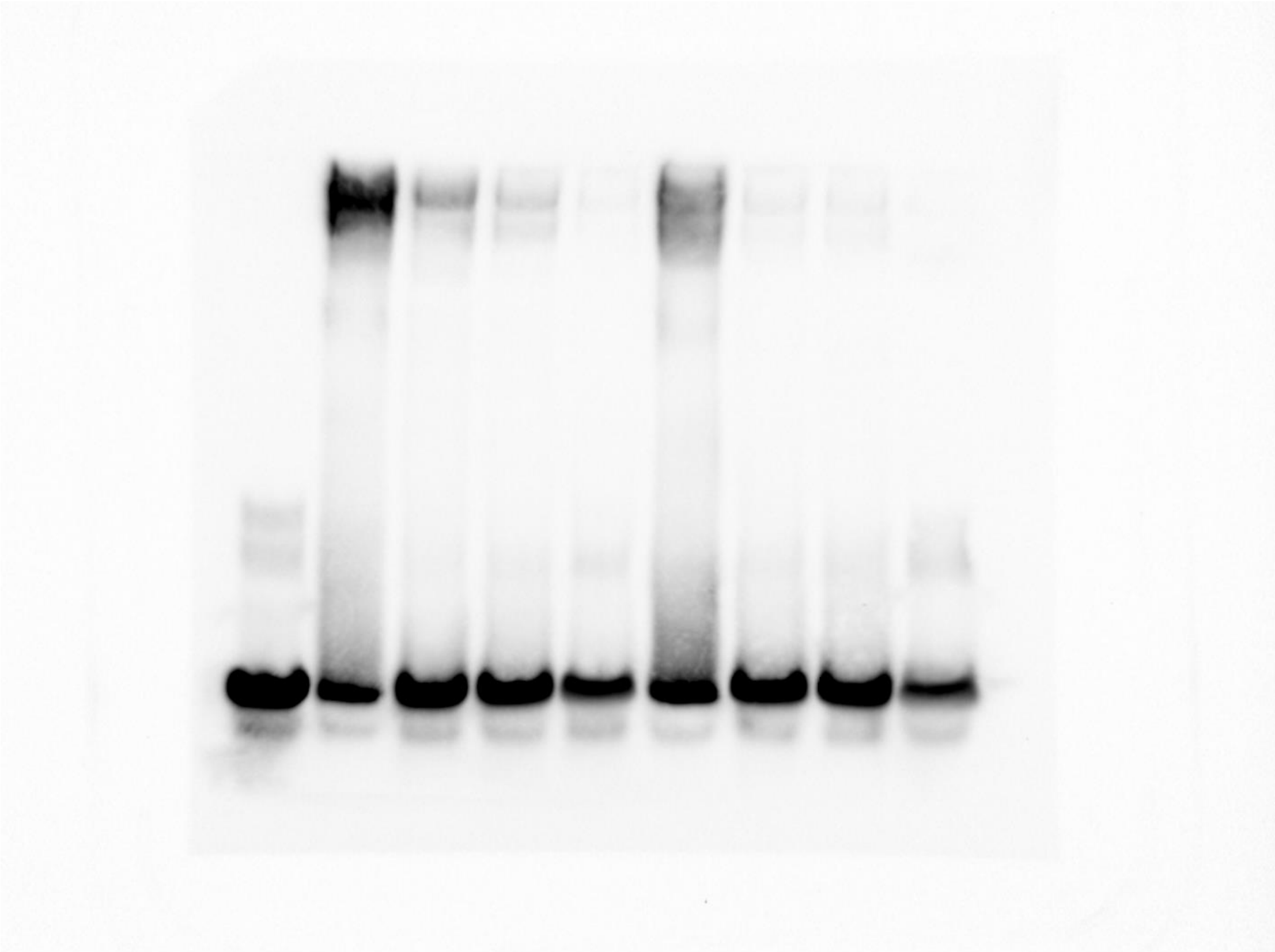

Fig 6g. Full-length unprocessed gel for the EMSA analysis.
