# Supplementary material for: A pangenome of tetraploid wheat reveals the genetic architecture underlying domestication and genomic diversity for breeding
Source: Nat Genet. 2026 Jul 22;58(8):2027–39. doi: 10.1038/s41588-026-02678-9 (PMC13447107; doi:10.1038/s41588-026-02678-9)
Supplement: Supplementary file 3 [file 41588_2026_2678_MOESM1_ESM.pdf]

nature genetics

Supplementary information https://doi.org/10.1038/s41588-026-02678-9

A pangenome of tetraploid wheat reveals the genetic architecture underlying domestication and genomic diversity for breeding

In the format provided by the authors and unedited

Supplementary Notes

Supplementary Note 1: Material and library preparation and sequencing.

Supplementary Note 2: Comparison between 12 genomes and population genetics analysis.

Supplementary Note 3: Cytological karyotyping methods and variation analysis.

Supplementary Note 4: Evolution network of tetraploid wheat.

Supplementary Note 5: Potential gene introgression among tetraploid wheats.

Supplementary Figures

Supplementary Fig. 1 Identified telomeres and assembly gaps on the *cv*. Kronos genome assembly.

Supplementary Fig. 2 Testing of sequencing-data size.

Supplementary Fig. 3. Chromatin-interaction map from Hi-C data for the 12 tetraploid wheat genome assemblies.

Supplementary Fig. 4 BUSCO assessments for the 12 genome assemblies.

Supplementary Fig. 5 Synteny dot plots between 12 assemblies and published tetraploid wheat genomes.

Supplementary Fig. 6 Comparison between 12 assembled genomes and published tetraploid wheat genomes.

Supplementary Fig. 7 Annotated centromere length for each chromosome for

the 12 assembled tetraploid wheat genomes.

Supplementary Fig. 8 Centromere structures for *cv.* Kronos.

Supplementary Fig. 9 Pan-genome analysis of 13 tetraploid wheat genomes.

Supplementary Fig. 10 Gene-ontology enrichment for core, softcore, dispensable and private genes.

Supplementary Fig. 11 Evolutionary tree among BBAA genomes of tetraploid and hexaploid bread wheats.

Supplementary Fig. 12 Gene-ontology enrichment for expanded and contracted gene families in key evolutionary nodes.

Supplementary Fig. 13 Pan-NLRomes of tetraploid wheat.

Supplementary Fig. 14 The A and B subgenomes of tetraploid wheat are asymmetrical.

Supplementary Fig. 15 Distribution of singleton genes on chromosomes 4A, 4B, 7A and 7B.

Supplementary Fig. 16 Summary of genome alignments for the 12 genomes.

Supplementary Fig. 17 FISH reveals three large karyotype variations in IG77365 and NU00021.

Supplementary Fig. 18 FISH identifies inversions on chromosome 5A in accession IG77365 and chromosome 7A in accession NU00021.

Supplementary Fig. 19 PCR verification of 30 randomly selected structural variants.

Supplementary Fig. 20 Geographic distribution and phylogenetic analysis of

736 tetraploid wheat accessions used in this study.

Supplementary Fig. 21 Analysis of gene flow from donor populations (P3) to recipient populations (P2) using Georgian wheat as the reference (P1).

Supplementary Fig. 22 Putative potential introgression (f_d) and nucleotide diversity (π) across each chromosome.

Supplementary Fig. 23 The relationship between introgression (f_d) and nucleotide diversity (π) among free-threshing tetraploid wheats.

Supplementary Fig. 24 Nucleotide diversity of wild emmer wheat, domesticated tetraploid wheat and free-threshing tetraploid wheat.

Supplementary Fig. 25 Linkage-disequilibrium-decay analysis for different types of genomic variation.

Supplementary Fig. 26 Phenotypes of tetraploid wheat individuals with two *TtGRAS-A* haplotypes.

Supplementary Fig. 27 Phenotype (a) and statistical analysis (b) of effective tiller number in *TtGRAS-A* overexpression lines.

Supplementary Fig. 28 Heritability of 32 traits contributed by genomic structural variants (SVs), SNPs and indels.

Supplementary Fig. 29 Chromosomal distribution of QTLs related to grain, plant-architecture and abiotic-stress traits.

Supplementary Fig. 30. Genome-wide association study for grain length/width and drought tolerance.

Supplementary Fig. 31 Phylogenetic analysis of HAT14-B homologs.

Supplementary Fig. 32 Haplotype analysis for *HAT14-B* on chromosome 1B.

Supplementary Fig. 33 Phenotype (a) and statistical analysis (b) of effective tiller number in *HAT14-B*^{Hap1} overexpression and RNAi lines.

Supplementary Fig. 34 Full-length, unprocessed gel corresponding to Supplementary Fig. 19.

Supplementary Note 1: Material and library preparation and sequencing

Planting

All accessions were planted at the experimental station of the Peking University Institute of Advanced Agricultural Sciences (Weifang, China) in 2022–2024 for sampling sequencing materials. To investigate plant architecture and grain traits, we also planted those accessions as well as transgenic lines at the experimental station of Luoyang Academy of Agricultural and Forestry Sciences (Luoyang, China) and Northwest A&F University (Yangling, China) in the 2023–2024 growing seasons.

HiFi library preparation and sequencing

Young leaves at the three-leaf stage were collected and genomic DNA (gDNA) was extracted using CTAB (Solarbio, LS00066). DNA quality was analysed using NanoDrop (Thermo Fisher Scientific, ND2000), Qubit (Invitrogen, Q33238), and CHEF Mapper XA Chiller System (Biorad, 1703672). After examination of the quality of isolated DNA, a library of 15 kb was constructed using a SMRTbell prep kit 3.0 (Pacific Biosciences, 102-182-700). Library size selection was conducted by Pippin HT (Sage Science). After quality-control testing using the Agilent Fragment Analyzer System (Agilent Technologies, M5310AA), SMRTbell libraries were obtained. All libraries were sequenced on the PacBio Revio system (Pacific Biosciences, 102-090-600) by Berry Genomics Corporation (Beijing, China).

Hi-C library preparation and sequencing

Hi-C sequencing[1] data were generated in five steps: cells of young leaves were crosslinked with formaldehyde; enzymatic cleavage of DNA with the restriction enzyme *Dpn*II; DNA ends were filled and marked with biotin; DNA after terminal

repair was cyclized; DNA was purified and randomly sheared into 300–500-bp fragments. Sequencing of Hi-C libraries was performed on the NovaSeq™ XPlus platform by BioMarker Corporation (Beijing, China).

Nanopore ultra-long library construction and sequencing

For Oxford Nanopore sequencing, DNA extraction, library preparation and sequencing were conducted[1]. In brief, high-quality genomic DNA was isolated from fresh leaves using CTAB, and DNA quality and concentration were examined by 0.75% agarose gel electrophoresis, NanoDrop One spectrophotometer (Thermo Fisher Scientific, USA) and Qubit 3.0 Fluorometer (Life Technologies, USA). Libraries were prepared using the SQK-LSK114 ligation kit according to the manufacturer's instructions. The purified library was loaded onto primed R10.4.1 Spot-On Flow Cells and sequenced using a PromethION sequencer (Oxford Nanopore Technologies, UK) with 48–72-h runs carried out at Wuhan Benagen Technology Co., Ltd. (Wuhan, China).

Re-sequencing library preparation and sequencing

Genomic DNA of 622 accessions (including Kronos) was extracted using CTAB and used to construct sequencing libraries using VAHTS®Universal Plus DNA Library Prep Kit for IIlumina ND617 according to the manufacturer's directions. Each accession was sequenced on DNBSEQ-T7 sequencing platform using 150-bp paired-end reads. The target sequencing depth was 15-fold for each accession.

ChIP-seq library preparation and sequencing

The peptide of the specific N-terminal tail of TaCENH3, 'ARTKHPAVRKTK', was

expressed in the pET28b vector fused to a His-tag in *Escherichia coli* BL-21. An anti-rabbit polyclonal antibody against this peptide was prepared and purified using rabbit serum by AtaGenix (Wuhan, China). ChIP assays were performed[2,3], cells, isolated from 7-d-old seedlings, were harvested and crosslinked with 1% (v/v) formaldehyde for 10 min at room temperature. After sonication, the immunoprecipitation was incubated overnight at 4°C with 6 μg of antibody to enrich DNA. Antibodies were captured using Dynabeads Protein G, and cross-links were reversed overnight at 65°C. DNA was extracted and purified using the MinElute PCR Purification Kit (QIAGEN, 28006). ChIP-seq libraries were prepared using TruePrep DNA Library Prep Kit V2 for Illumina (Vazyme, TD501) according to the manufacturer's instructions. For ChIP-seq, extracted DNA was ligated to specific adaptors, followed by deep sequencing in the Illumina Novaseq 6000 using 150-bp paired ends.

RNA-seq library preparation and sequencing

Total RNA was extracted according to the instructions of the RNAprep Pure Plant Kit (TIANGEN, DP441). The integrity, quality and concentration of RNA were detected by NanoDrop (Thermo Fisher Scientific, ND2000) and Agilent 2100 Bioanalyzer instrument (Agilent Technologies, G2939BA). Strand-specific dUTP RNA-seq libraries were constructed for roots, leaves and stems at the three-leaf stage. The resulting libraries were sequenced in the 150-bp paired-end mode using the NovaSeq™ XPlus system at BioMarker (Beijing, China), which produced around 10 Gb of data for each tissue in each sample. For PacBio Iso-seq, equal concentrations of RNA from nine separate tissues of each accession were mixed, including stems, flag leaves, internodes below the spikes, awns, spikes and grains at 14-d post-heading stage; whole seedlings (three-leaf stage) grown in 1/2 Hoagland nutrient

solution, and whole seedlings (three-leaf stage) treated with 250 mM NaCl and 20% w/v PEG-6000. Library preparation was performed using the Iso-Seq express 2.0 kit (Pacific Biosciences, 103-071-500) and the Kinnex full-length RNA kit (Pacific Biosciences, 103-072-000). All libraries were sequenced on the PacBio Revio system (Pacific Biosciences, 102-090-600) by Berry Genomics (Beijing, China).

Quality control

Revio sequencing data were subjected to quality control using SMRT Link (https://www.pacb.com/support/software%20downloads/, v13.1). This process filtered out low-quality reads with an average read quality value of ≤ 0.99, as well as adapter sequences. ONT sequencing data were generated in the pod5 format, and dorado (https://github.com/nanoporetech/dorado, v0.4.1) was used in "sup" mode for base calling. We used NanoPack[4] (v1.1.1) for ONT read processing and only retained reads longer than 100 kb and a quality value ≥ 10. Illumina and BGI sequencing data were quality controlled using Fastp[5] (v0.23.2) by removing adapter sequences and low-quality sequences and retaining the final set of high-quality reads for subsequent analysis. Finally, we obtained 998.37 Gb HiFi, 1,035.09 Gb Hi-C and 576.75 Gb ONT data for the Kronos accession assembly. For the other 11 *de novo* assembly accessions, an average of 556.46 Gb of cleaned HiFi and 439.39 Gb of cleaned Hi-C data were obtained. For all 622 accessions, an average of 153.68 Gb of whole-genome sequencing cleaned data per sample was obtained (Supplementary Table 12).

Genome size assessment

To estimate genome size, we used Kmerfreq (https://github.com/fanagislab/kmerfreq)

to count *k*-mer frequency based on HiFi reads. Genome-size estimation was performed using GCE[6] (v1.0.2). Genomescope[7] (v1.0) and findGSE[8] (v0.1.0) were used for visualization.

Testing of HiFi and Hi-C data at different depths

To determine the sequencing saturation of HiFi and Hi-C for tetraploid wheat, we generated HiFi data (998 Gb total; 96× coverage) and Hi-C data (1,035 Gb total; 99.5× coverage) for *cv.* Kronos. Contig assemblies were generated from subsampled HiFi datasets using hifiasm[9] (v0.22.0-r689). Assembly quality and completeness were evaluated using contig N50 and BUSCO integrity metrics (Supplementary Fig. 2), with BUSCO[10] (v5.4.6) and the Poales lineage dataset (poales_odb10; OrthoDB, 2020-09-10).

All Hi-C data were partitioned into two subsets: 400 Gb (~40× coverage) and 600 Gb (~60× coverage). All reads and reads from each subset were processed using Arima Genomics' mapping pipeline (https://github.com/ArimaGenomics/mapping_pipeline, v03) to perform single-end read mapping, filter chimeric reads and PCR duplicates, and generate valid read pairs. Resulting Hi-C contact maps were then scaffolded into chromosome-scale assemblies using YaHS[11] (v1.2a.1, with parameters --no-contig-ec --no-scaffold-ec -e GATC). Finally, scaffolds were manually curated in Juicebox[12] (v1.11.08) based on Hi-C interaction patterns and telomere positional information.

AnchorWave[13] (v1.2.6) was applied to compare the scaffolding results with the published genome of Svevo[14]. The scaffolding results obtained using Hi-C sequencing data of different depths (~40×, ~60×, ~100×) showed minimal differences. It was demonstrated that 40× Hi-C sequencing data were adequate to scaffold contigs to the chromosome level (Supplementary Fig. 2).

To visually inspect the assembly using a Hi-C heatmap, we used HiC-Pro[15] (v3.1.0) to filter out invalid Hi-C read pairs. The Matrix2heatmap.py script (https://github.com/fanagislab/EndHiC/blob/master/matrix2heatmap.py, with a parameter that compresses every 10 bins into one) was used to generate Hi-C interaction heatmaps from HiC-Pro results (hic_100000.matrix) for Hi-C data subsampled to different sequencing depths. In the final heatmap, each bin represents a 1-Mb genomic region, with color intensity proportional to the $\log_2$-transformed Hi-C interaction frequency between or within these 1-Mb bins.

Phenotypic evaluation

For phenotypic evaluation, a panel of 373 representative accessions was grown in the experimental farms of Yangling, Shaanxi (Northwest A&F University, 107.97996° E, 34.29109° N) and Luoyang, Henan, China (Luoyang Academy of Agricultural and Forestry Sciences, 112.48649° E, 34.63484° N) in the October 2023 to June 2024 growing season. The field trials were performed with a randomized block design and each accession was planted in a three-row plot with a row length of 1.5 m, with 30 seeds per row, at a row spacing of 0.25 m.

Agronomic traits were measured at physiological maturity before harvest in the field. For each accession, at least 30 plants were randomly selected to investigate plant height, spike length, spikelet number, flag-leaf length, flag-leaf width and awn length. Subsequently, the main spikes of 30 plants were manually harvested and threshed. Grain-related traits, including thousand-kernel weight, grain width, grain length, grain area, grain diameter, grain circumference, grain roundness and the ratio of grain length to width, were measured using a grain analysis system (SC-E, Wseen, China).

To investigate drought- and salt-related traits, all seeds were cultivated in a greenhouse at Northwest A&F University (Yangling, China) under a 16-h light and 8-h dark cycle, with day and night temperatures of 16°C and 14°C, and a relative humidity of 40%. Intact seeds were selected to soak in 10% v/v sodium hypochlorite solution for 10 min, followed by three or four washes with fresh water. Seeds were divided into three groups and grown in Hoagland solution for one week. One group had 250 mM NaCl added as the salt-stress treatment and the other had 20% w/v PEG-6000 added as the simulated drought-stress treatment, while the remaining group was used as a control. After 10 d of treatment, phenotypic traits were scored using five biological replicates, including above- and below-ground biomass (fresh weight and dry weight), water content ((fresh weight – dry weight)/fresh weight), plant height, and longest root length. The drought-tolerance index (DTI) and salt-tolerance index (STI) were calculated as the ratio of the mean trait value under stress conditions to the mean trait value under normal control conditions[16]. Statistical analysis was performed using R (4.2.1, https://www.r-project.org/).

Supplementary Note 2: Comparison between 12 genomes and population genetics analysis

Genome assembly assessment

Two tetraploid wheat genomes have been reported, including one wild emmer genome (Zavitan)[17] and one durum genome (Svevo)[14]. The assemblies *de novo* of the Zavitan and Svevo genomes mainly used Illumina short-read sequencing data, with contig N50 values of 57,378 bp and 56,196 bp, respectively. The scaffold N50 is 6.96 Mb for Zavitan and 5.97 Mb for Svevo. Based on optical maps, the Zavitan genome assembly was further improved in 2019[18], of which the 2,660 scaffolds were

re-anchored and scaffold N50 increased to 65.67 Mb. Taking advantage of progress in long-read sequencing and genome-assembly technology, the contig N50 of the recently released genome assembly of *cv.* Langdon[19] reached 41.18 Mb, and its scaffold N50 was 751.30 Mb.

Here, we assembled *de novo* 12 tetraploid wheat genomes, achieving reference quality as suggested by the LAI parameter. The contig N50 length of the genome assembled is 54 Mb on average, and the scaffold length achieved 756.21 Mb on average (Supplementary. 6a). The effective chromosome length (genome sequence without 'N') of Zavitan v3 was longer than that of Zavitan v2 and v1 (Supplementary Fig. 6b). These results demonstrate that this study has assembled high-quality, reference-level genomes.

Detecting expansions and contractions of gene families

Gene families were identified using OrthoFinder[20] (v2.5.5) with parameters '-M msa -S blastp'. Using gene-family counts, we examined expansions and contractions of gene families at each node of the evolutionary history of the studied wheat genomes using CAFE[21] (v5). The results were shown by the ChiPlot website (TVBOT).

Gene-flow identification

The D statistic[22] can be used to reflect introgression proportions at the genome-wide level, and the f_d statistic[23] can estimate introgression proportions within a specific size window. To explore the introgression regions among the tetraploid wheat accessions, we calculated D and f_d values across the whole genome using Python code from https://github.com/simonhmartin/genomics_general, with the window size = 100 and step size = 50 SNPs. The minimum number of loci in each window was

set to 3. The f_d statistic ranges from 0 to 1, and the D statistic ranges from –1 to 1. When windows with D < 0, or D > 0, but f_d > 1, the f_d statistic was biologically meaningless or noisy; therefore, f_d was set to zero. Using barley and Georgian wheat as the outgroup and P1, we estimated the f_d statistic using different population combinations, containing gene flow from wild emmer wheat to the other two domesticated tetraploid wheats and six free-threshing tetraploid wheats; gene flow from domesticated tetraploid wheats to all free-threshing tetraploid wheats; gene flow between domesticated emmer wheat and Ispahanicum; and gene flow across all free-threshing tetraploid wheats. We also used the proportion of genome introgression (PGI) value, defined as the overall proportion of introgression across the whole genome[24]. To discover the relationship between gene flow and nucleotide diversity (π) in tetraploid wheat populations, we analysed nucleotide diversity and f_d in each window. Boxplots of the f_d statistics for each bin were plotted using the matplotlib function from the Pyplot module[25].

Enrichment analysis

Annotation files of gene-ontology (GO) terms were produced using Interproscan[26] (v5.73), and the enrichment analysis was performed using a custom Python script (https://github.com/Lucio-Yang/Pan-genome/tree/main/03.Pan-genome_analysis/scripts/GO_enrichment). Enrichment significance was analysed using the Hypergeometric test, and a *P*-value of < 0.05 was considered statistically significant. Statistically significant GO terms were plotted with the R package ggplot2.

Supplementary Note 3: Cytological karyotyping methods and variation analysis

Chromosome spreads were prepared following conventional cytological preparation methods[27]. Non-denaturing fluorescence *in situ* hybridization (ND-FISH) of five repetitive-sequence probes, Oligo-pSc119.2-1 (5′-CCGTT TTGTG GACTA TTACT CACCG CTTTG GGGTC CCATA GCTAT-3′), Oligo-pTa535-1 (5′-AAAAA CTTGA CGCAC GTCAC GTACA AATTG GACAA ACTCT TTCGG AGTAT CAGGG TTTC-3′), Oligo-5SrDNA (5′-GTACT ACTCT CGCCC AAGCA CGCTT AACTT CGGAG TTCTG A-3′), Oligo-3A1 (5′-AATAA TTTTA CACTA GAGTT GAACT AGCTC TATAA GCTAG TTCA-3′), Oligo-CCS1 (5′-CCGTT TGATA GAGGC AAAGG TGTCC CGTCT TTTGA TGAGA-3′) and Oligo-v03-86 (5′-CGAGG CGGAC GTCGT CGCGG TAGCG ACGAC GGACG CCGAG ACGAG CACGT-3′) was performed[28–31]. Chromosomes were counterstained with DAPI (4′,6- diamidino-2-phenylindole) and chromosome images were captured with an Olympus BX-53 microscope and a DP70 CCD camera.

For karyotyping, the target accession and control accessions were examined, and their karyotypes were compared to that of Chinese Spring[28–30,32]. We also confirmed karyotype variation using Hi-C heatmaps. In brief, cleaned Hi-C reads for the target accession were mapped to the target accession genome and control accessions genomes using BWA-MEM2[33] (v2.3). Raw Hi-C link counts were calculated in 1-Mb non-overlapping sliding windows and then normalized. Finally, the normalized Hi-C contact matrix was visualized using R.

Mitotic metaphase chromosomes were prepared by conventional acetocarmine-squash and non-denaturing fluorescence *in situ* hybridization (ND-FISH) using five repetitive-sequence probes was performed: Oligo-pSc119.2-1[28], Oligo-pTa535-1[28], Oligo-5SrDNA[31], Oligo-3A1[30], Oligo-CCS1[31], Oligo-v03-86[28].

Oligo-3A1 and Oligo-5SrDNA were used to confirm chromosomes 5A and 7A. The major karyotypic difference to the Kronos genome was the translocation between chromosomes 2A and 4B of accession IG77365, and chromosomes 6A of accession NU00021. The simultaneous translocation (between chromosome 2A and 4B) and inversion (chromosome 5A) variation was presented by shifting Oligo-pSc119.2-1 and Oligo-pTa535 and inverting Oligo-CCS1 and Oligo-v03-86 in IG77365. The inversion on chromosome 7A near the centromere was presented by inverting Oligo-CCS1 and Oligo-v03-86 in NU00021.

Supplementary Note 4: Evolution network of tetraploid wheat

The network-based approach used for bread wheat was used to reconstruct the species history and community structure of tetraploid wheat. To further understand the evolutionary history of tetraploid wheat subspecies, we employed a phylogenetic network approach. Sequenced accessions were categorized into three groups: wild emmer wheat, domesticated tetraploid wheat, and free-threshing tetraploid wheat. Based on a high-quality VCF file (see *Variant calling and filtering* section for details), we divided the genome into 1,000 parts and performed repeated random-haplotype sampling at heterozygous sites, randomly sampling 2,000 SNP sites each time to infer 1,000 maximum-likelihood tree topologies with the GTRGAMMA model and JC69 distances in RAxML[34] (v8.2.12) (asc-corr = felsenstein). While these repeated random haplotype sampling procedures including heterozygous sites (RRHS) trees were also analysed in the form of conventional consensus topologies and Densitree visualizations to infer taxonomic clades, we used the Kruskal algorithm[35] implemented in Python (https://github.com/Lucio-Yang/Pan-genome/) to analyse the evolutionary distances between each pair of accessions using 1,000 minimum

spanning trees (MST). MST graphs were subsequently combined into a weighted, phylogenetic consensus network whose nodes were clustered into clades using the Girvan–Newman EdgeBetweenness algorithm[36] implemented in the CytoCluster plugin[37] (v2.1.0) of Cytoscape[38] (v3.6). The clustered network topology was plotted considering edge-betweenness in Cytoscape and taxonomic clades were inferred by the intersection of community clusters with taxon information that was annotated using the AutoAnnotate plugin[39] (v1.5.2). The relative number of RRHS trees for which a respective edge was selected by the MST algorithm was used as edge weights and interpreted as bootstrap-support values in the consensus tree topologies. The network uncovered extensive reticulation events, indicating pervasive gene flow across lineages. We observed fewer connections between the wild tetraploid wheat and the free-threshing tetraploid (number of edges = 9,503) compared to between the domesticated tetraploid wheat and the free-threshing tetraploid (number of edges = 31,076) (Fig. 4f). Further classifying all the individuals into 10 subspecies uncovered limited connections between durum from Persian (*T. carthlicum*, number of edges = 3,626) (Fig. 4g). Very rare putative gene flows were observed from Ispahanicum (*T. ispahanicum*, number of edges = 252) and Georgian (*T. karamyschevi*, number of edges = 618) tetraploid wheat to free-threshing tetraploid wheats, highlighting untapped genetic resources for breeding. More edges were observed between the free-threshing subspecies and the domesticated emmer wheat (number of edges = 22,042) than between the free-threshing subspecies and wild emmer (number of edges = 7,988), further supporting the hypothesis of the two-step evolutionary model: from wild tetraploid wheat to domesticated tetraploid and from domesticated tetraploid to free-threshing tetraploid.

Supplementary Note 5: Potential gene introgression among tetraploid wheats

Introgression of genomic fragments from diploid and tetraploid wheat is a primary genetic resource for enhancing genetic diversity and environmental adaptability in bread wheat[24,40,41]. However, knowledge of inter-population introgression in tetraploid wheat is lacking. We used the f_d statistical approach to quantify potential introgression in 100-SNP windows. Meanwhile, we also inferred PGI[24] to reflect the size of genome fragments derived from potential alien introgression. As Georgian wheat was genetically distant from the other tetraploid wheat subspecies based on the evolution network and its nucleotide diversity was lower (Fig. 4e and g), we used Georgian wheat as the reference population (P1).

The inferred introgression segments from wild emmer wheat to the free-threshing tetraploid wheats (average f_d = 0.09; average PGI = 0.17) showed statistically significantly lower f_d values and PGI values than those from domesticated tetraploid wheats (average f_d = 0.15; average PGI = 0.28; one-tailed t-test, P-value < 0.001; Supplementary Fig. 21; Supplementary Table 13). Within free-threshing tetraploid wheats, excepting for Persian wheat, the potential introgression ratio was relatively higher, indicating pervasive gene flow. Consistent with a published study[24], we also identified several potentially introgressed segments spanning hundreds of megabases near pericentromeric regions (Supplementary Fig. 22) on the majority of chromosomes. The largest potential introgressed segment was located on chromosome 1A (82–260 Mb) between wild emmer wheat and domesticated tetraploid wheat, covering approximately 178 Mb/39.97% of the whole chromosome.

In the tetraploid wheats, introgression correlated with nucleotide diversity (π) in a non-monotonic pattern. Genomic regions with high and low proportions of introgression fragments displayed lower diversity than regions with intermediate

proportions (Supplementary Fig. 23a). For example, a 2.21-fold and 1.34-fold increase in π was observed in intermediate-proportion introgression regions of Khorasan wheat (*T. turanicum,* 0.4 > f_d > 0.3, π = 0.002091) compared to low-proportion introgression regions (0.1 > f_d > 0, π = 0.000948) and high-proportion introgression regions (0.7 > f_d > 0.6, π = 0.001559). Functional annotations linked introgressed segments to adaptive genes (e.g., grain-protein regulator *NAM-B1* and grain-size gene *GW2-B*), suggesting that alien alleles provide potentially beneficial natural alleles for agronomic traits (Supplementary Fig. 23).

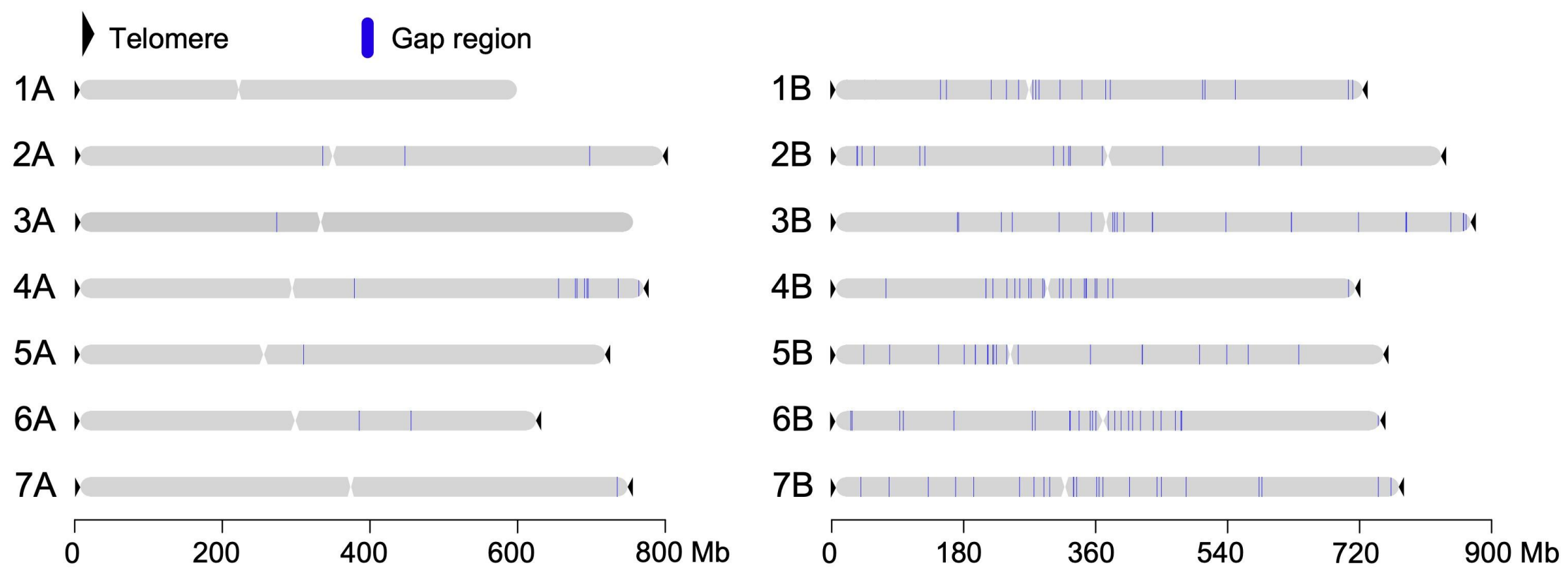

Supplementary Fig. 1 Identified telomeres and assembly gaps on the *cv.* Kronos genome assembly.

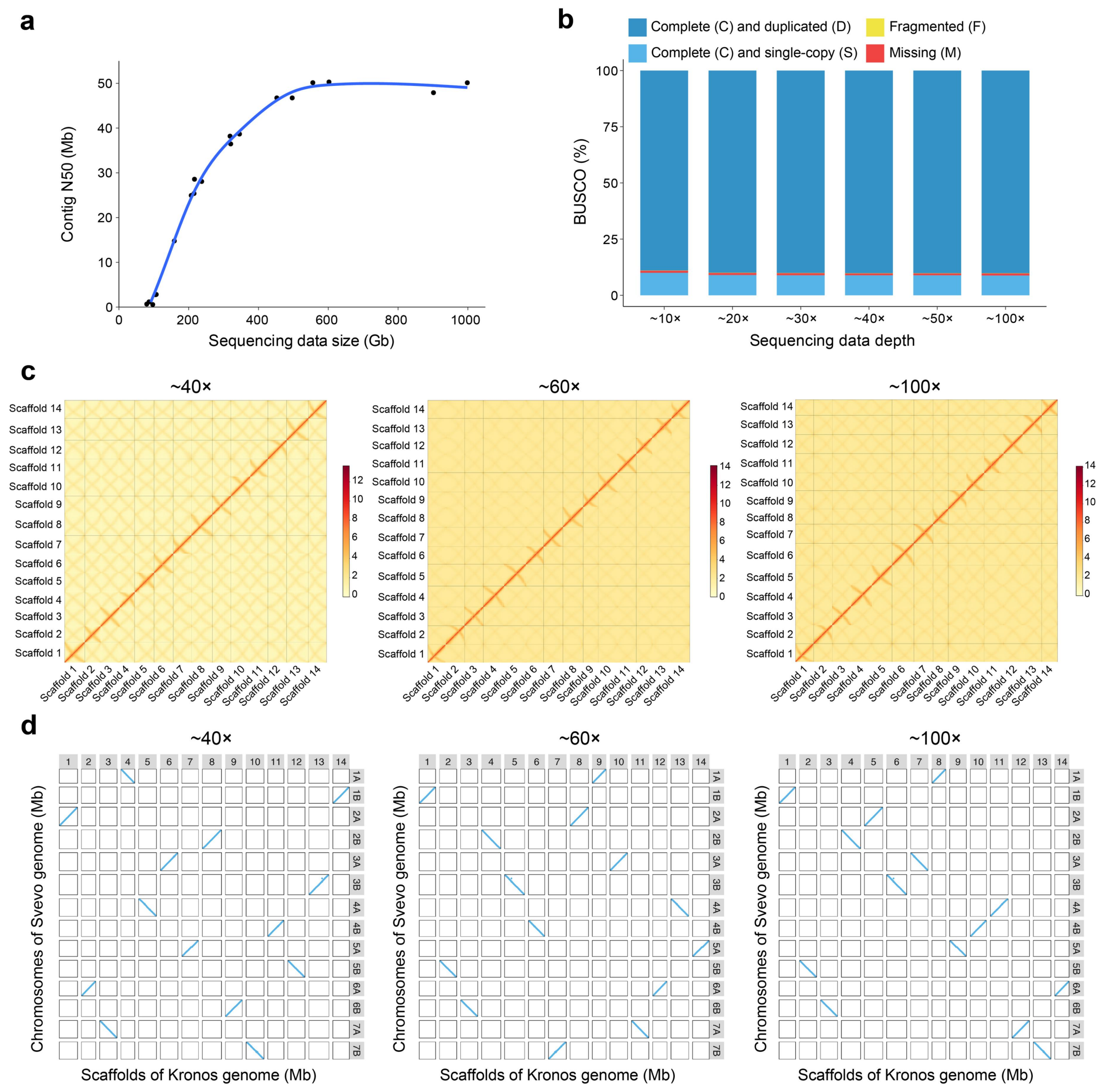

Supplementary Fig. 2 Testing of sequencing-data size. a–b Contig N50 length (**a**) and BUSCO assessment (**b**) of the *cv.* Kronos genome assembled in this study from different HiFi sequencing-depth data. **c–d,** Scaffolding (**c**) and synteny (**d**) results of genomes assembled with the assistance of different Hi-C data sizes.

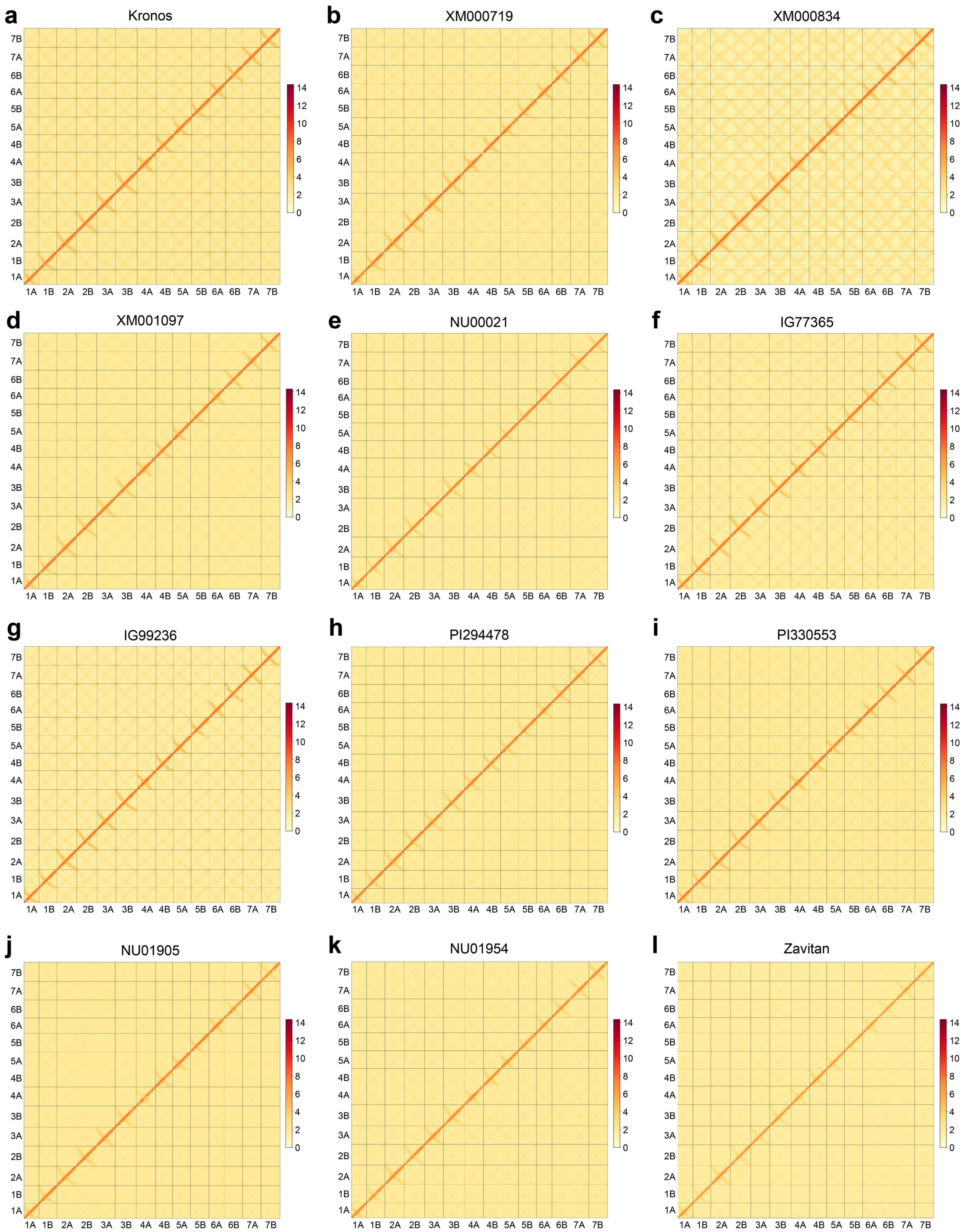

Supplementary Fig. 3 Chromatin-interaction map from Hi-C data for the 12 tetraploid wheat genome assemblies. Interactions were computed at a 1-Mb resolution.

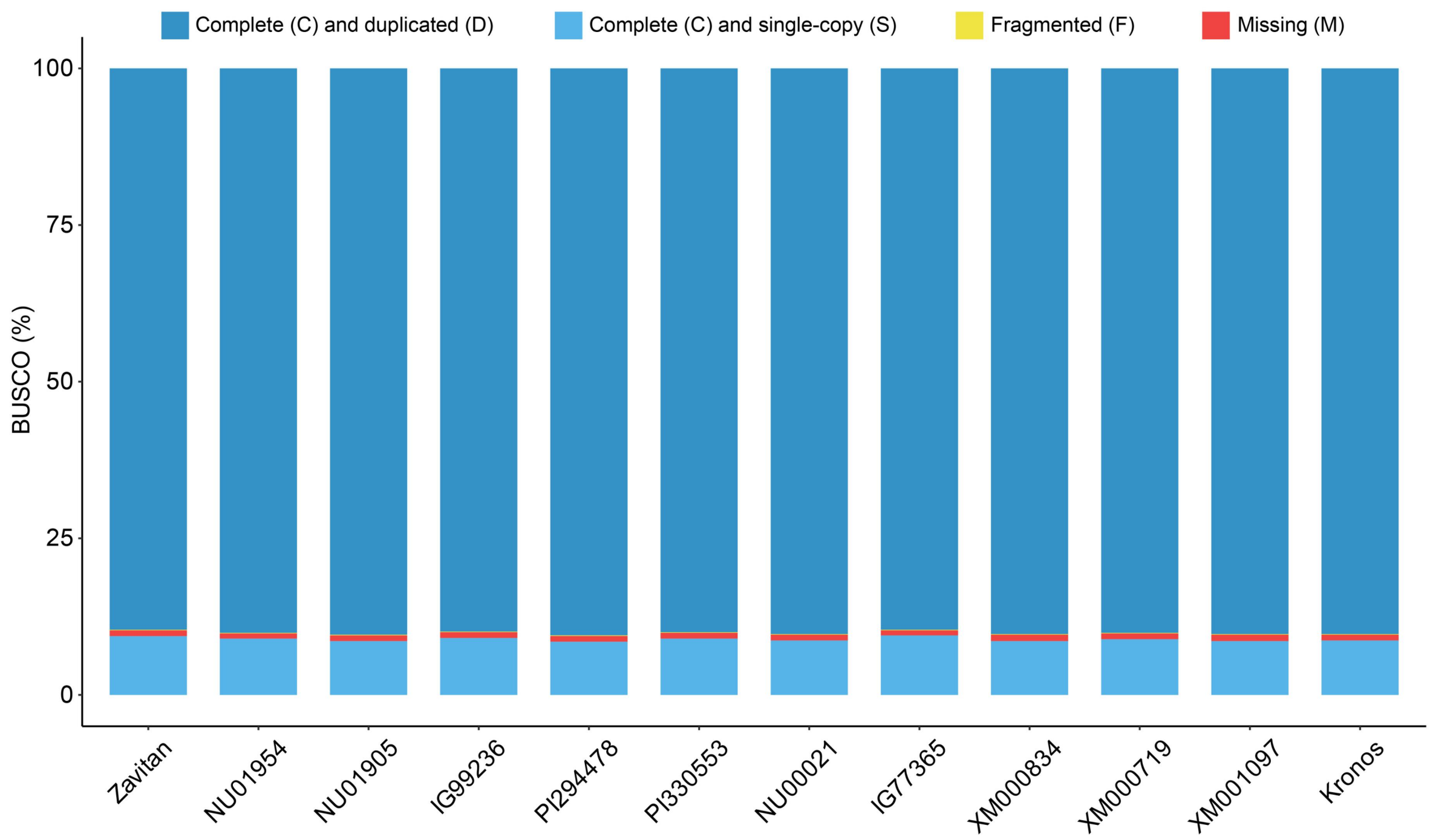

Supplementary Fig. 4 BUSCO assessments for the 12 genome assemblies.

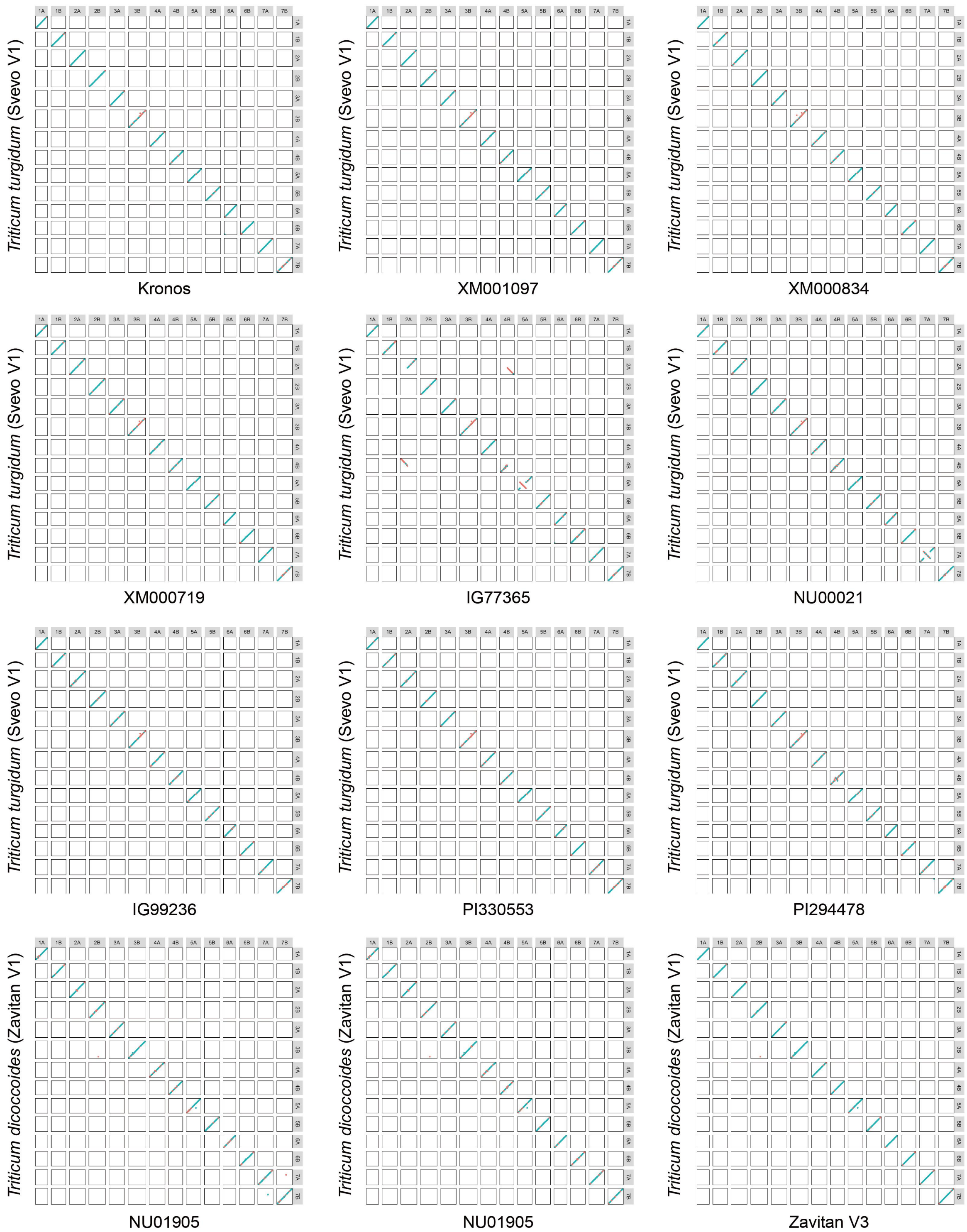

Supplementary Fig. 5 Synteny dot plots between 12 assemblies and published tetraploid wheat genomes. Turquoise dots represent the sequences from two assemblies on the same strand, and red represents sequences on different strands.

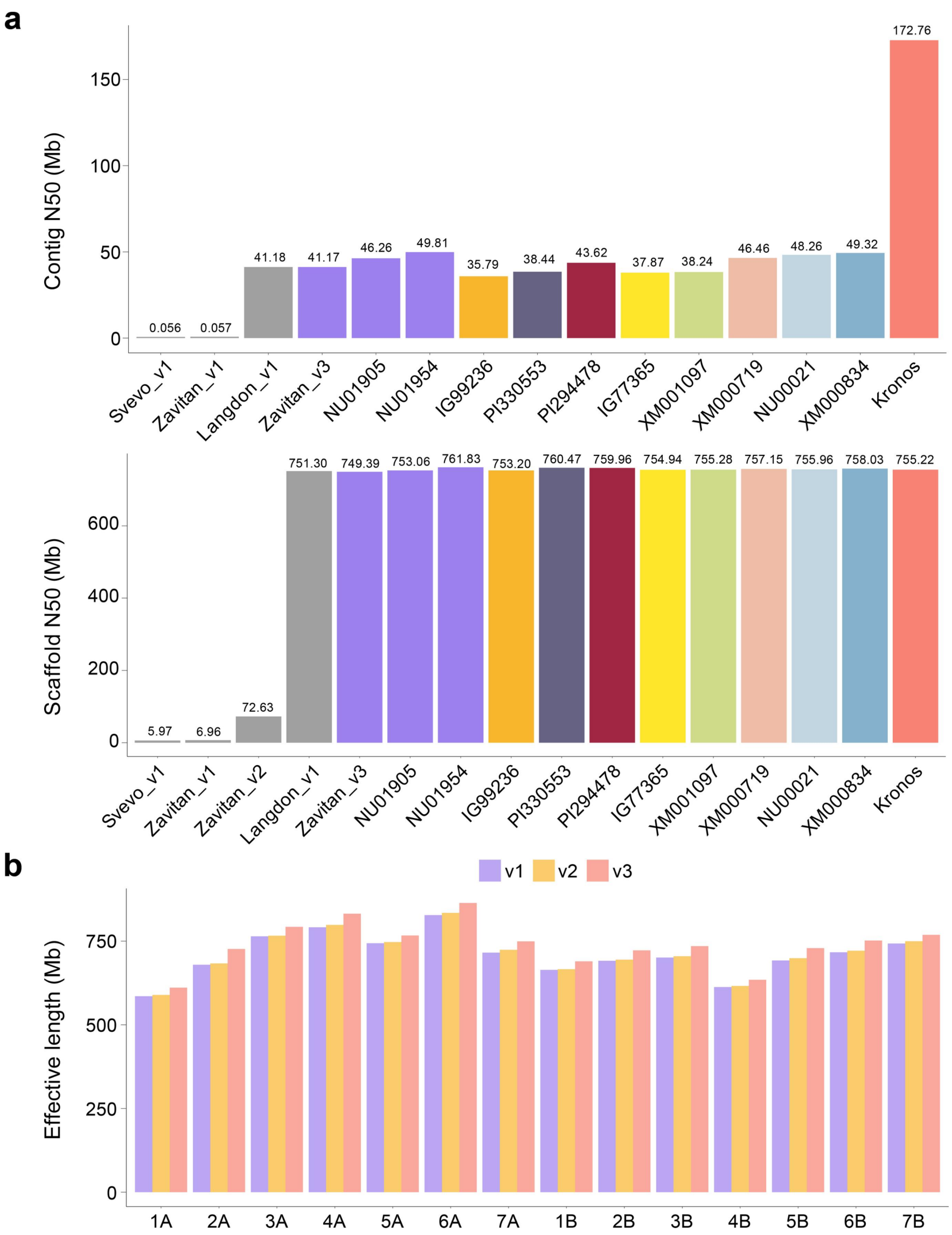

Supplementary Fig. 6 Comparison between 12 assembled genomes and published tetraploid wheat genomes. a, Comparison of the contig N50 (top) and scaffold N50 (bottom) lengths between published Svevo, Zavitan, Langdon genomes and the genomes assembled in this study. **b,** Effective chromosome lengths (genome sequence without ‘N’) in different versions of the Zavitan genome assembly. Svevo_V1 and Zavitan_V1: assembled from short-read sequencing data; Zavitan_V2: updated from Zavitan_V1 using optical maps for scaffolding; Langdon_V1: assembled from HiFi sequencing data; Zavitan_V3: generated in this study.

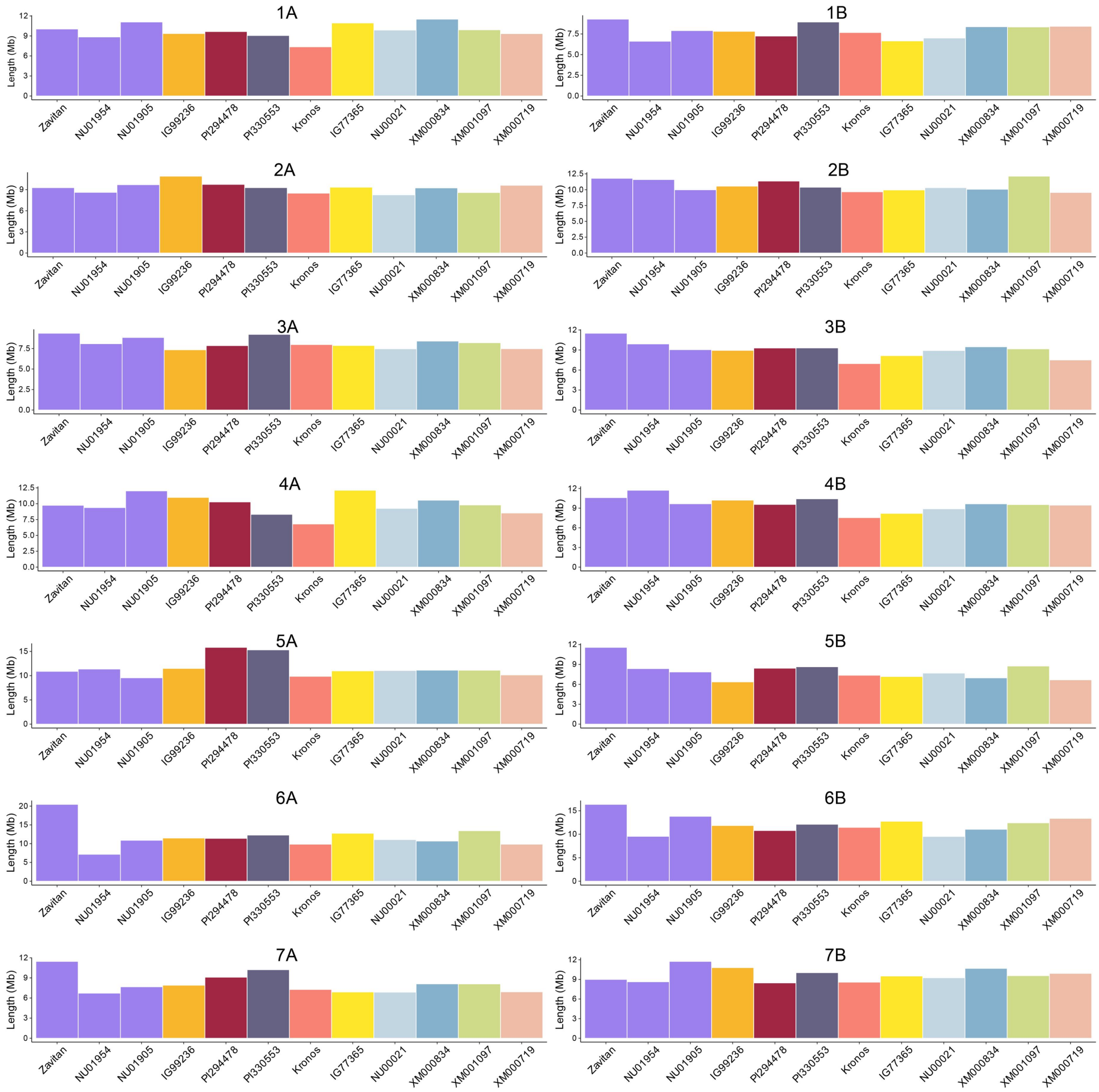

Supplementary Fig. 7 Annotated centromere length for each chromosome for the 12 assembled tetraploid wheat genomes.

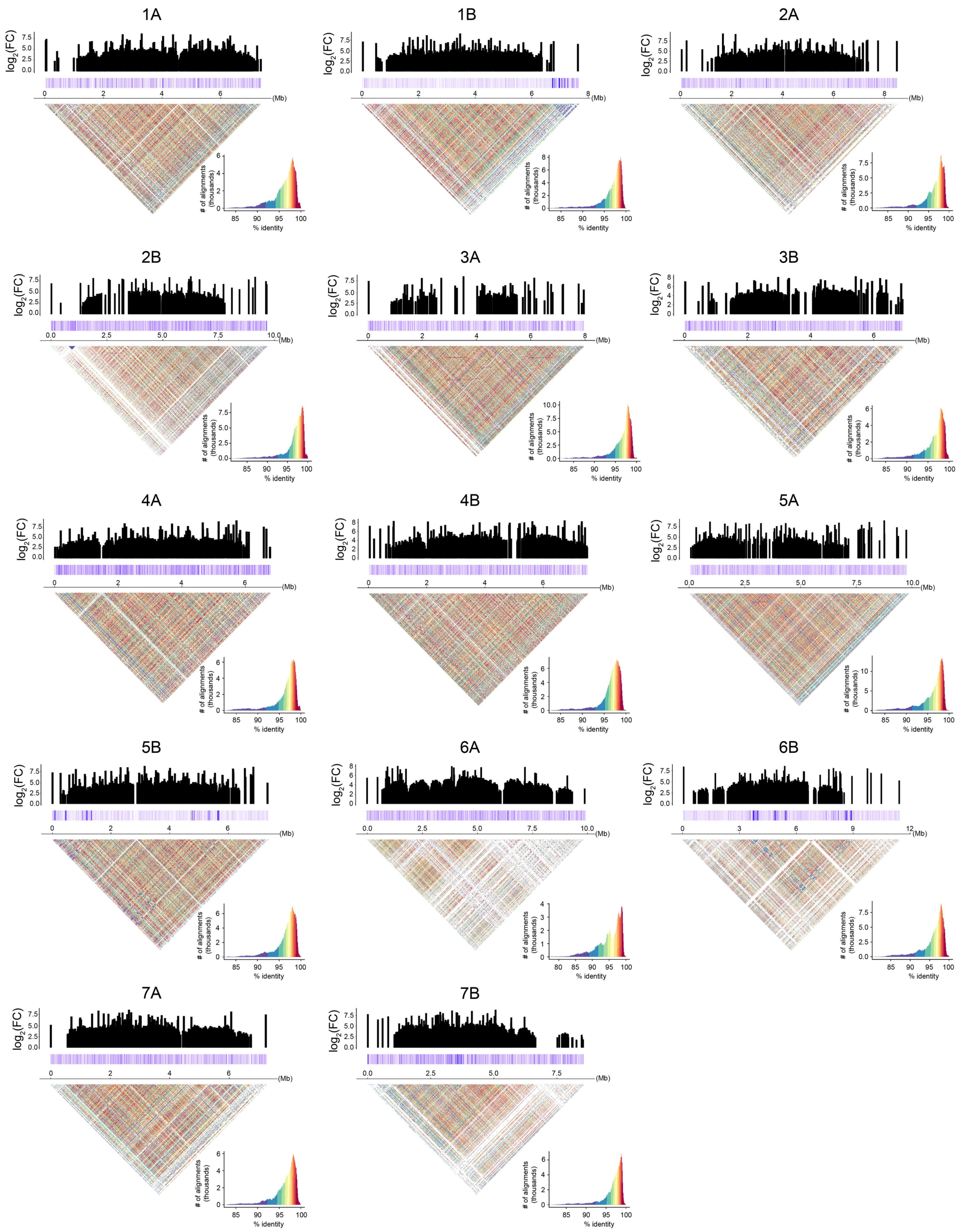

Supplementary Fig. 8 Centromere structures for *cv.* Kronos. CENH3 log2 (ChIP/input; black peak) is plotted over centromeres. The second rows show the distribution of TEs. Heatmaps show pairwise sequence identity between all non-overlapping 10-kb regions.

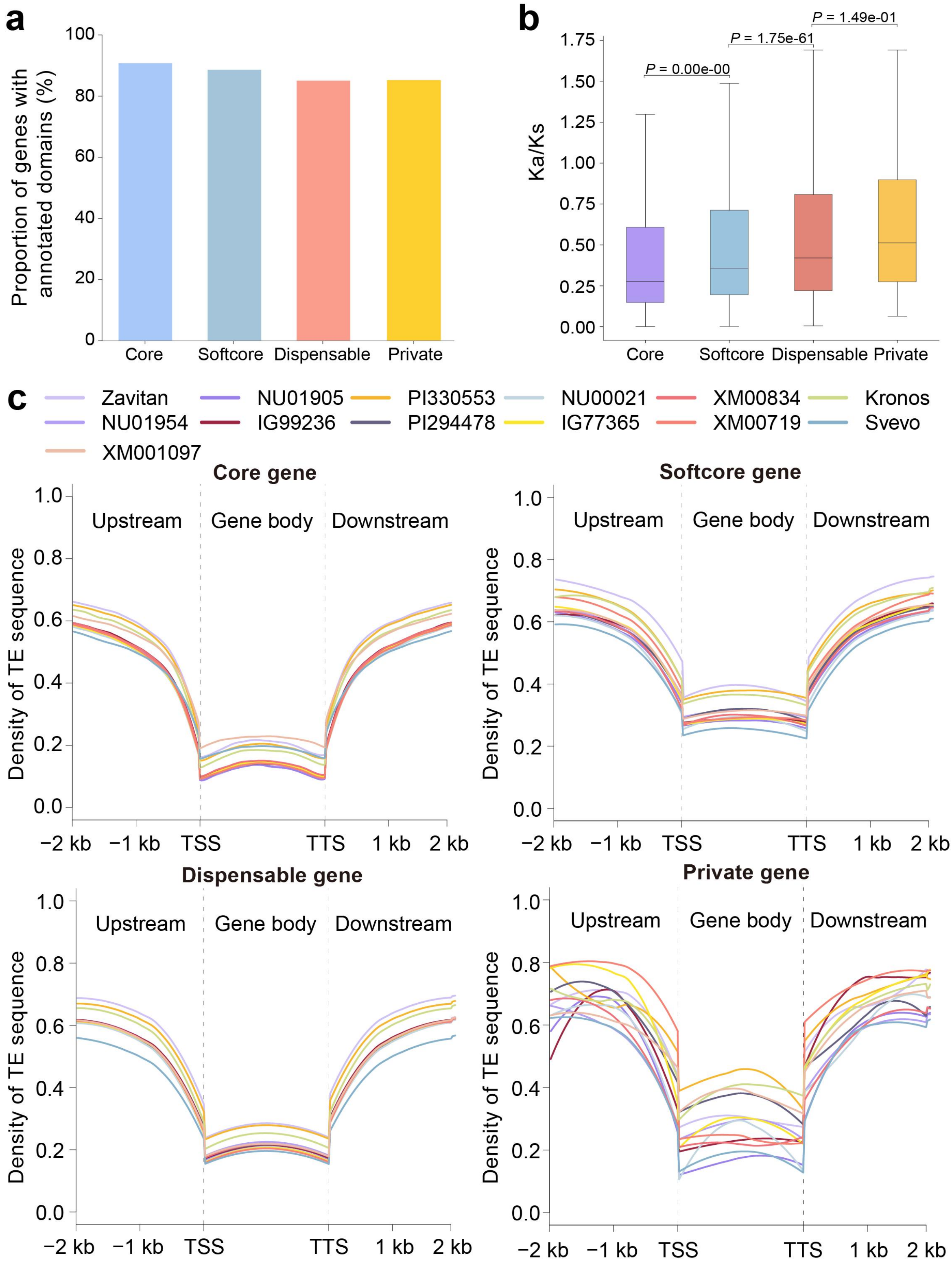

Supplementary Fig. 9 Pan-genome analysis of 13 tetraploid wheat genomes. a, Proportion of core, dispensable and private genes with conserved protein domains. **b,** Comparison of Ka/Ks values of core, dispensable and private genes using barley (*Hordeum vulgare*) as a reference. Statistical significance was determined by

Kruskal–Wallis testing and post-hoc two-sided Wilcoxon rank-sum tests with Bonferroni-corrected for multiple tests. **c,** Density of transposable-element (TE) sequences in gene bodies and 2-kb flanking regions in the 13 sequenced tetraploid wheat genomes. Center line, median; box lower and upper edges, 25% and 75% quartiles, respectively; whiskers, 1.5× IQR; colored dots, outliers. IQR, interquartile range.

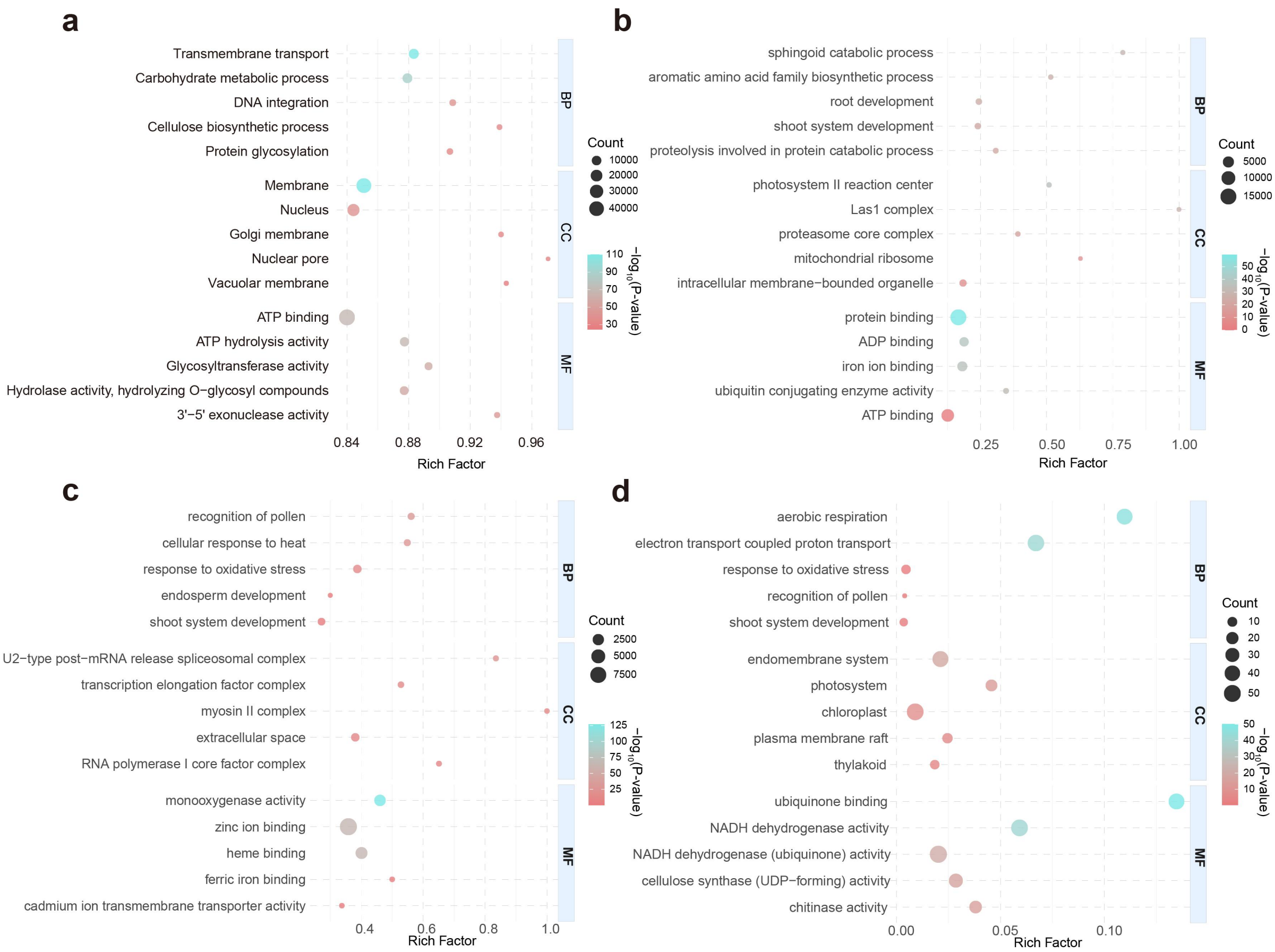

Supplementary Fig. 10 Gene-ontology enrichment for core (a), softcore (b), dispensable (c) and private (d) genes. Rich factor, the ratio of the number of transcripts located in a specific pathway to the total number of annotated transcripts belonging to that pathway.

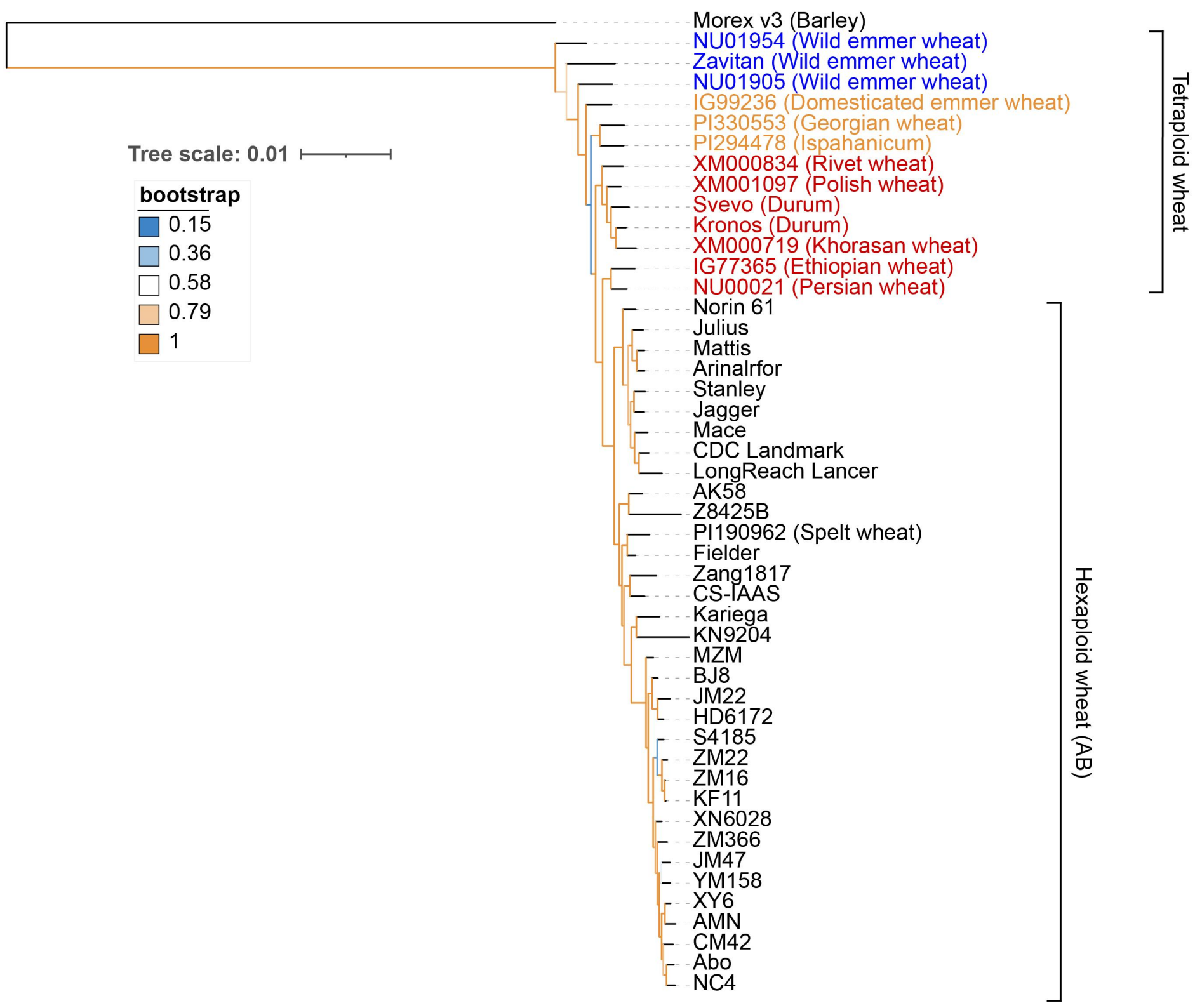

Supplementary Fig. 11 Evolutionary tree among BBAA genomes of tetraploid and hexaploid bread wheats.

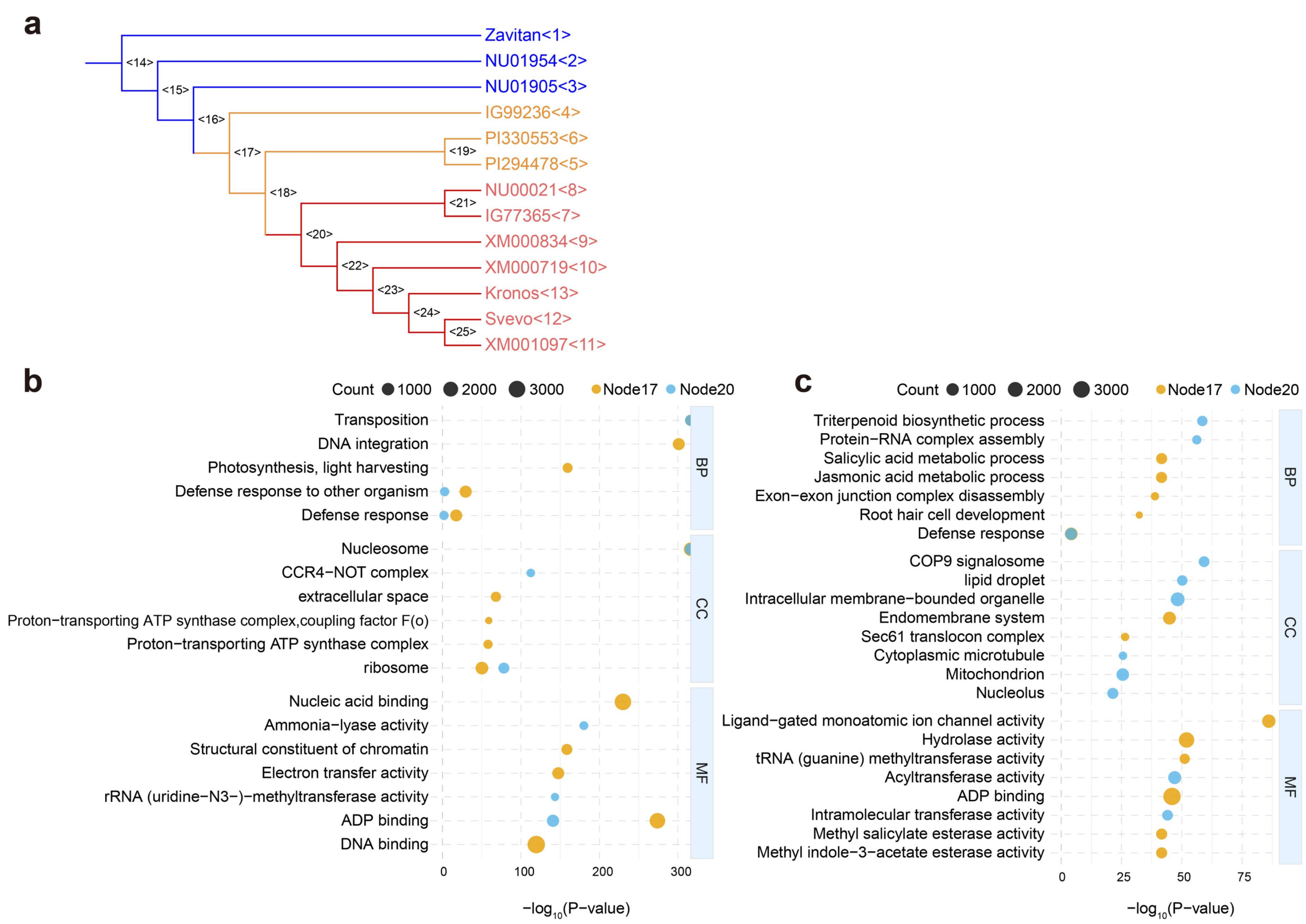

Supplementary Fig. 12 Gene-ontology enrichment for expanded and contracted gene families in key evolutionary nodes. a, Phylogenetic tree with evolution nodes. Numbers at nodes (e.g., <1>) are used for referencing clades. Tree structure is the same as in Fig. 2f. **b,** GO enrichment for expanded gene families in nodes 17 and 20. Node 17 corresponds to the most-recent common ancestor of domesticated tetraploid wheat and free-threshing tetraploid wheat, following their divergence from wild emmer wheat. Node 20 corresponds to the divergence from hulled tetraploid wheat to free-threshing tetraploid wheats. **c,** GO enrichment for contracted gene families in nodes 17 and 20.

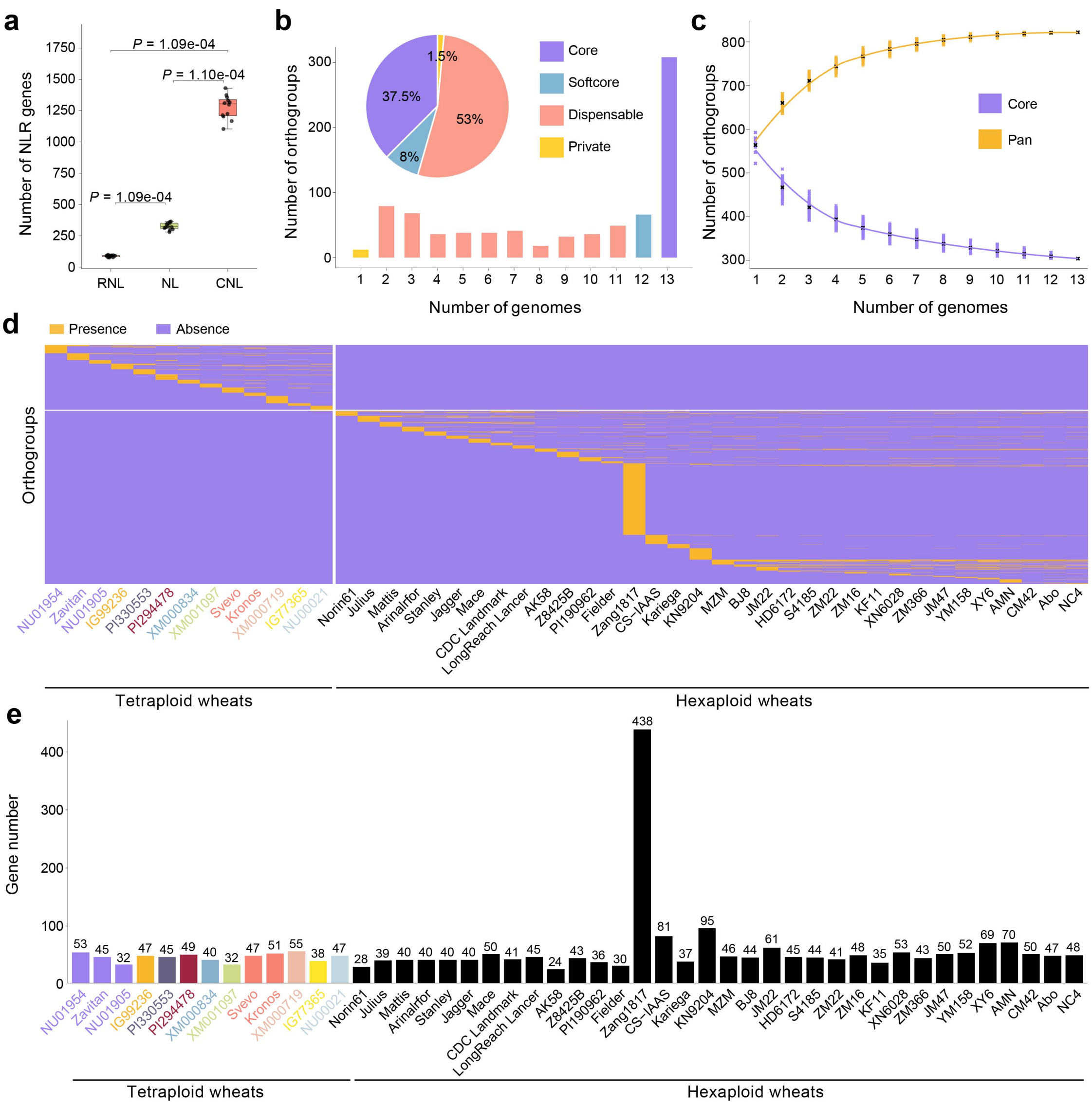

Supplementary Fig. 13 Pan-NLRomes of tetraploid wheat. a, The number of different classes of NLR genes in the 13 assembled accessions. Statistical significance was determined by Kruskal–Wallis testing and post-hoc two-sided Wilcoxon rank-sum tests with Bonferroni correction for multiple tests. **b,** Construction of a Pan-NLRome in tetraploid wheat. The histogram shows the frequency of NLR orthogroups. **c,** Number of pan and core NLR orthogroups across 1-13 genomes (including the published Svevo genome). **d,** Heatmap of specific NLR orthogroups between tetraploid and hexaploid wheats. Purple and orange depict the presence and absence of NLR orthogroups, respectively. **e,** NLR gene numbers in each *de novo* assembled tetraploid wheat and hexaploid wheat accession. Center line,

median; box lower and upper edges, 25% and 75% quartiles, respectively; whiskers, 1.5× IQR; colored dots, outliers. IQR, interquartile range.

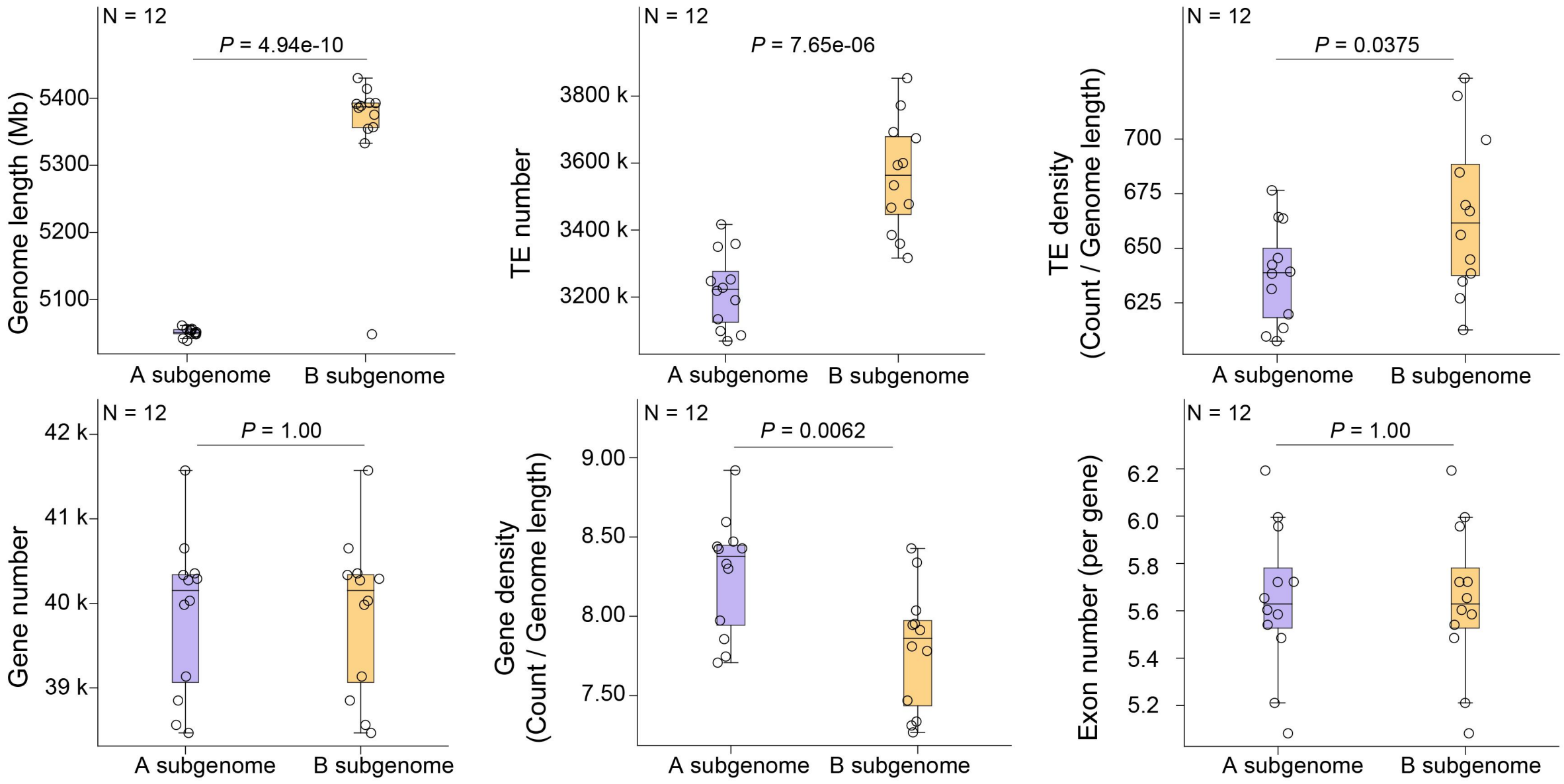

Supplementary Fig. 14 The A and B subgenomes of tetraploid wheat are asymmetrical. Genome length, TE density, and gene number of the B subgenome are greater than those of the A subgenome, while gene density is lower than that of the A subgenome. Statistical significance was determined by two-sided t-tests. Center line, median; box lower and upper edges, 25% and 75% quartiles, respectively; whiskers, 1.5× IQR; colored dots, outliers. IQR, interquartile range.

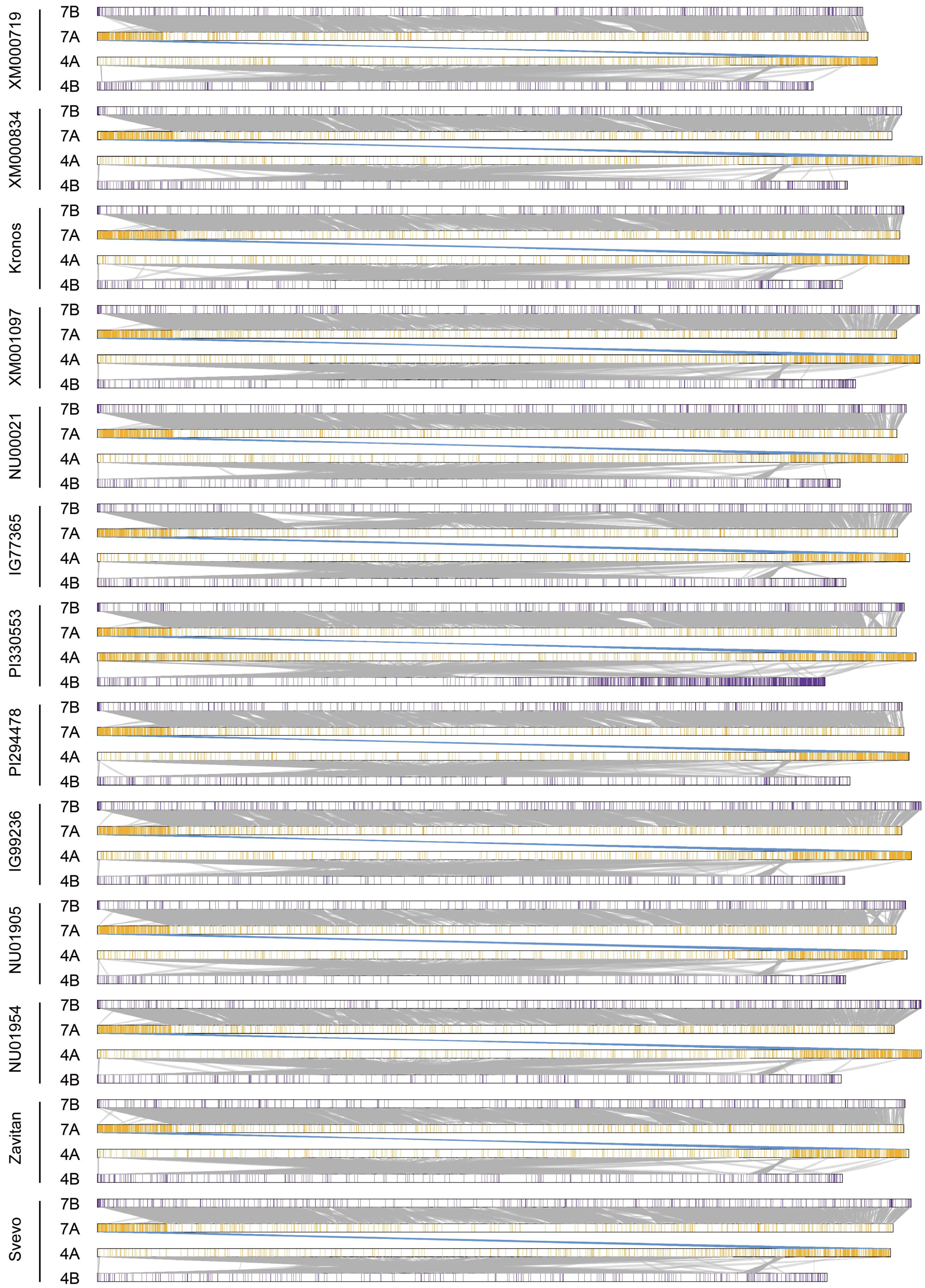

Supplementary Fig. 15 Distribution of singleton genes on chromosomes 4A, 4B, 7A and 7B. Yellow represents the singleton genes on the A subgenomes; purple represents the singleton genes on the B subgenomes; grey and blue lines represent synteny genes between different chromosomes.

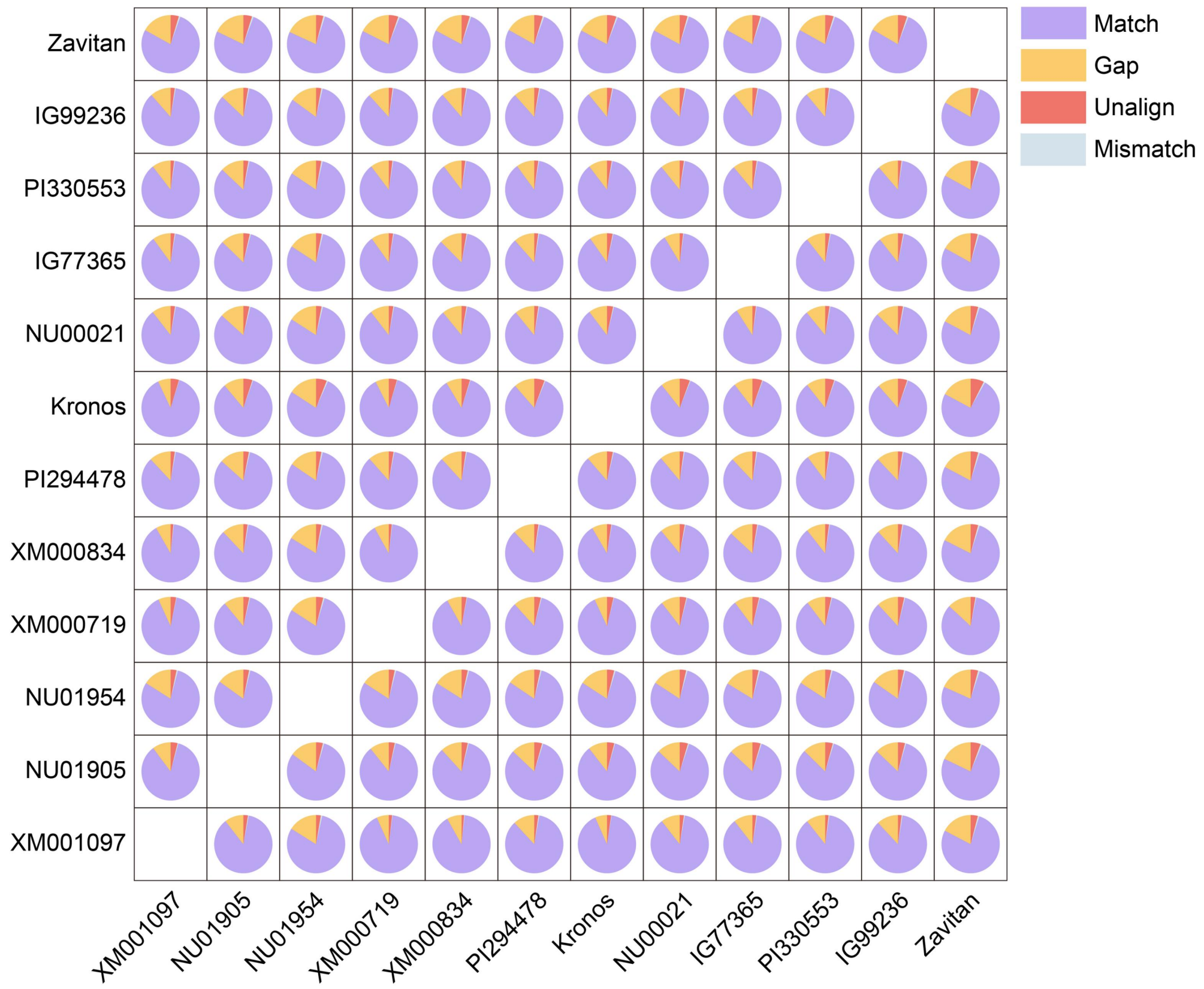

Supplementary Fig. 16 Summary of genome alignments for the 12 genomes. The upper-left corner of the rectangle shows statistics for pairwise genome-alignment results, wherein the accessions labelled on the y axis were set as references, and the x axis represents the queries. For the lower-right corner, accessions labelled on the x axis were set as references, and the y axis were queries. The proportion of each pie chart was calculated relative to each reference genome. 'Gap' indicates bases present on the reference genome, but deleted from the query genome.

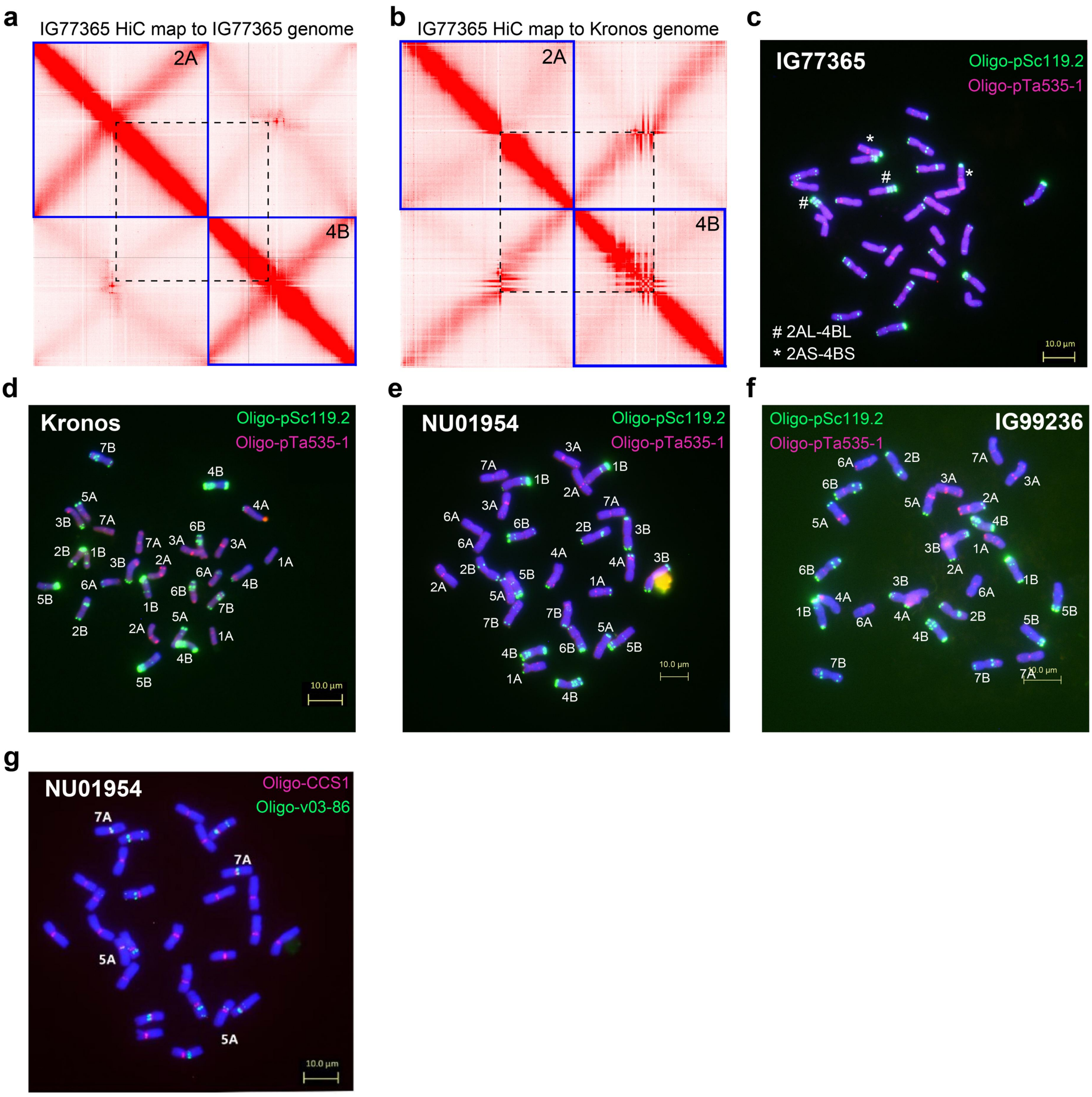

Supplementary Fig. 17 FISH reveals three large karyotype variations in IG77365 and NU00021. a–c, Hi-C heatmaps (**a, b**) and fluorescence *in situ* hybridization (FISH) (**c**) analysis confirm the translocation between chromosome 2A and 4B of accession IG77365. **d–f,** FISH analysis with Oligo-pSc119.2 and Oligo-pTa535-1 on control samples Kronos (**d**), NU1954 (**e**) and IG99236 (**f**). **g**, FISH analysis with Oligo-CCS1 and Oligo-v03-86 on control samples NU1954.

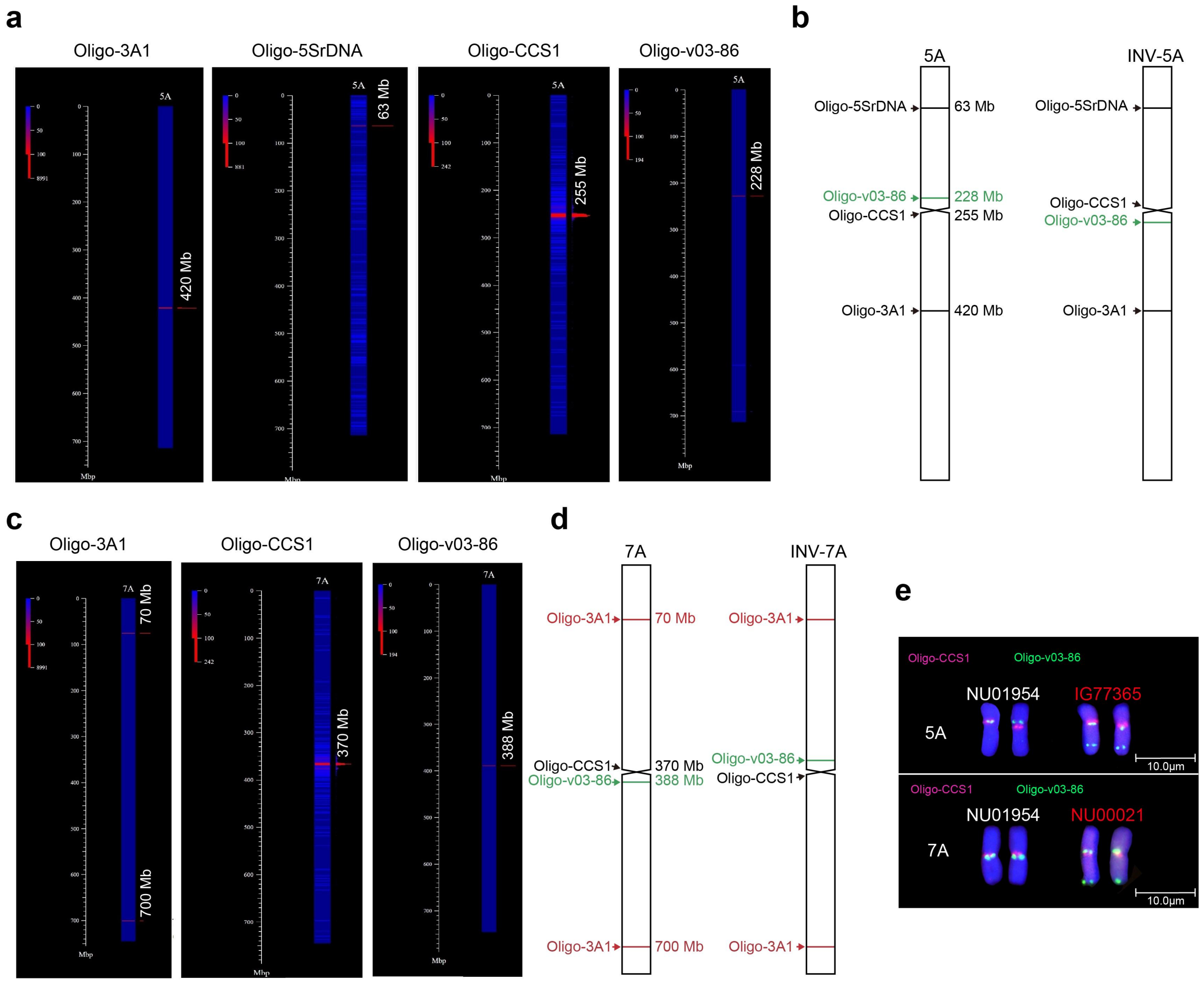

Supplementary Fig. 18 FISH identifies inversions on chromosome 5A in accession IG77365 and chromosome 7A in accession NU00021. a–b, Fluorescence signals (**a**) of oligonucleotide probes used to verify the inversion in the pericentromeric region of chromosome 5A of IG77365 on Chinese Spring chromosomes and a schematic (**b**) of the inversion variation detected on chromosome 5A. **c–d**, Fluorescence signals (**c**) of oligonucleotide probes used to verify the inversion in the pericentromeric region of chromosome 7A of NU00021 on Chinese Spring chromosomes and a schematic (**d**) of the inversion variation detected on chromosome 7A. **e,** Fluorescence signals observed in the inversion region of chromosomes 5A and 7A. NU01954 was set as the control sample.

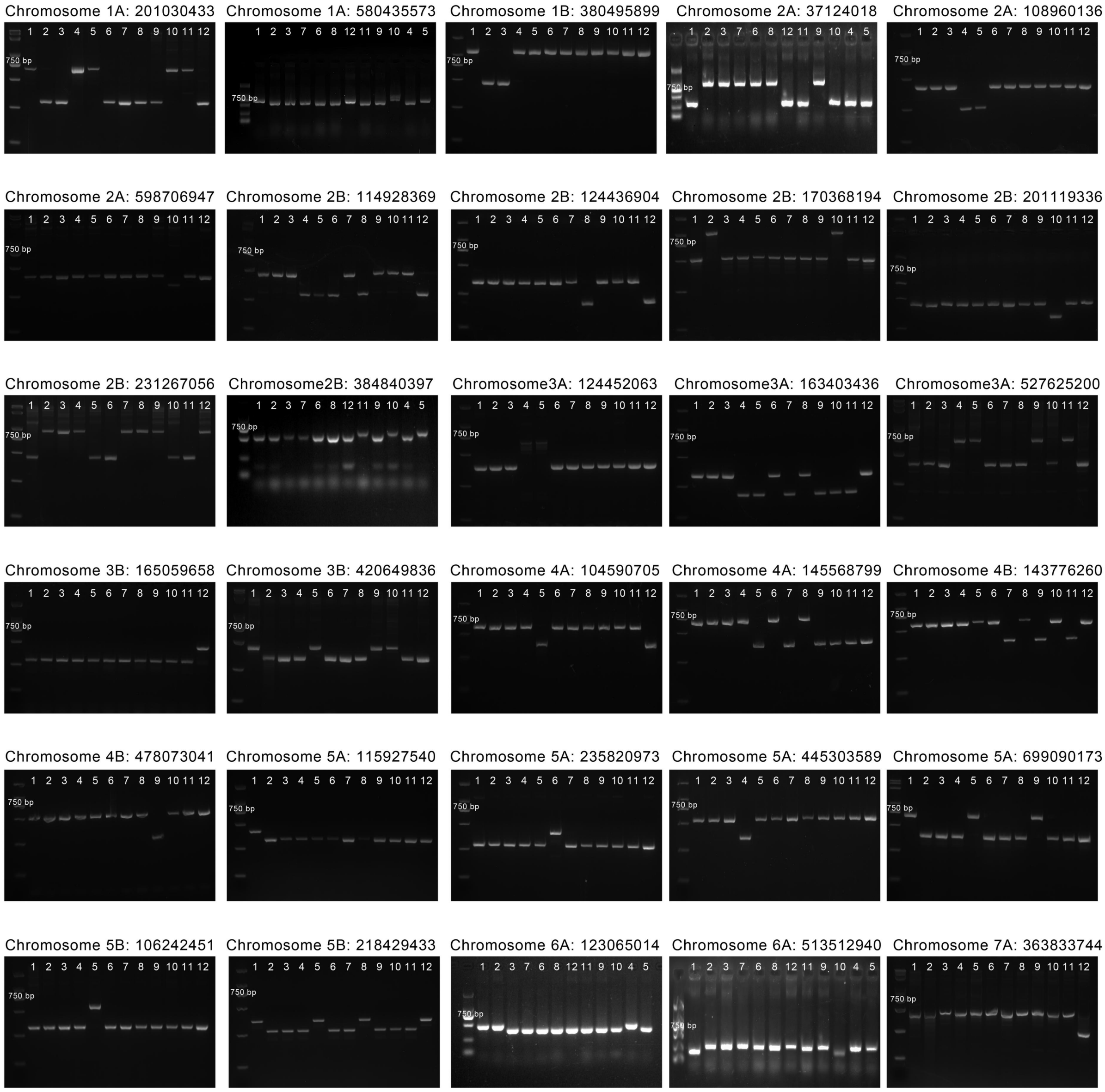

Supplementary Fig. 19 PCR verification of 30 randomly selected structural variants. DL 2000 DNA and DL 5000 DNA were used as markers. Marker and the obtained DNA ladder sizes were 100, 250, 500, 750, 1,000, 2,000, 3,000 and 5,000 bp. The number from 1 to 12 represent: 1, Zavitan; 2, Kronos; 3, XM001097; 4, NU01905; 5, NU01954; 6, XM000719; 7, XM000834; 8, IG77365; 9, PI330553; 10, IG99236; 11, PI294478; 12, NU00021 in order.

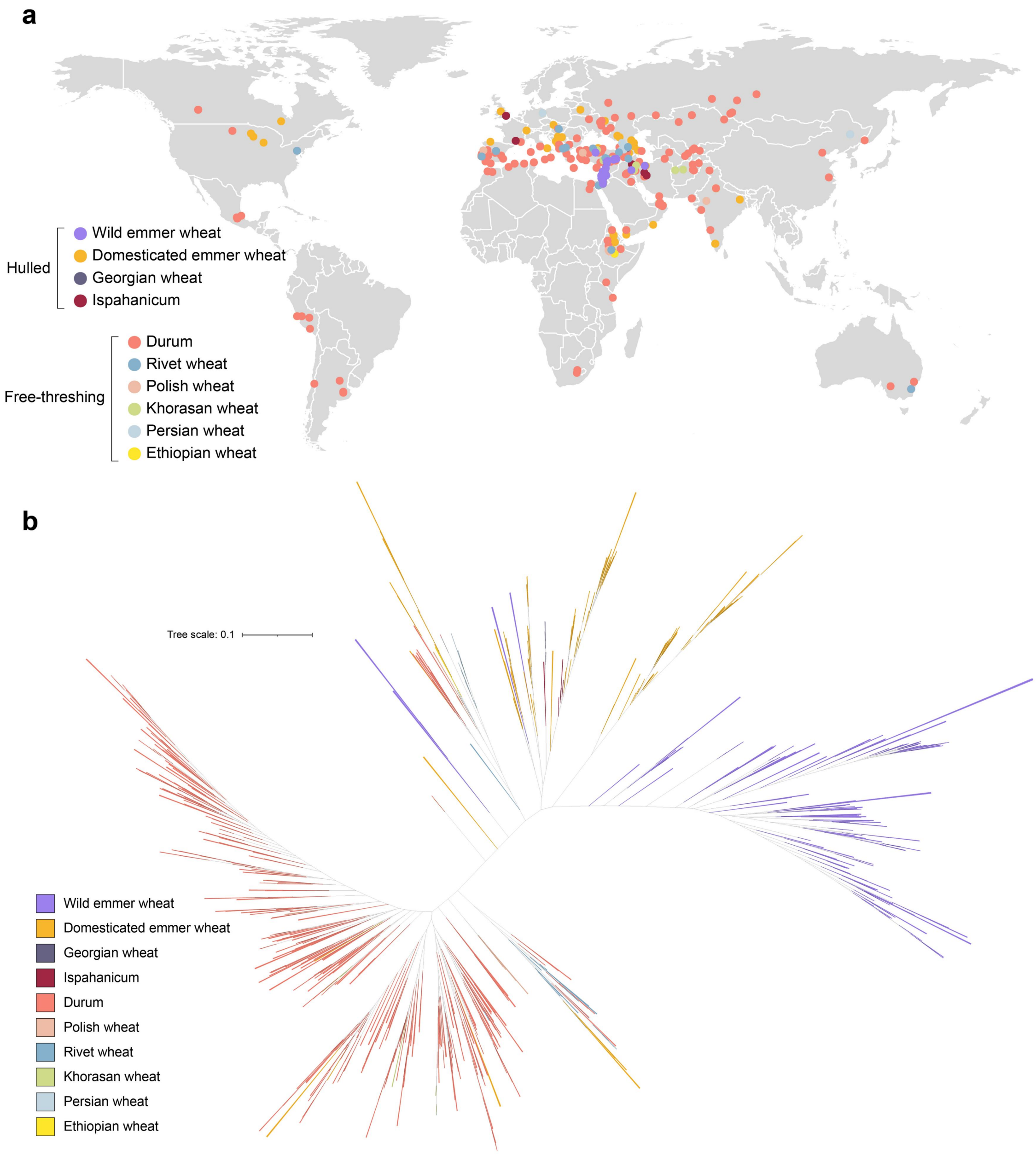

Supplementary Fig. 20 Geographic distribution and phylogenetic analysis of 736 tetraploid wheat accessions used in this study. a, 621 accessions were sequenced in this study and 121 were obtained from public data. **b,** The phylogenetic tree was constructed using the maximum-likelihood method in RAxML with 1,000 bootstraps. The map was plotted using the R package rworldmap (GPL ≥ 2 license, https://github.com/AndySouth/rworldmap), with base map data sourced from public-domain Natural Earth dataset (http://www.naturalearthdata.com).

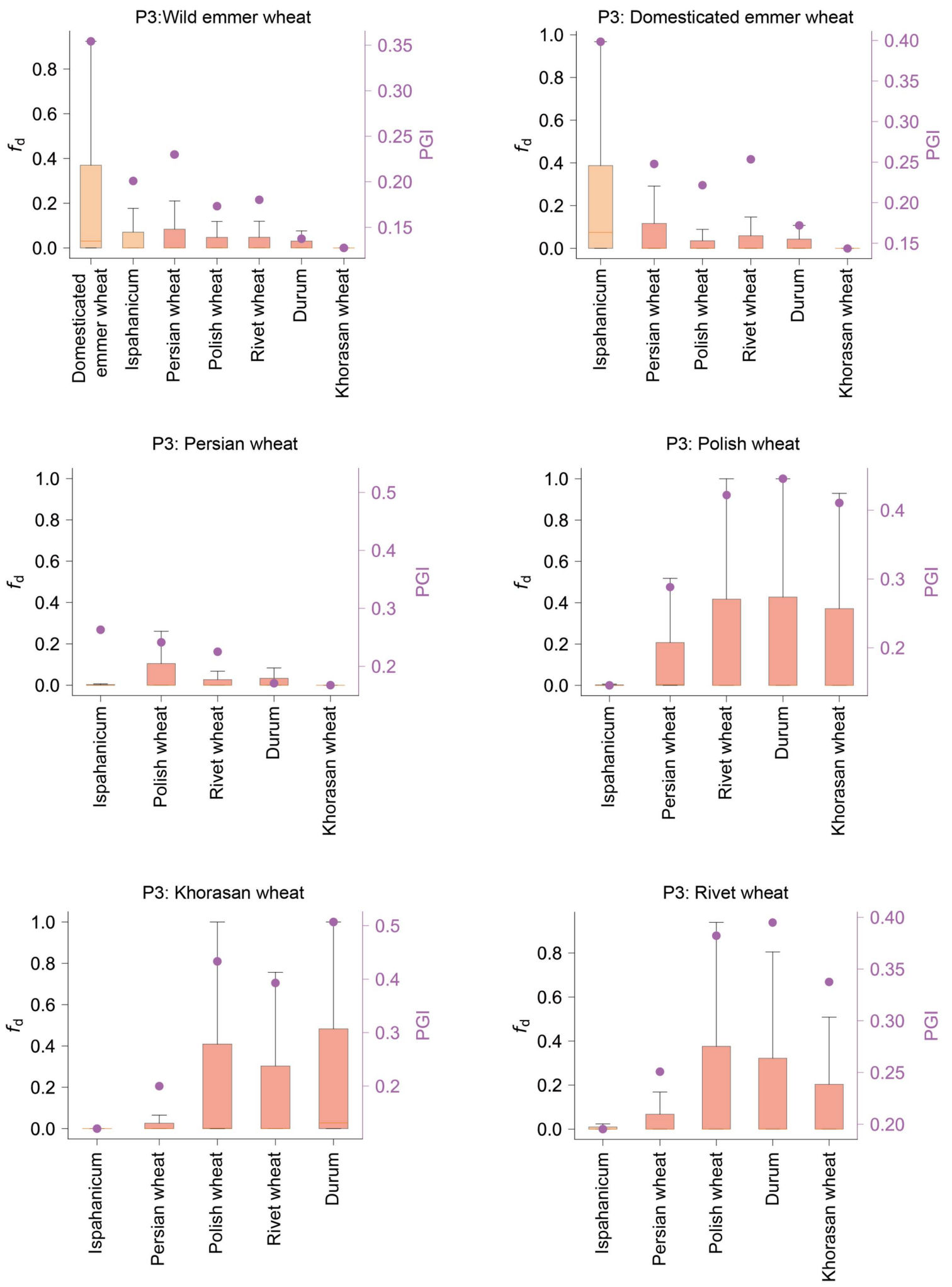

Supplementary Fig. 21 Analysis of gene flow from donor populations (P3) to recipient populations (P2) using Georgian wheat as the reference (P1). Box plots display f_d statistics (left y axis) across all sliding windows from P3 to P2 (x axis), with purple dots representing the relative proportion of genome introgression (PGI) (right y axis), i.e., the percentage of gene introgression in a given population relative to the local variety's PGI, corresponding to the right y axis.

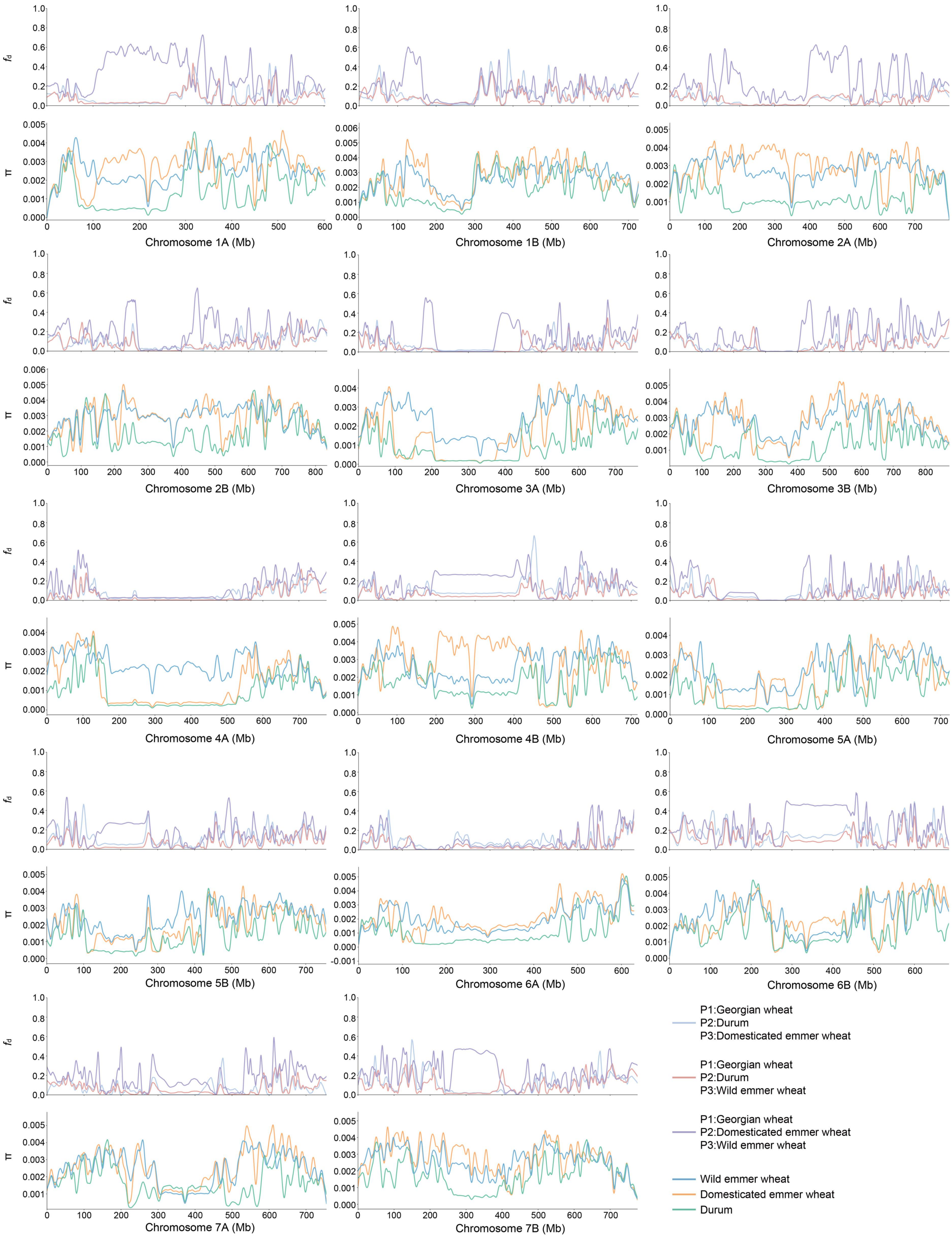

Supplementary Fig. 22 Putative potential introgression (f_d) and nucleotide diversity (π) across each chromosome. The size and frequency of potential introgression fragments differ in P2 accessions. Potential introgression fragments

frequently appear in the vicinity of the centromere region, spanning hundreds of megabases. For example, the longest potential introgression segment on chromosome 1A (about 82–260 Mb) from wild emmer wheat to domesticated tetraploid wheat is around the centromere region (about 215–222 Mb) (Supplementary Table 3).

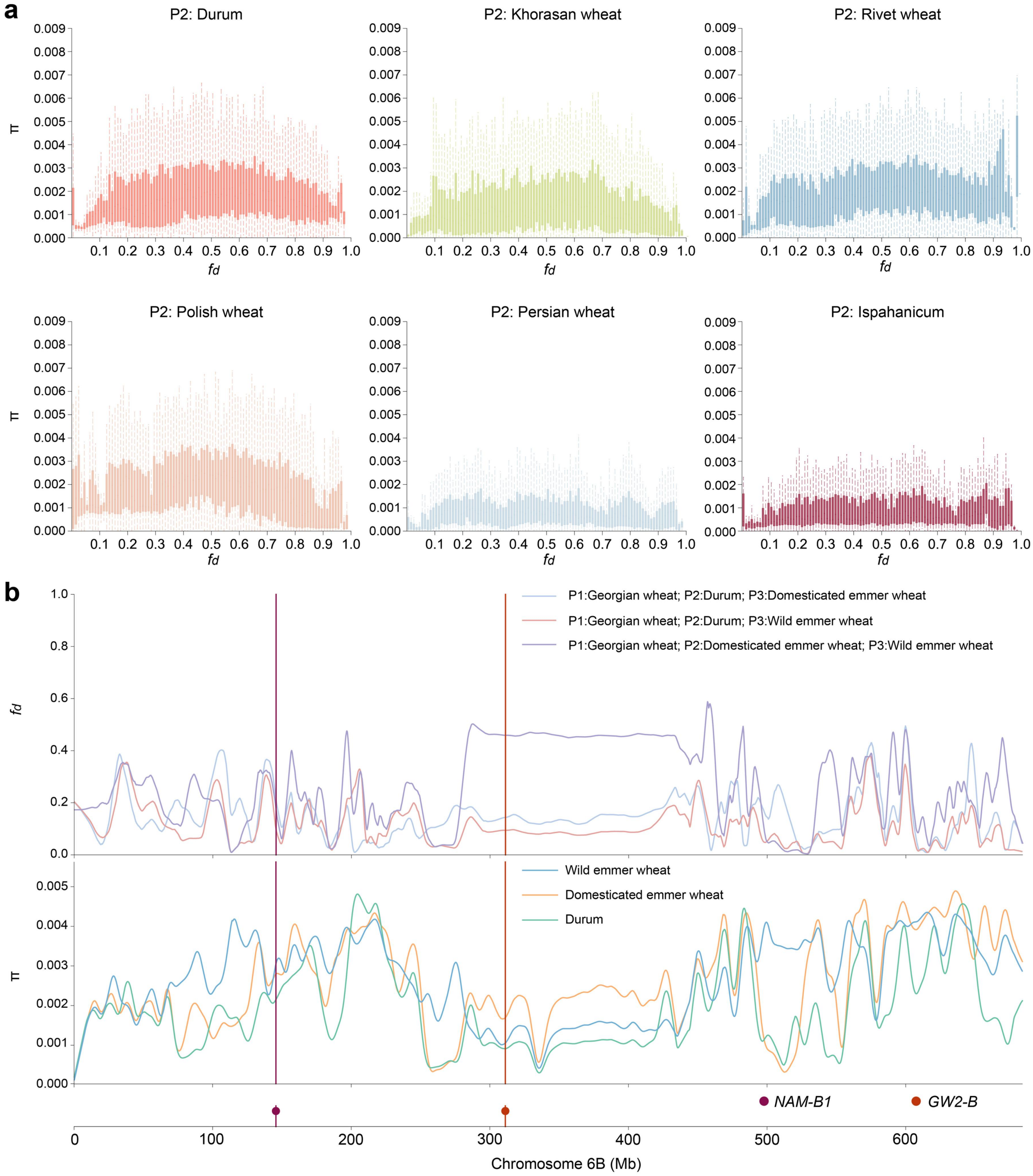

Supplementary Fig. 23 The relationship between introgression (f_d) and nucleotide diversity (π) among free-threshing tetraploid wheats. a, Non-monotonic pattern of f_d and π. **b,** An example of a putative introgression region on chromosome 6B.

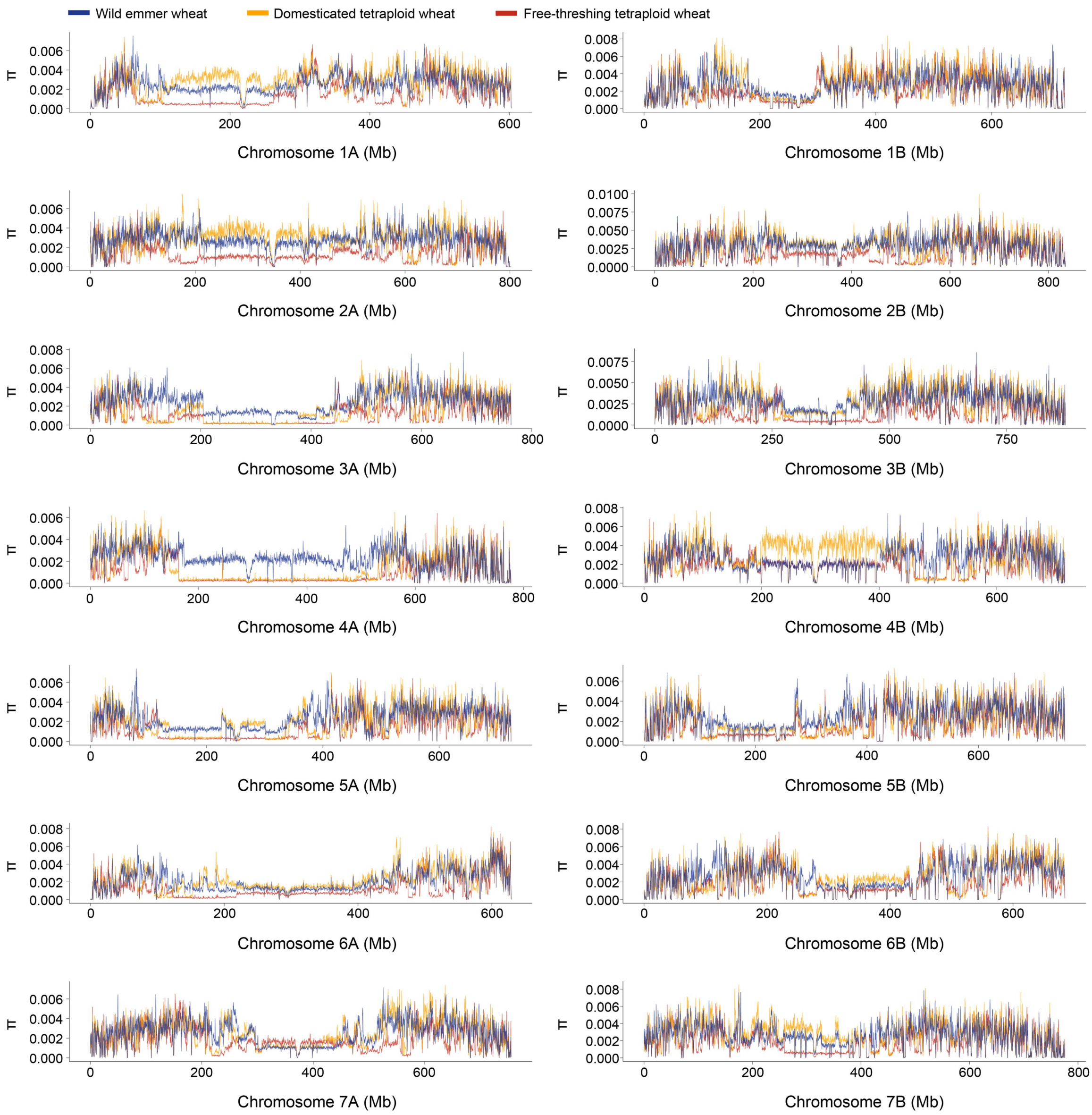

Supplementary Fig. 24 Nucleotide diversity of wild emmer wheat, domesticated tetraploid wheat and free-threshing tetraploid wheat.

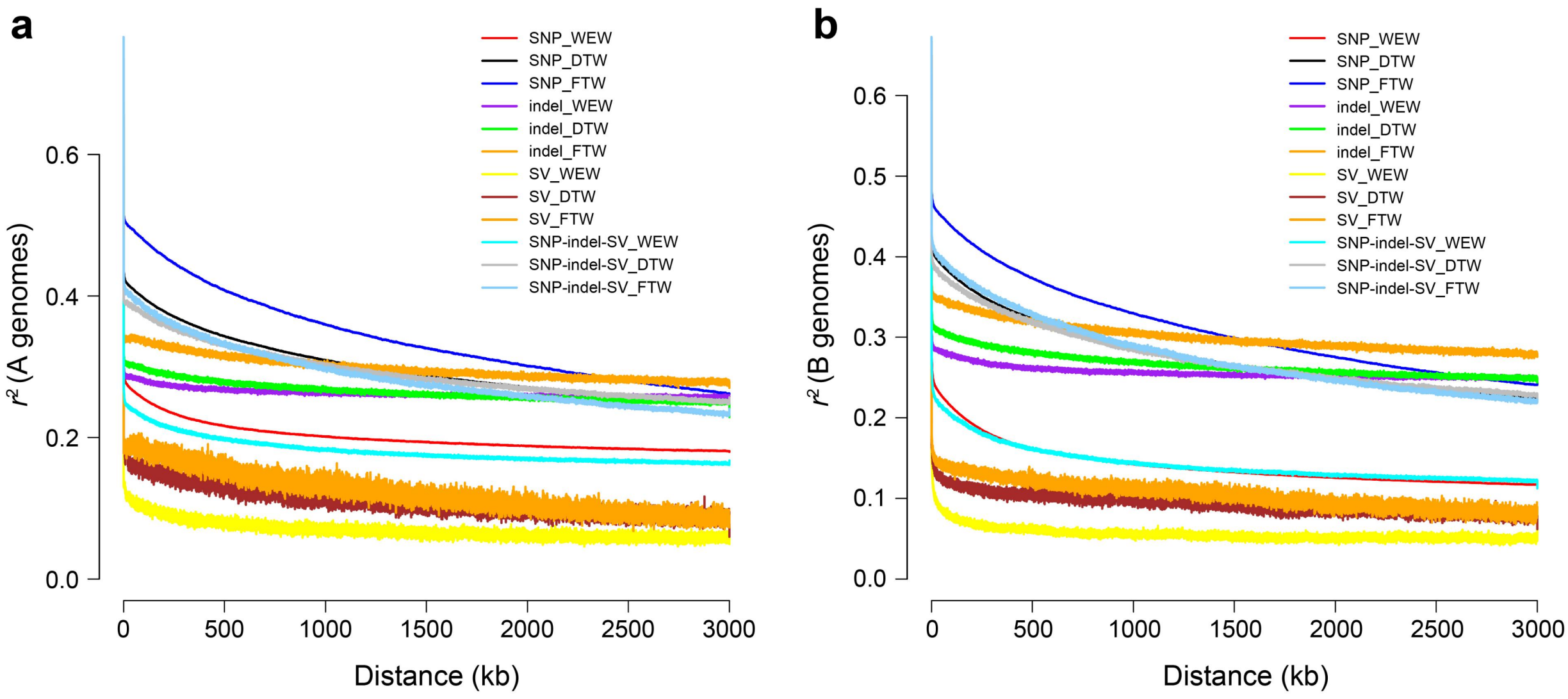

Supplementary Fig. 25 Linkage-disequilibrium-decay analysis for different types of genomic variation. a, Linkage-disequilibrium decay in the A subgenome. **b,** Linkage-disequilibrium decay in the B subgenome. WEW, wild emmer wheat; DTW, domesticated tetraploid wheat; FTW, free-threshing tetraploid wheat.

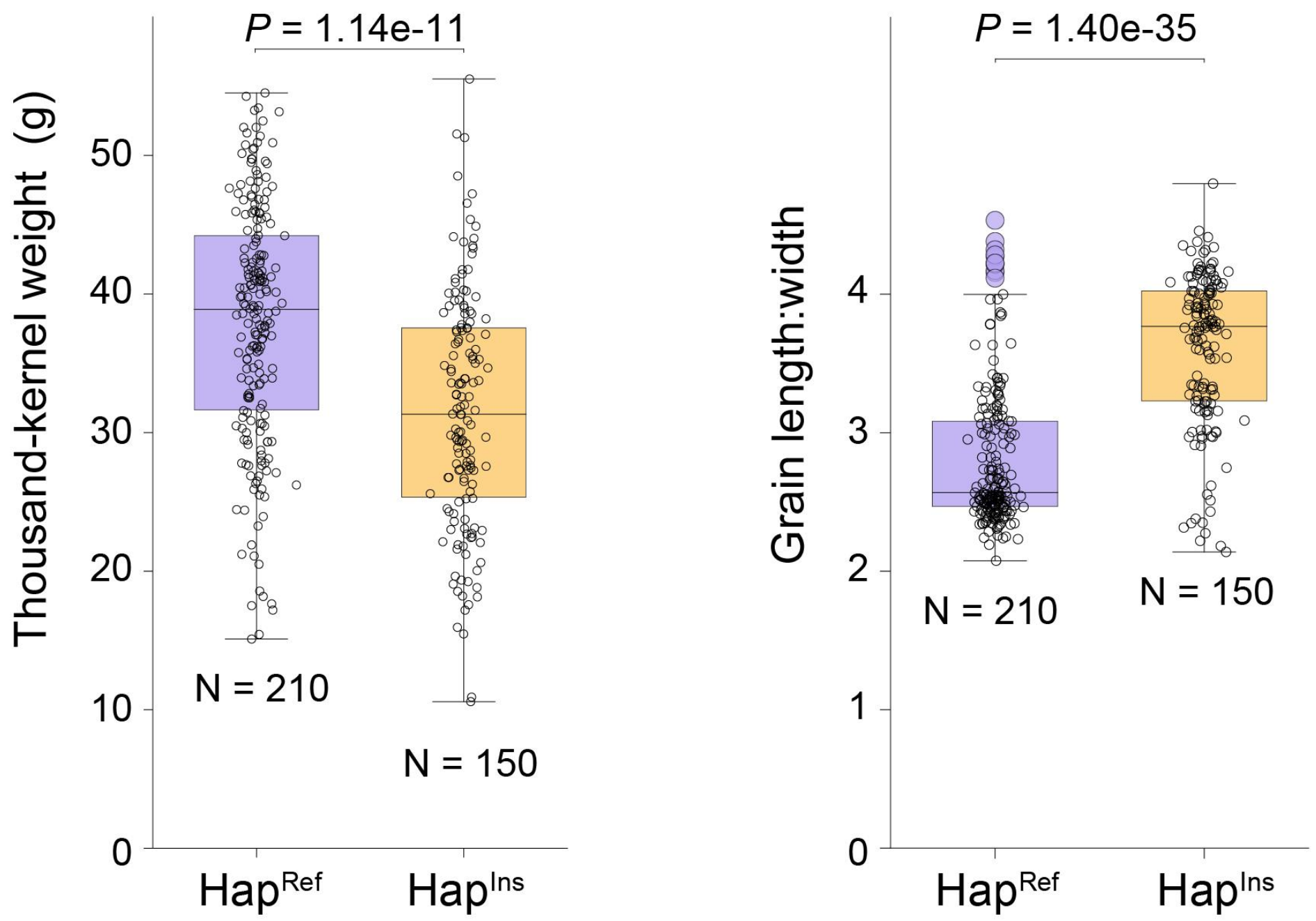

Supplementary Fig. 26 Phenotypes of tetraploid wheat individuals with two *TtGRAS-A* haplotypes. Statistical significance was determined by two-sided t-tests. Center line, median; box lower and upper edges, 25% and 75% quartiles, respectively; whiskers, 1.5× IQR; colored dots, outliers. IQR, interquartile range.

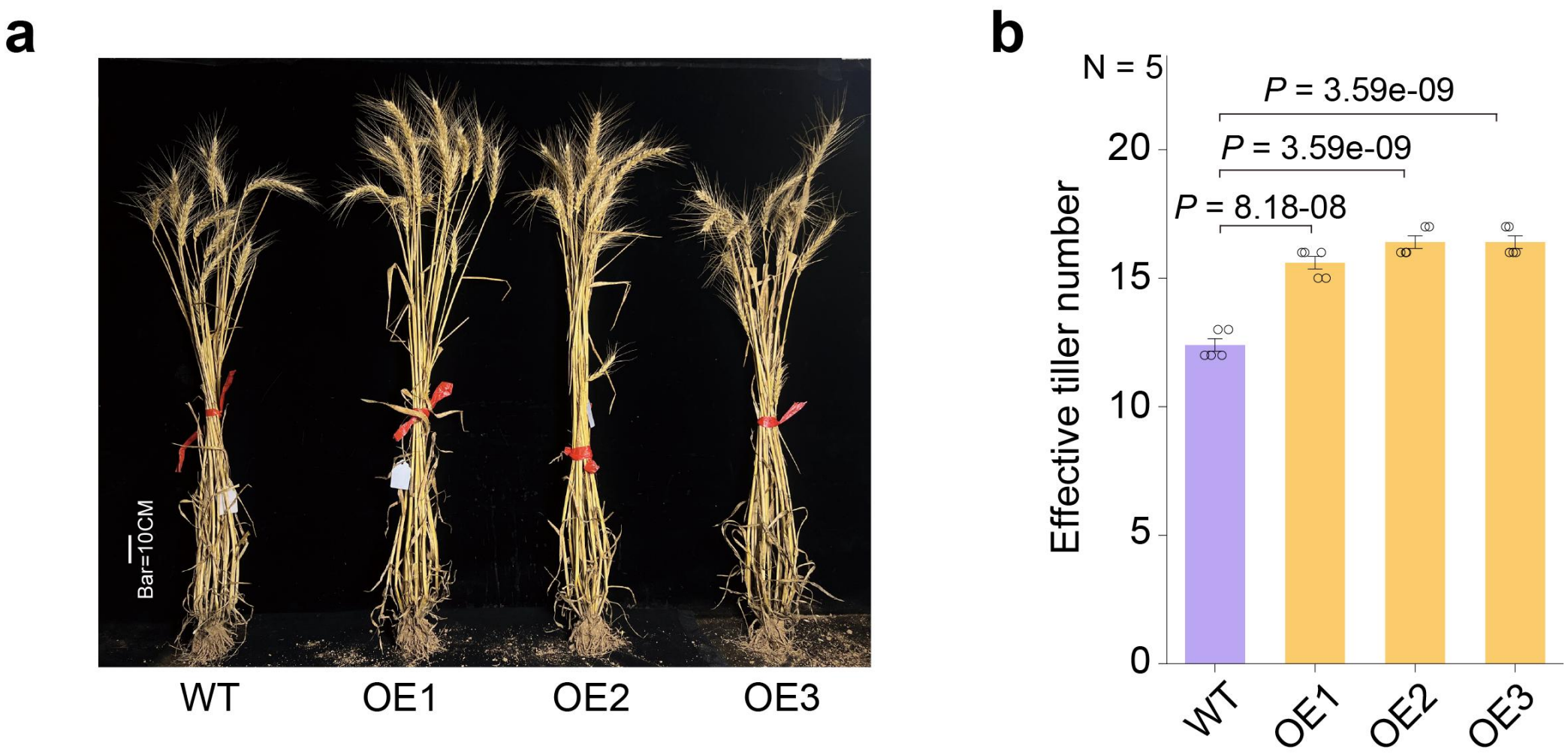

Supplementary Fig. 27 Phenotype (a) and statistical analysis (b) of effective tiller number in *TtGRAS-A* overexpression lines. Statistical significance was determined by one-way ANOVA followed by Fisher's least-significant difference multiple-comparisons tests. Error bars are mean ± s.e.m.

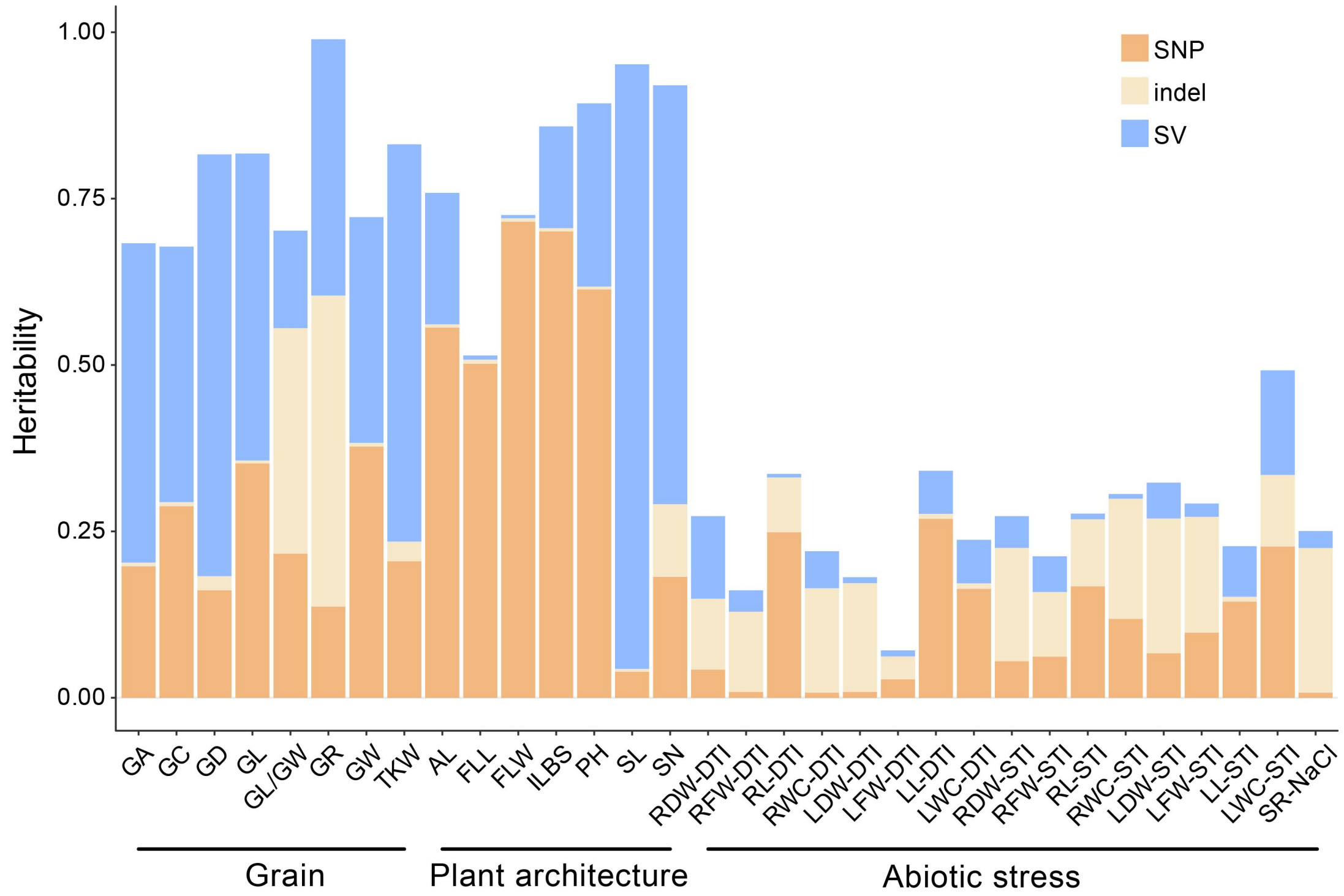

Supplementary Fig. 28 Heritability of 32 traits contributed by genomic structural variants (SVs), SNPs and indels. GA, grain area; GC, grain circumference; GD, grain diameter; GL, grain length; GL/GW, ratio of GL to GW; GR, grain roundness; GW, grain width; TKW, thousand-kernel weight; AL, awn length; FLL, flag-leaf length; FLW, flag-leaf width; ILBS, internode length below spike; PH, plant height; SL, spike length; SN, spikelet number per spike; STI, salt tolerance index; DTI, drought tolerance index; RDW, root dry weight; RFW, root fresh weight; RL, root length; RWC, root water content; LDW, leaf dry weight; LFW, leaf fresh weight; LL, leaf length; LWC, leaf water content; SR, survival rate.

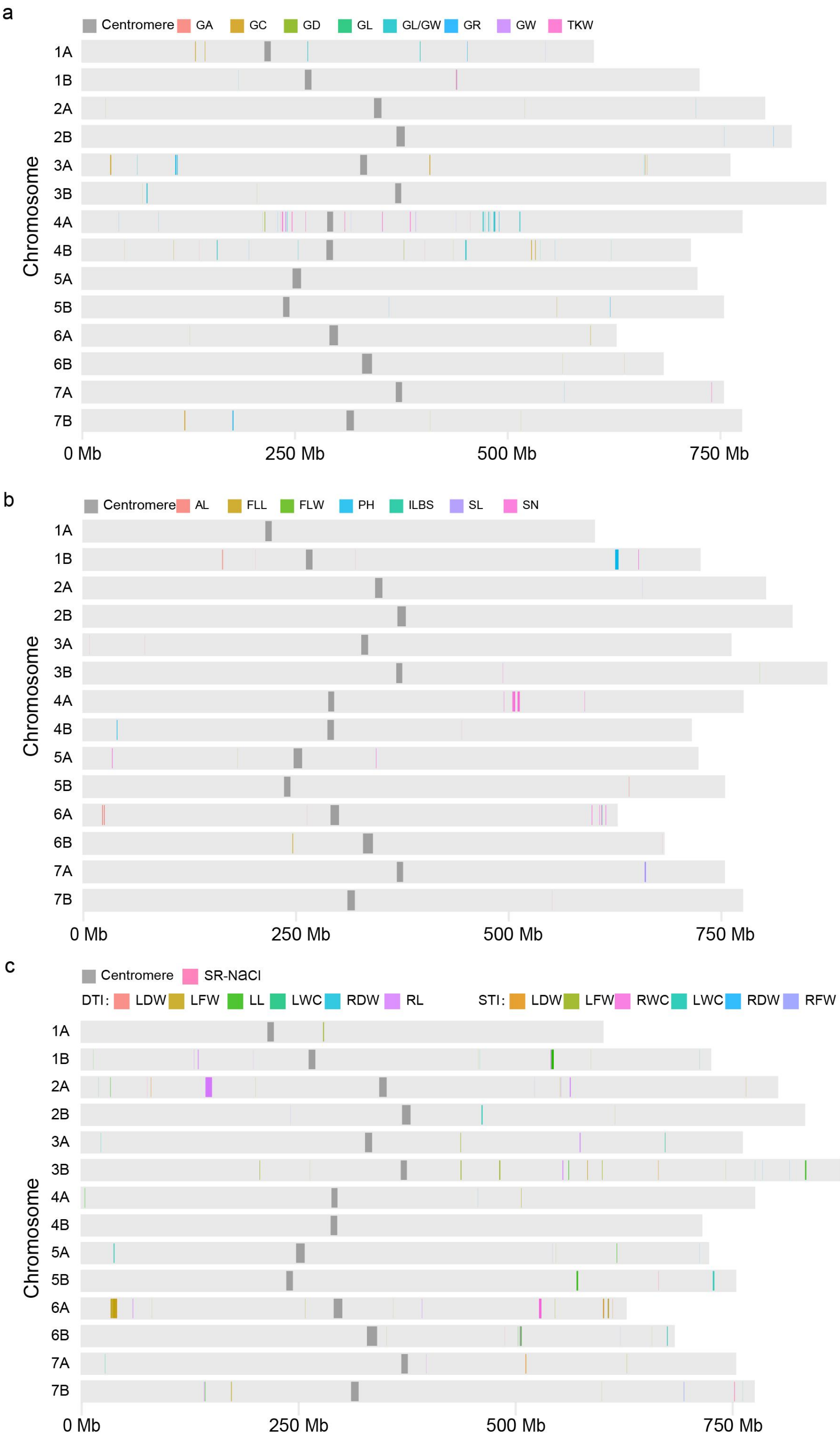

Supplementary Fig. 29 Chromosomal distribution of QTLs related to grain (a), plant-architecture (b) and abiotic-stress traits (c). GA, grain area; GC, grain circumference; GL, grain length; GW, grain width; GD, grain diameter; GR, grain

roundness; GL/GW, ratio of GL to GW; TKW, thousand-kernel weight; AL, awn length; FLW, flag-leaf width; FLL, flag-leaf length; PH, plant height; ILBS, internode length below spike; SN, spikelet number; SL, spike length; STI, salt-tolerance index; DTI, drought-tolerance index; SR-NaCl, Survival rate−NaCl; LDW, Leaf dry weight; LFW, Leaf fresh weight; LL, Leaf length; RWC, Root water content; LWC, Leaf fresh weight; RDW, Root dry weight; RL, Root length; RFW, Root fresh weight.

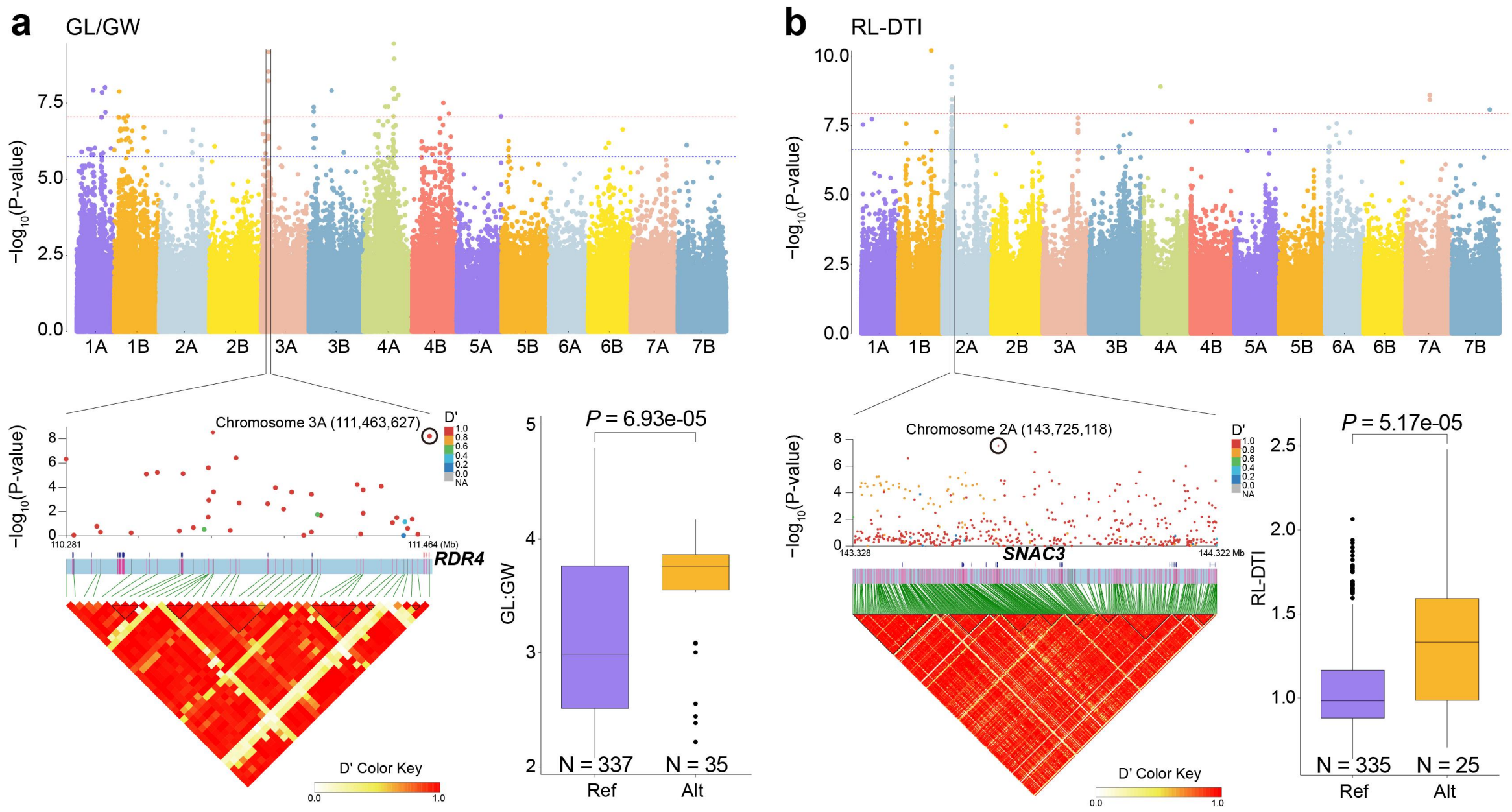

Supplementary Fig. 30. Genome-wide association study for grain length/width and drought tolerance. GWAS results for grain length/width (**a**) and RL-DTI (**b**) are shown as Manhattan plots. Linkage disequilibrium is in the focus region and the comparison of phenotypes grouped by the alleles at the most strongly associated genomic variants at the focus loci. Statistical significance was determined by two-sided t-tests. Center line, median; box lower and upper edges, 25% and 75% quartiles, respectively; whiskers, 1.5× IQR; colored dots, outliers. IQR, interquartile range.

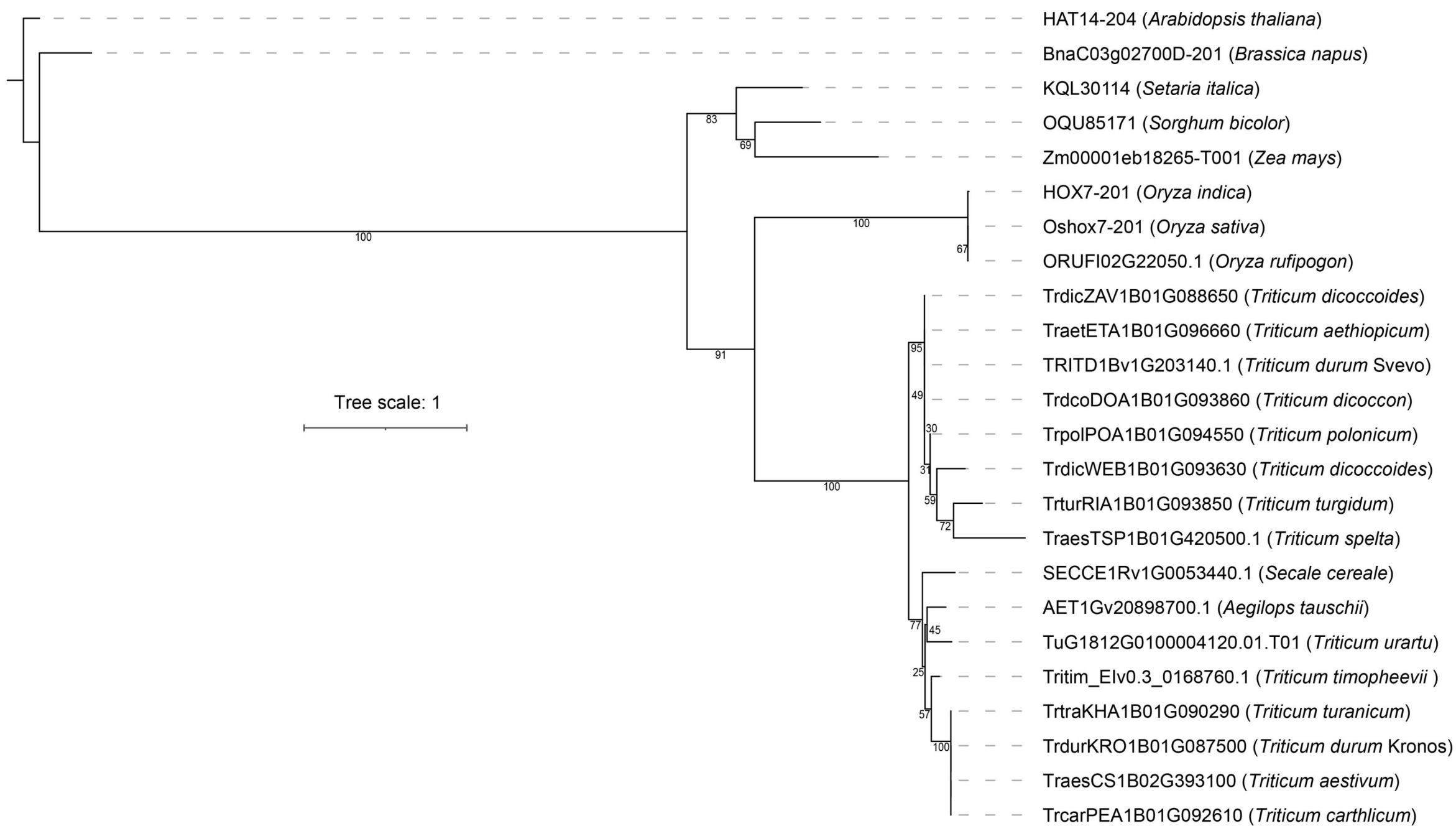

Supplementary Fig. 31 Phylogenetic analysis of HAT14-B homologs. The phylogenetic tree was constructed using protein sequences from *Arabidopsis thaliana* and selected Poaceae species using the maximum-likelihood method implemented in IQ-TREE 2. The scale represents branch length and each node represents bootstrap values from 1,000 replicates.

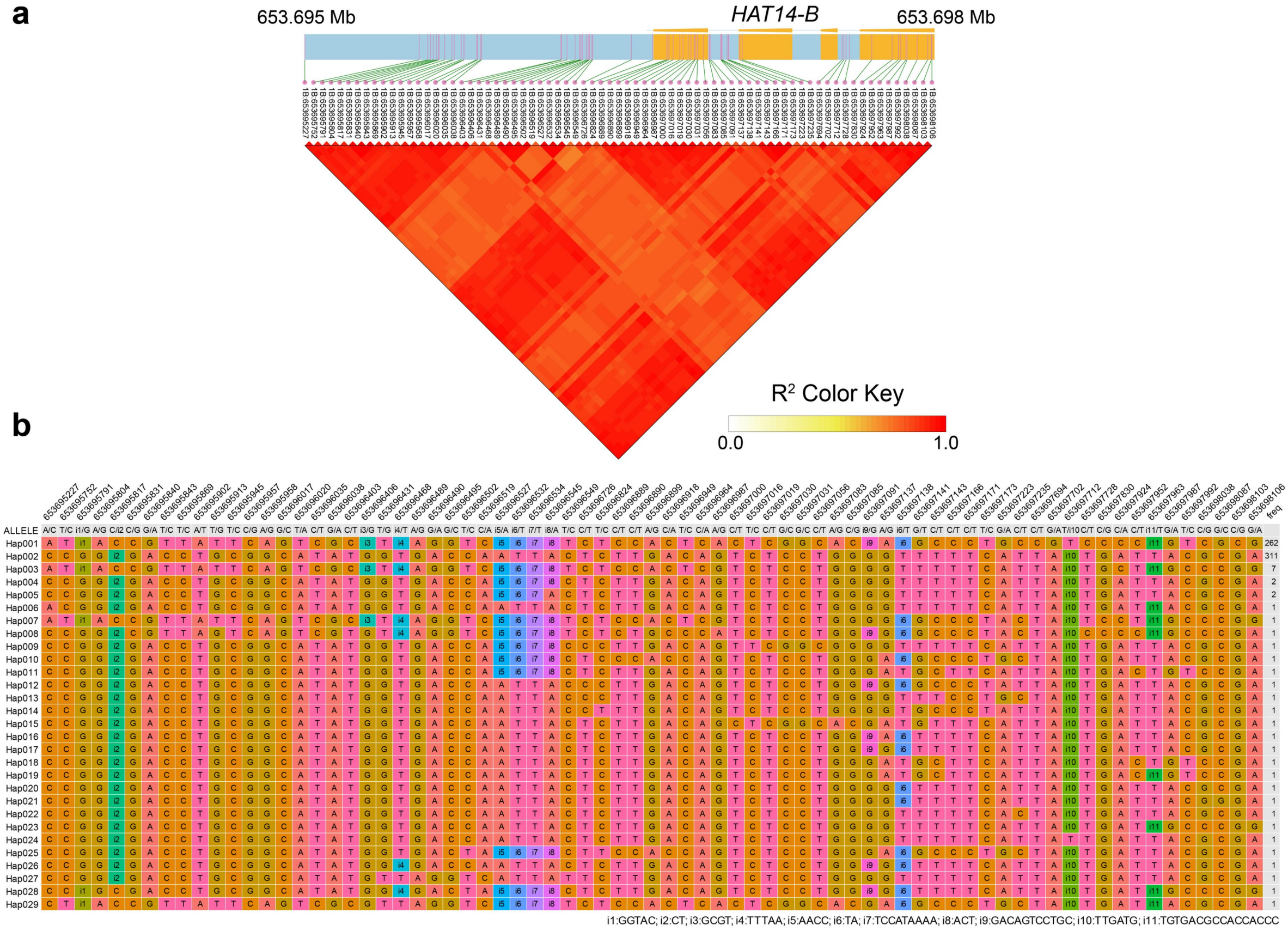

Supplementary Fig. 32 Haplotype analysis for *HAT14-B* on chromosome 1B. a, Linkage-disequilibrium block heatmap of the *HAT14-B* region and its upstream promoter region. **b,** Haplotype frequency of *HAT14-B* after removing heterozygous sites. The numbers on the right side indicate the count of individuals with the corresponding haplotype. i1–i11 below the table represent the genotype sequences of the indel variants labeled in the first line.

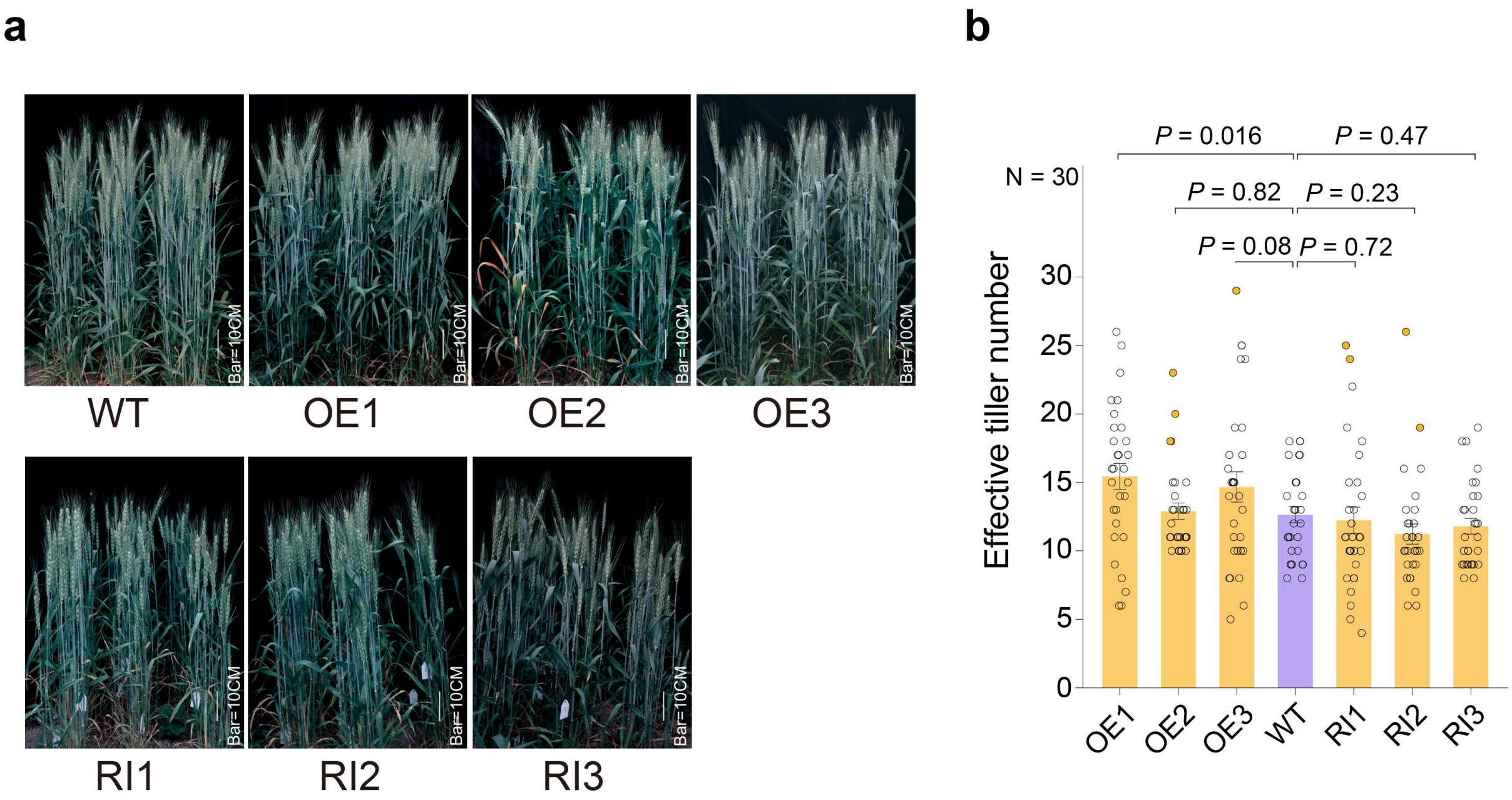

Supplementary Fig. 33 Phenotype (a) and statistical analysis (b) of effective tiller number in *HAT14-B*$^{\mathrm{Hap1}}$ overexpression and RNAi lines. Statistical significance was determined by one-way ANOVA followed by Fisher's least-significant difference multiple-comparisons tests. Error bars are mean ± s.e.m.

Chromosome 1A: 201030433

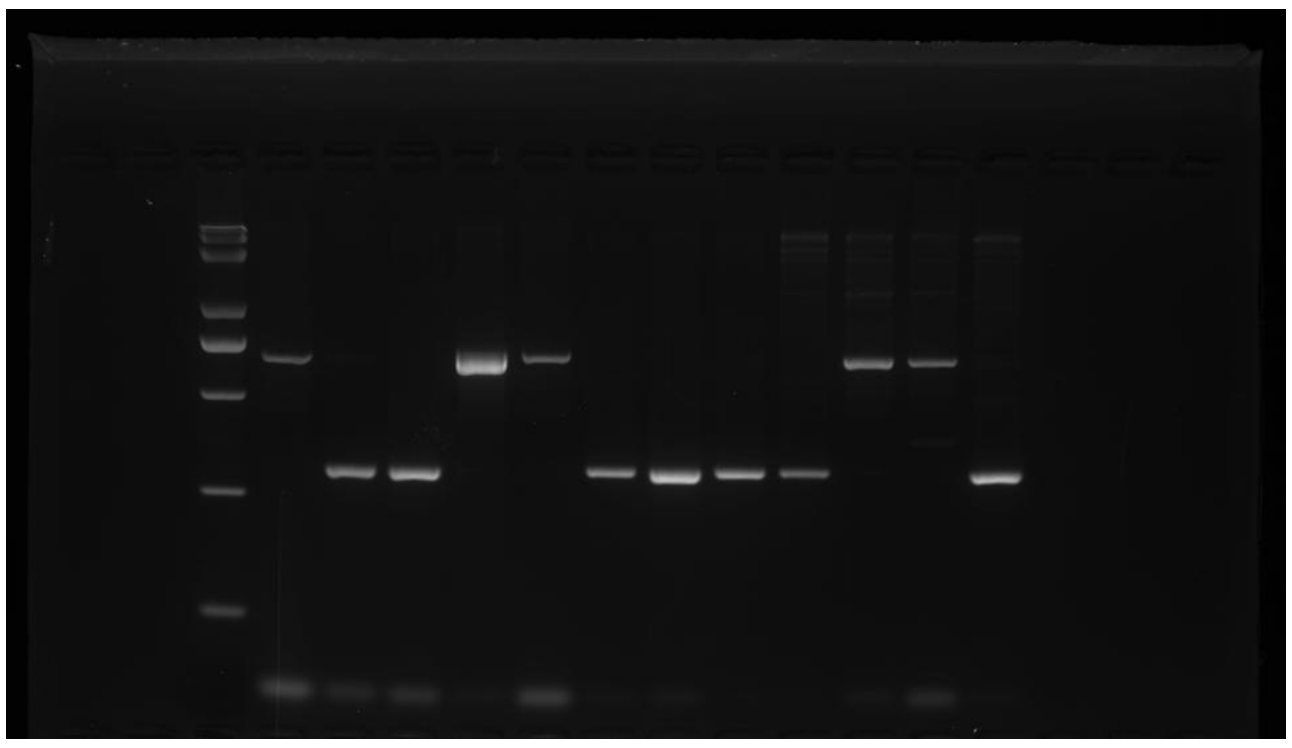

Chromosome 1A: 580435573

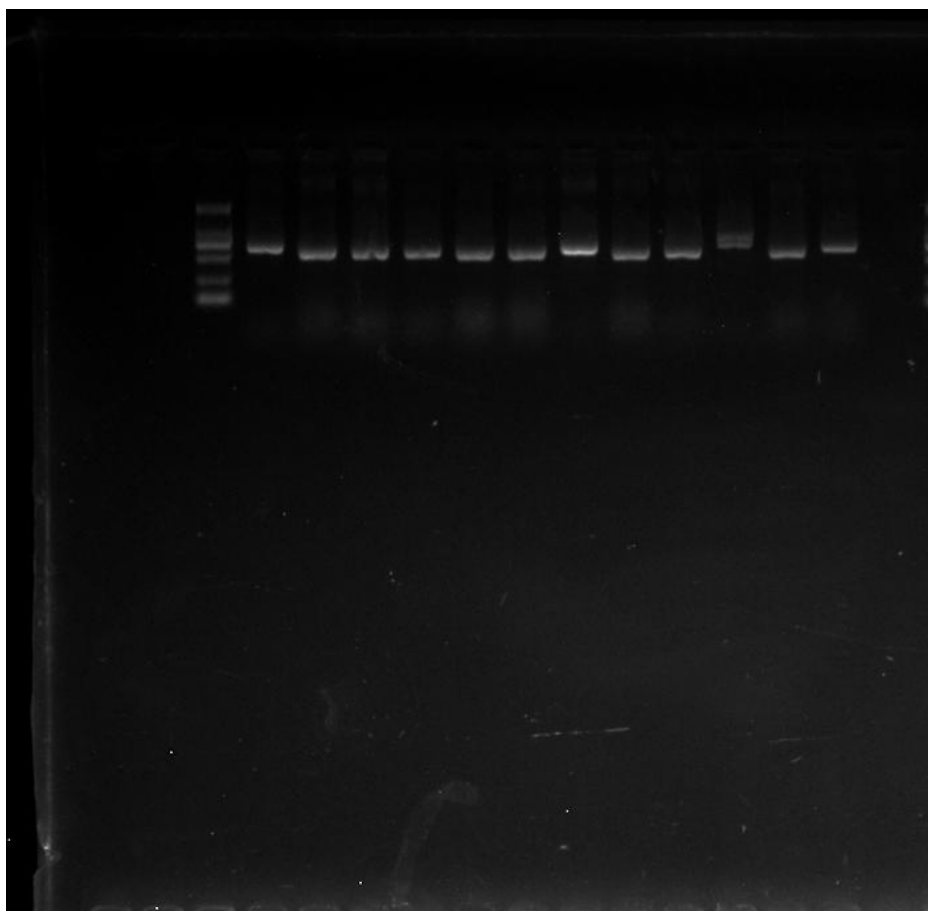

Chromosome 1B: 380495899

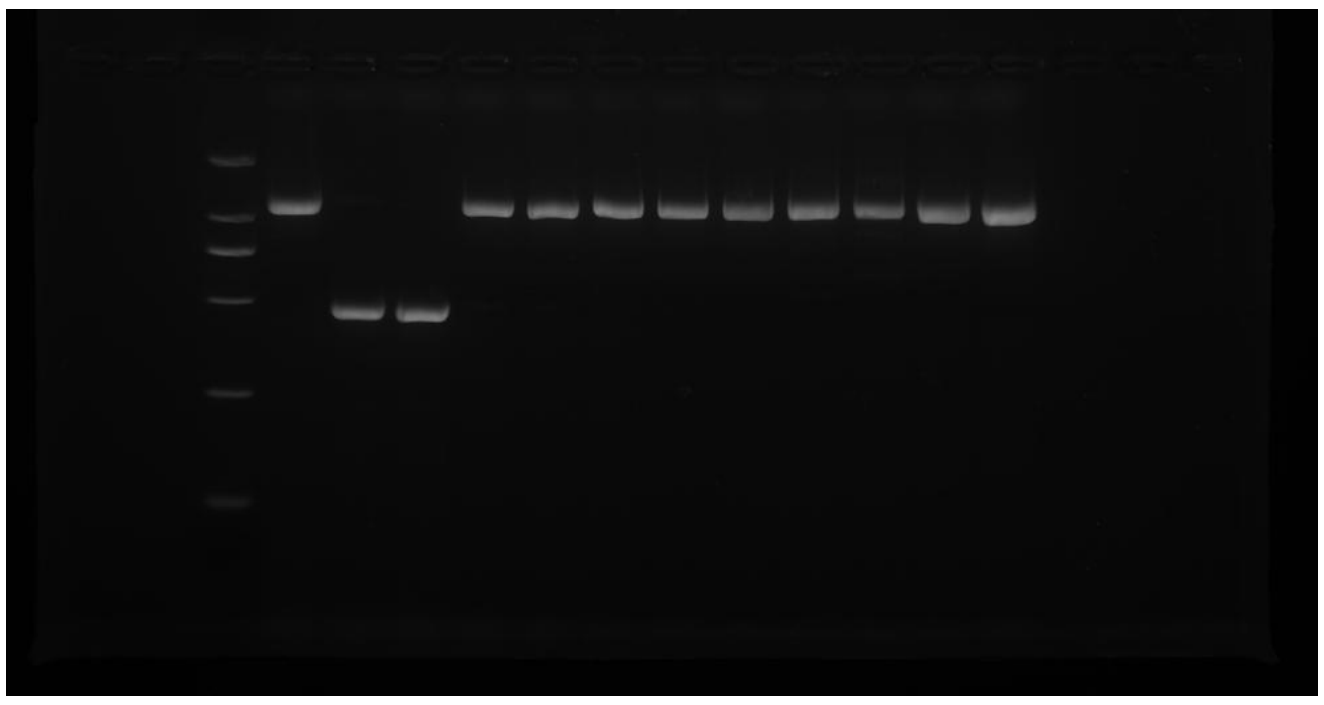

Chromosome 2A: 37124018

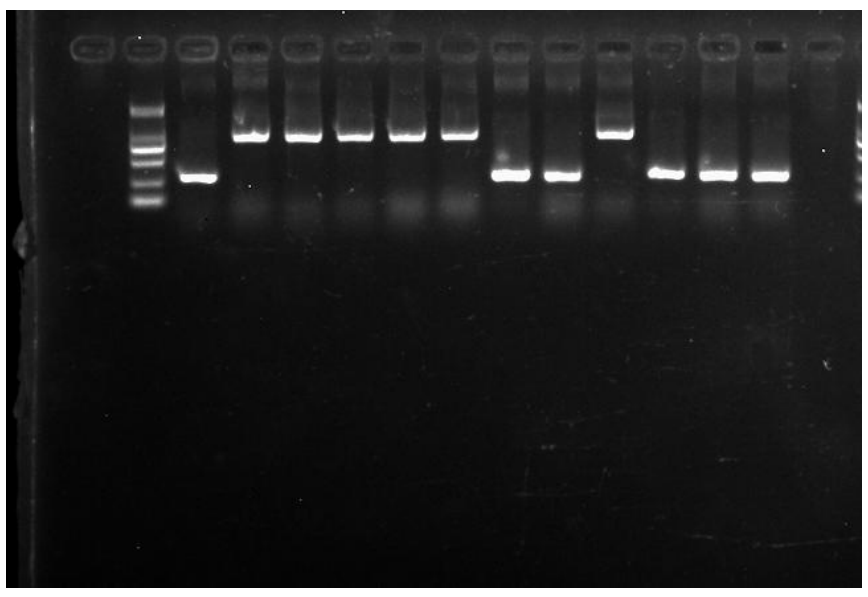

Chromosome 2A: 108960136

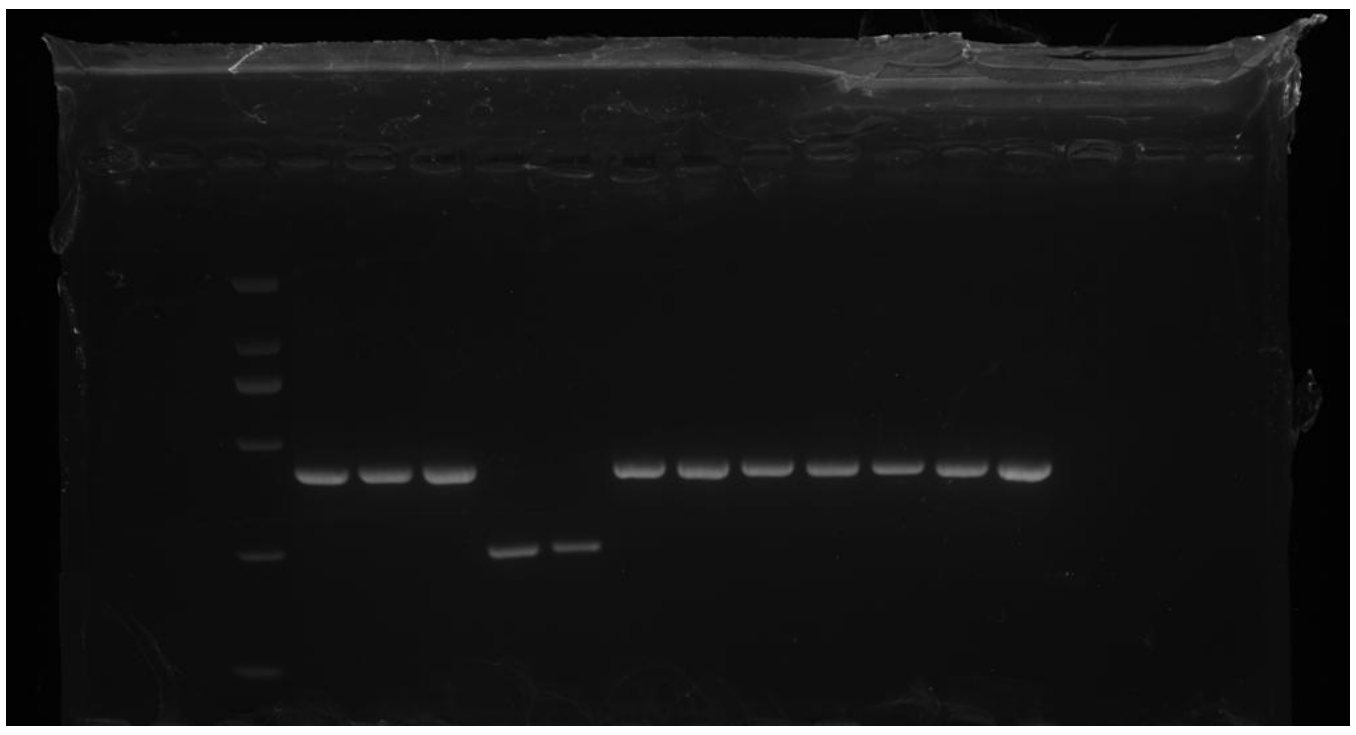

Chromosome 2A: 598706947

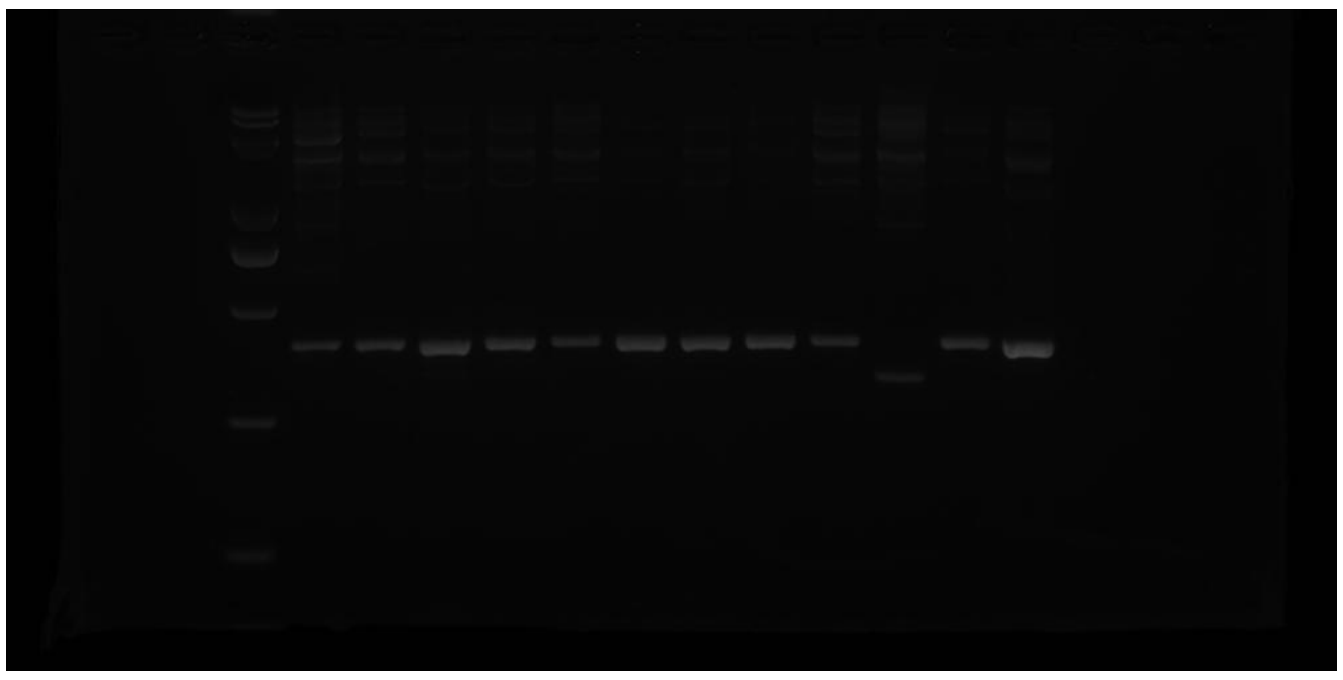

Chromosome 2A: 114928369

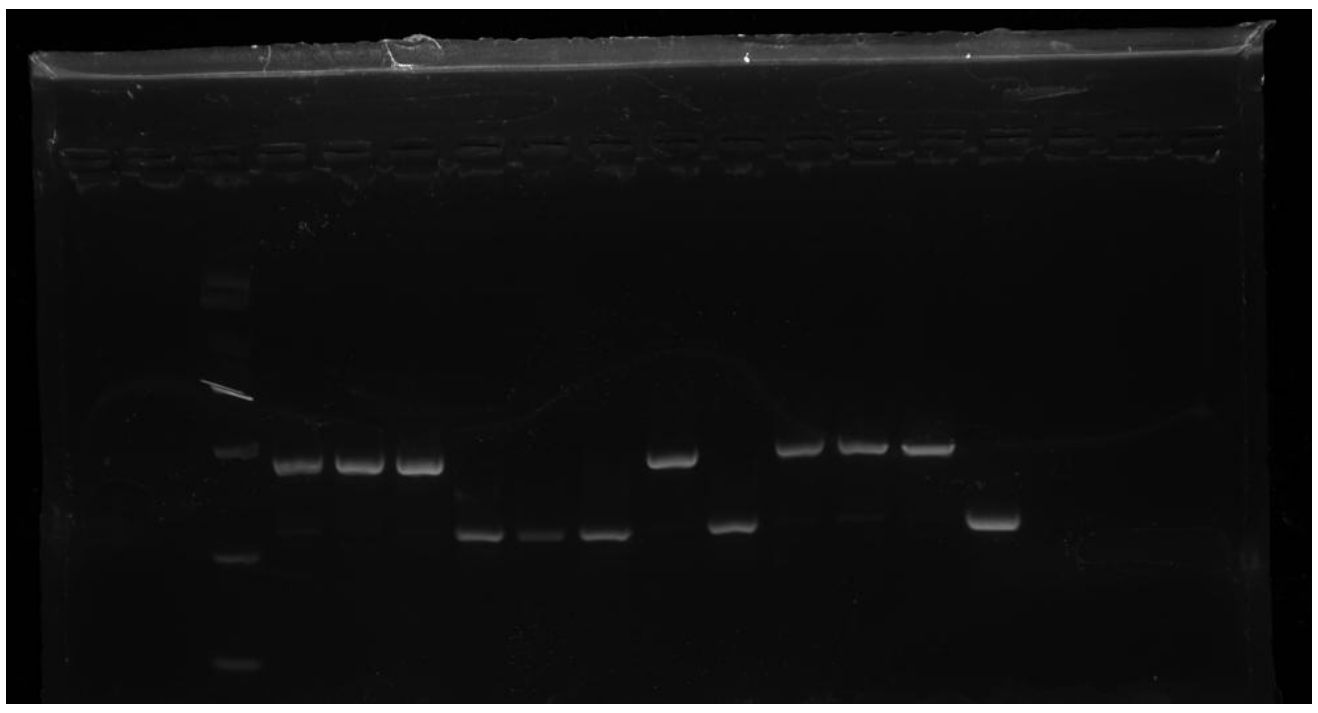

Chromosome 2B: 124436904

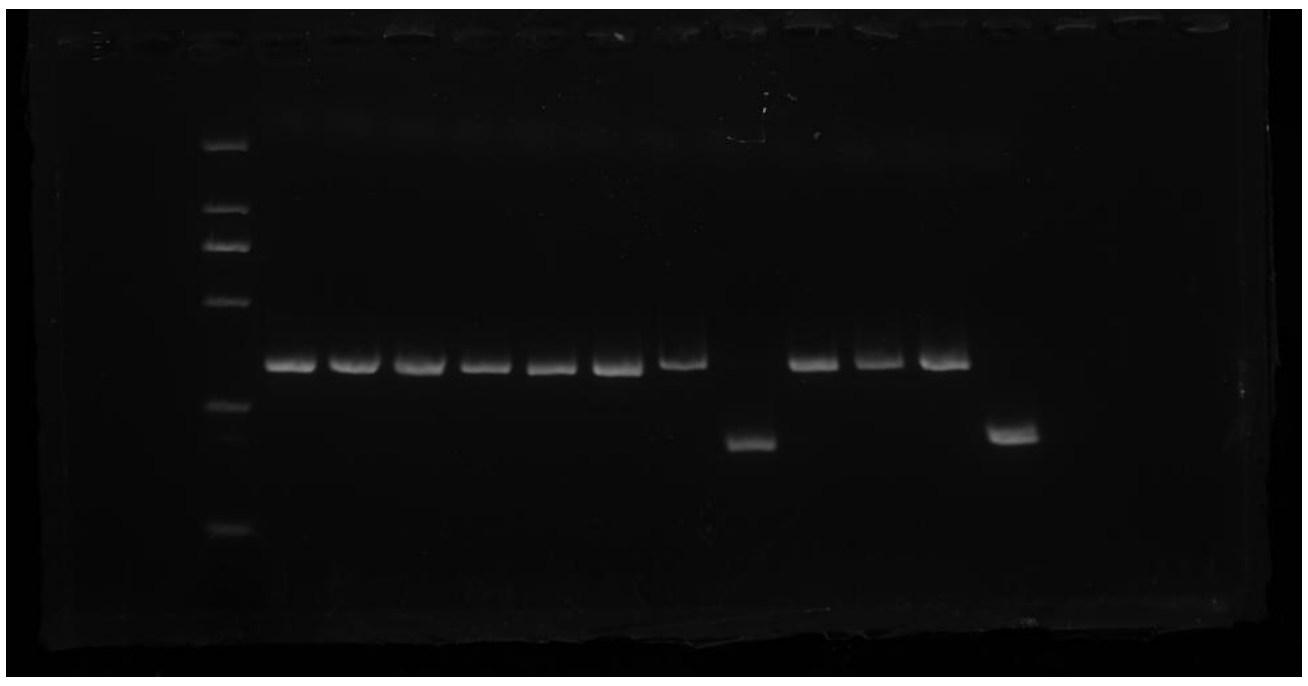

Chromosome 2B: 170368194

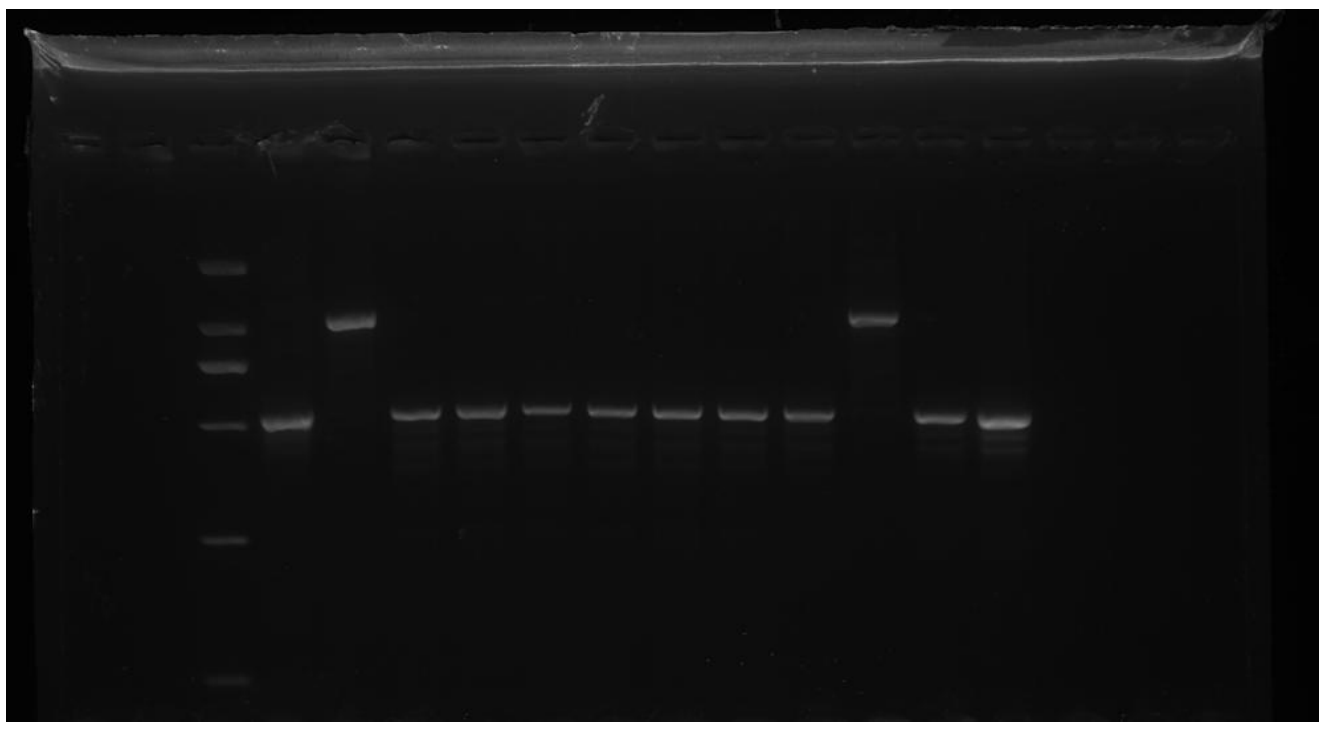

Chromosome 2B: 201119336

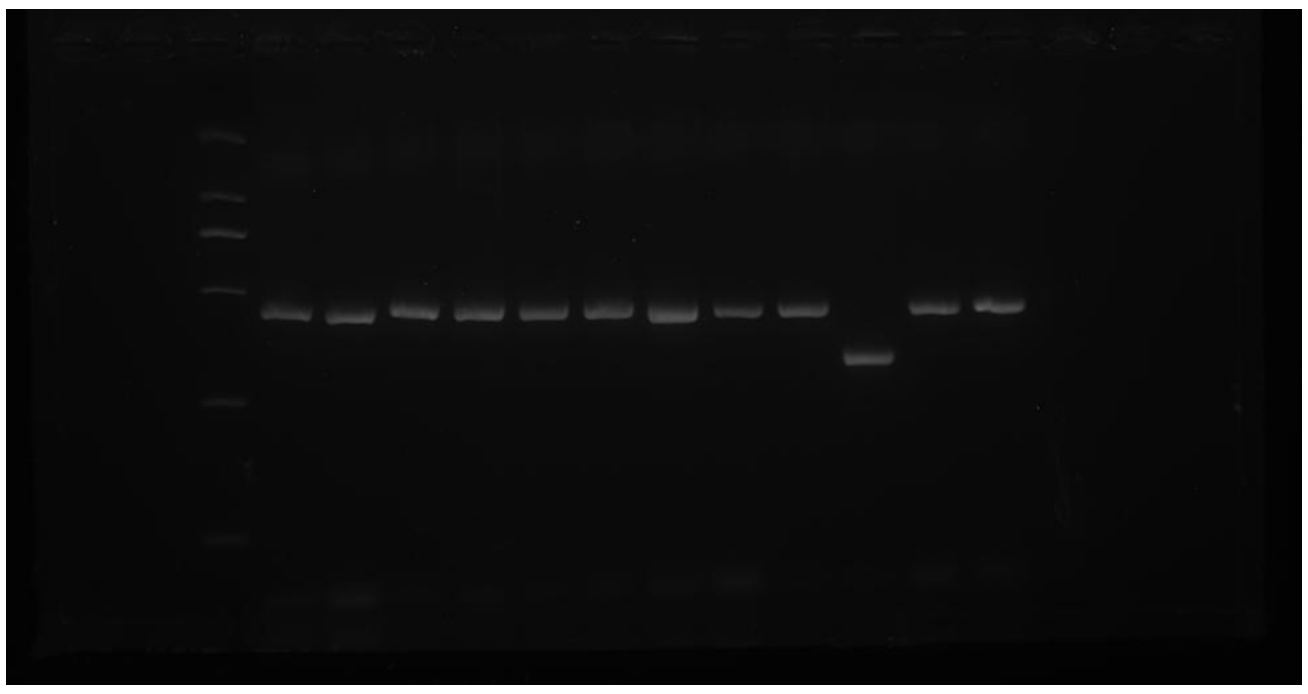

Chromosome 2B: 231267056

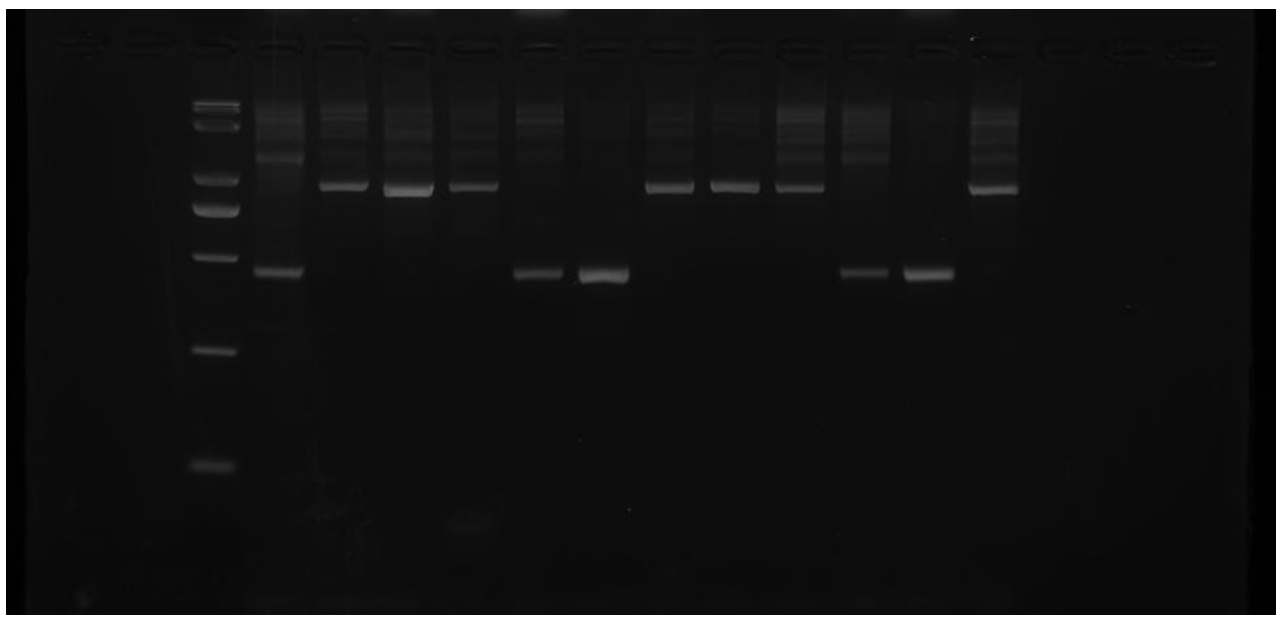

Chromosome2B: 384840397

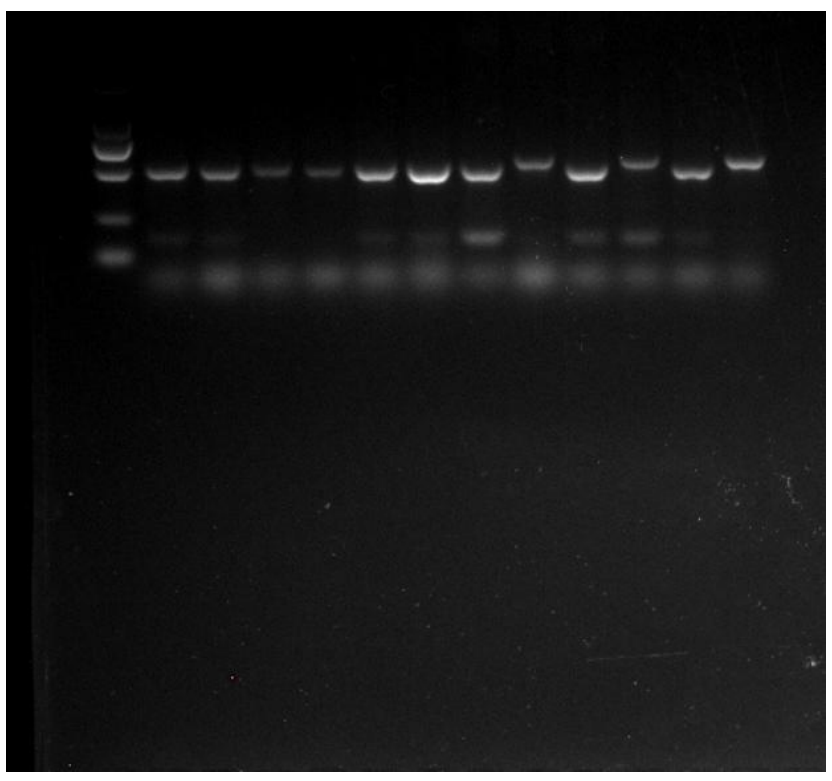

Chromosome3A: 124452063

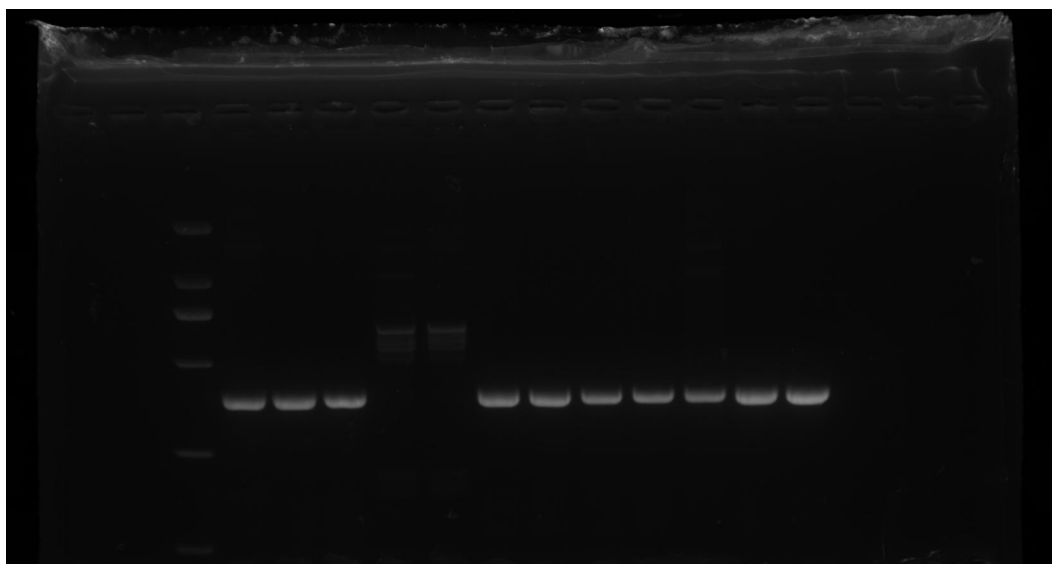

Chromosome3A: 163403436

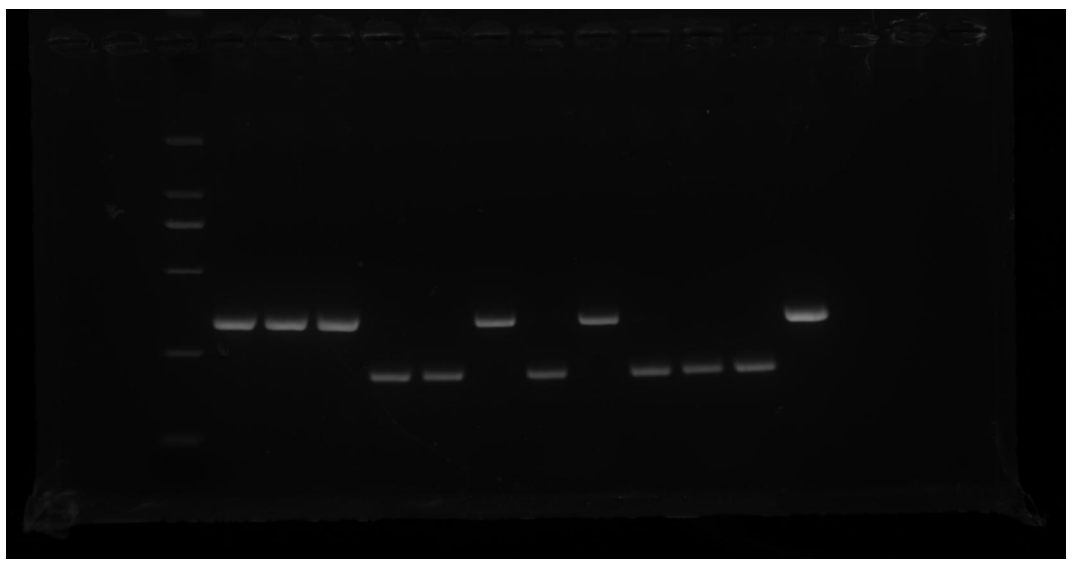

Chromosome3A: 527625200

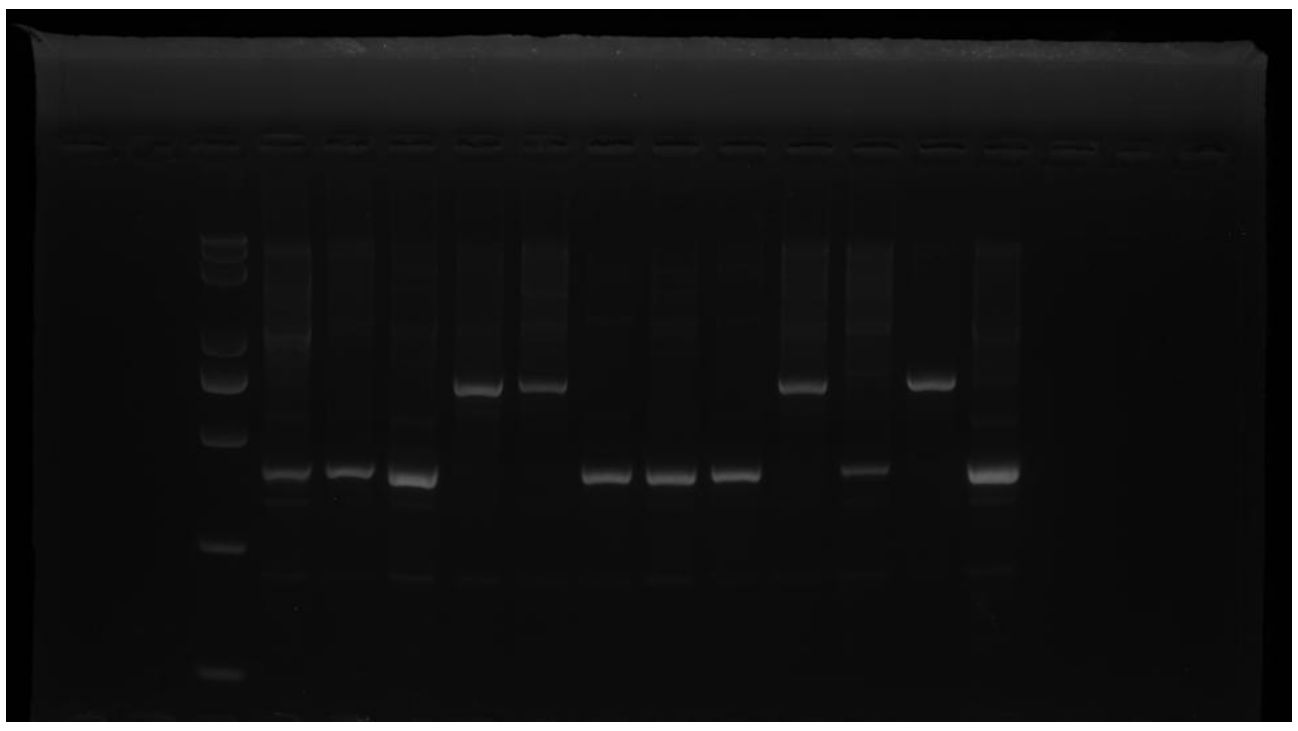

Chromosome 3B: 165059658

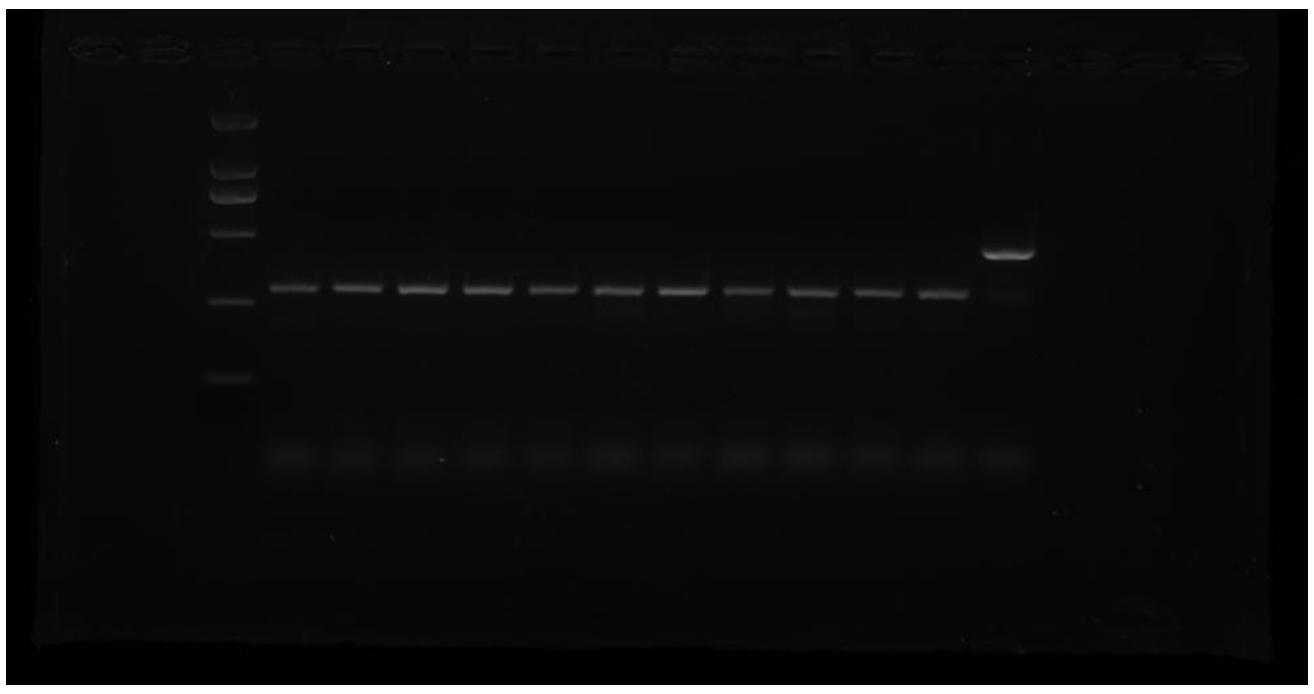

Chromosome3B: 420649836

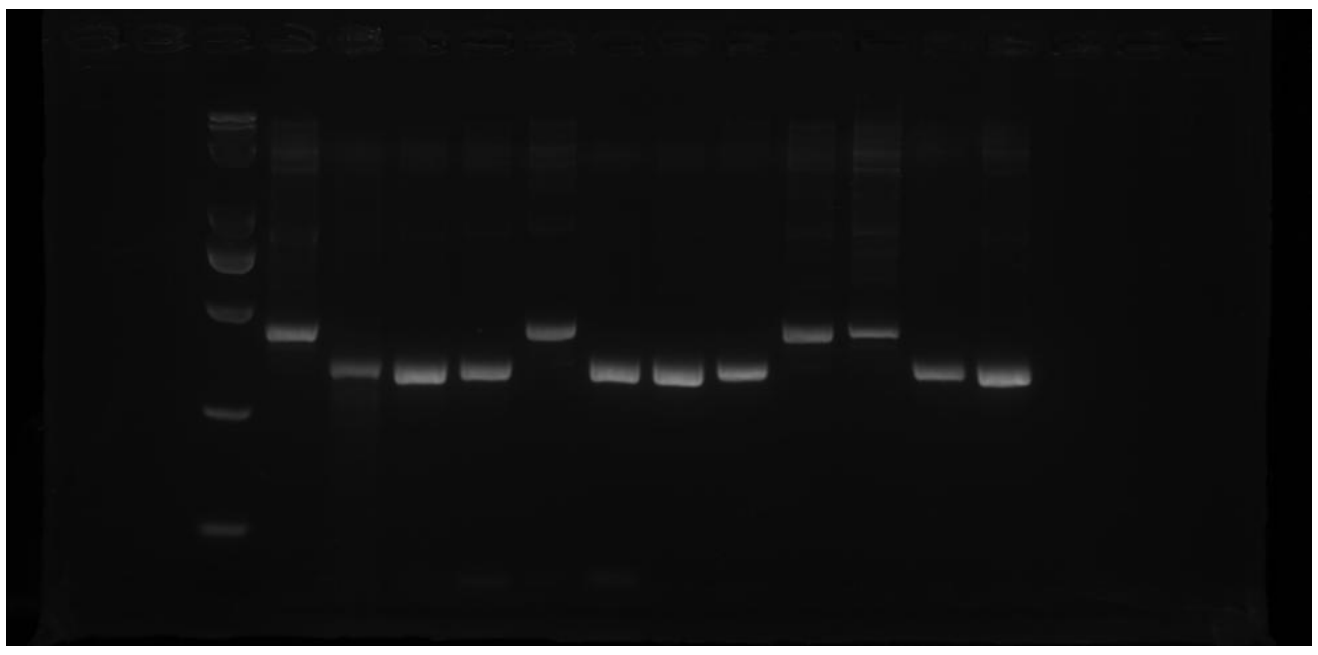

Chromosome4A: 104590705

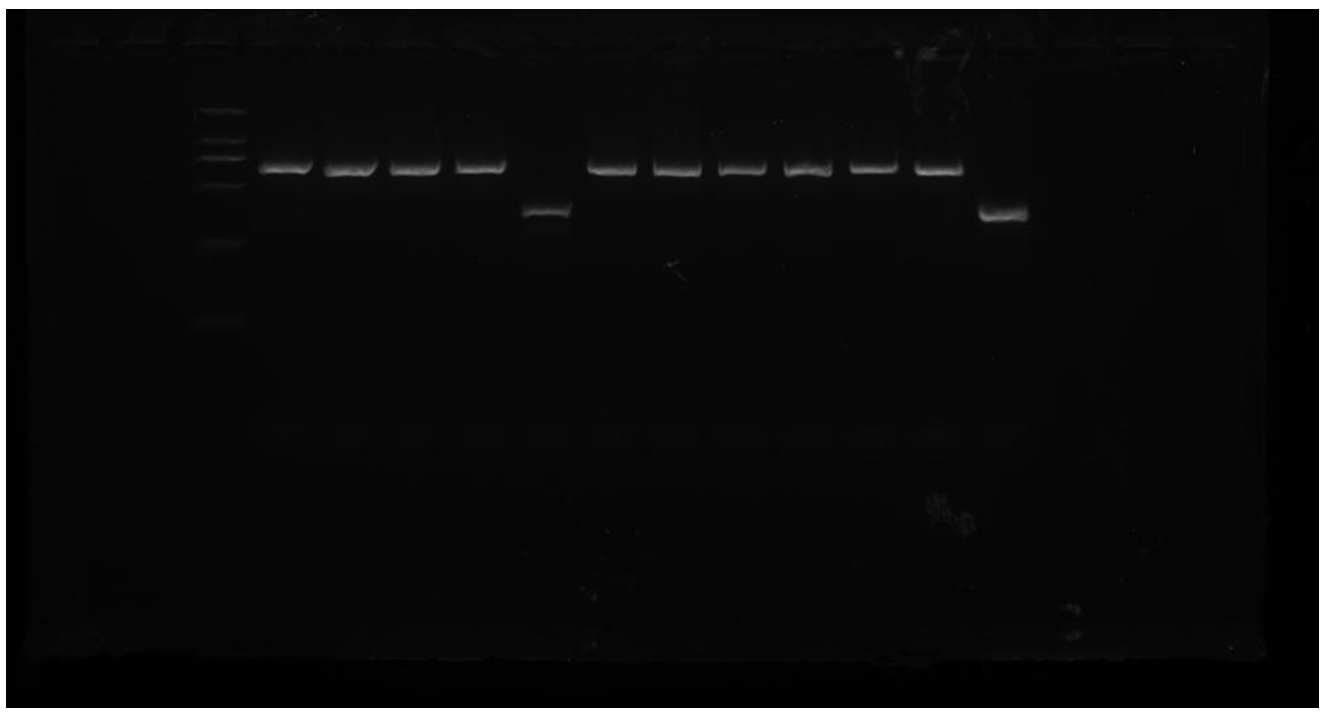

Chromosome4A: 145568799

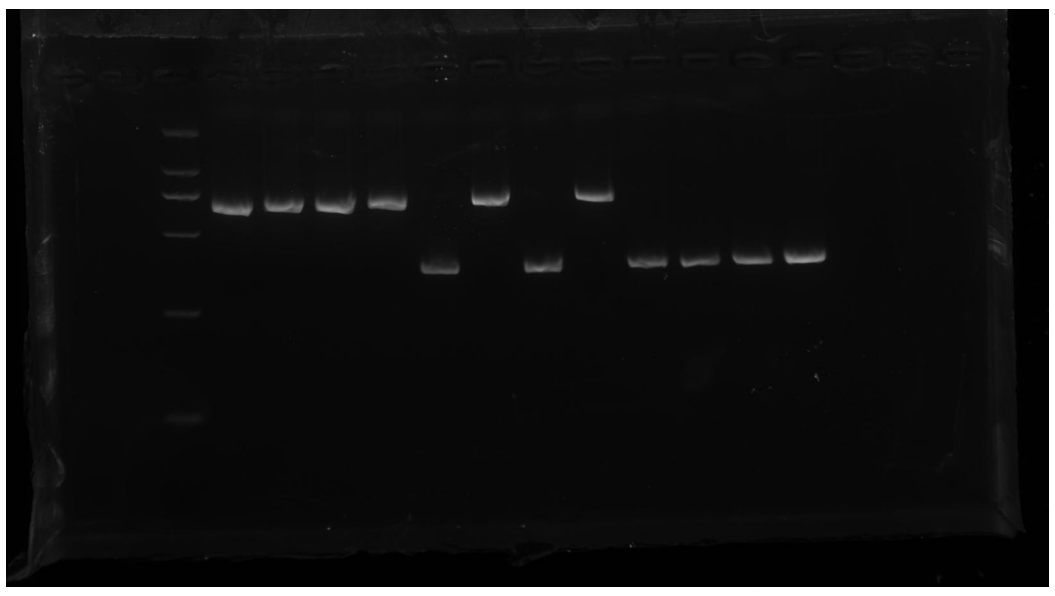

Chromosome4B: 143776260

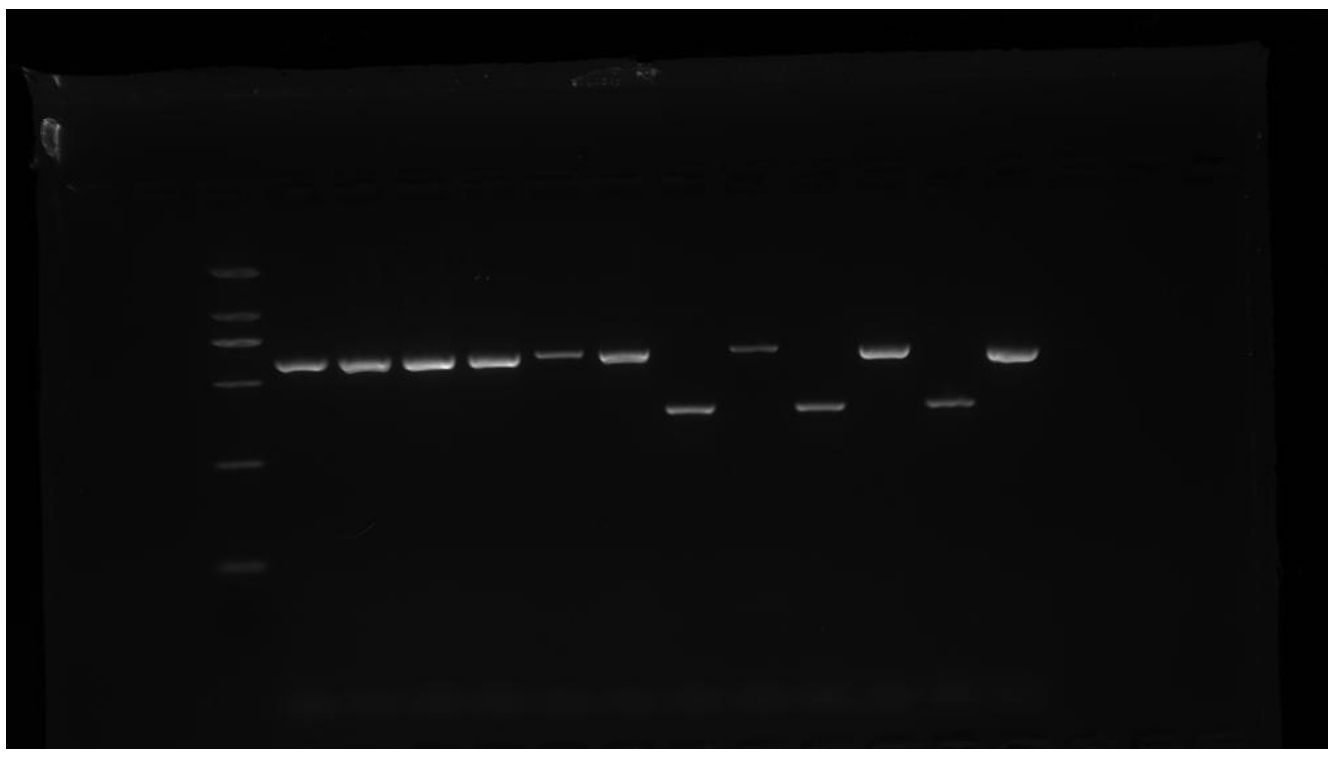

Chromosome4B: 478073041

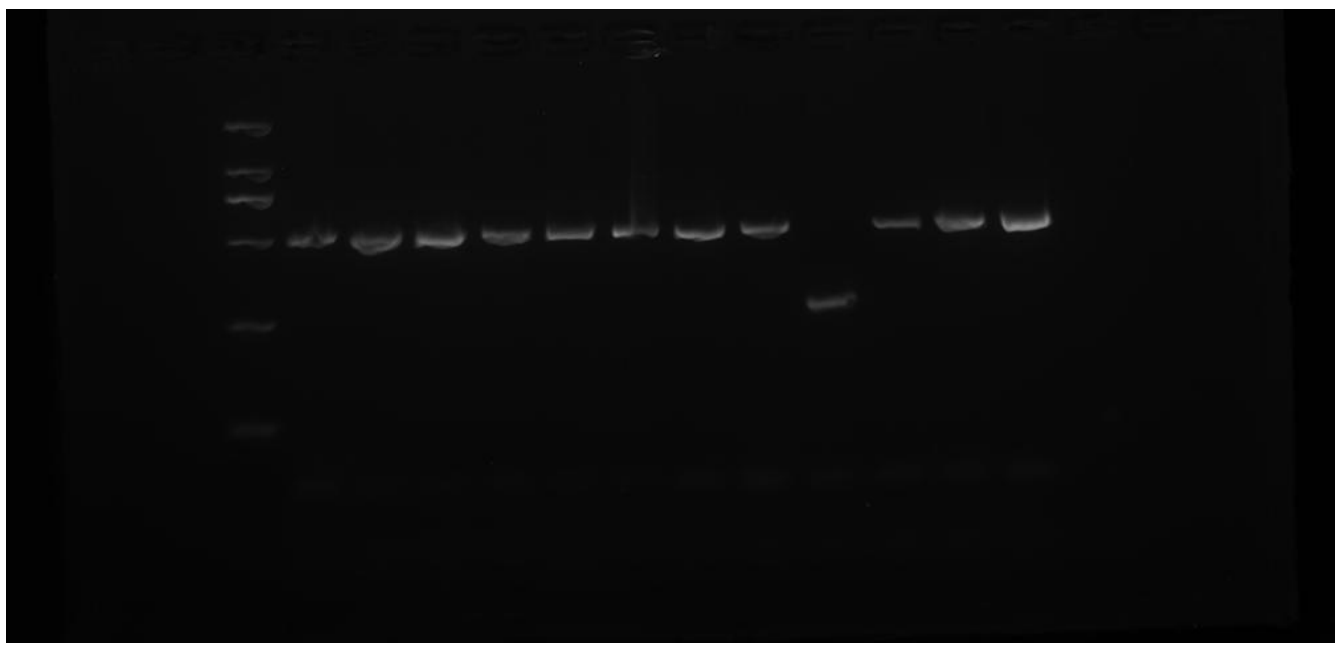

Chromosome5A: 115927540

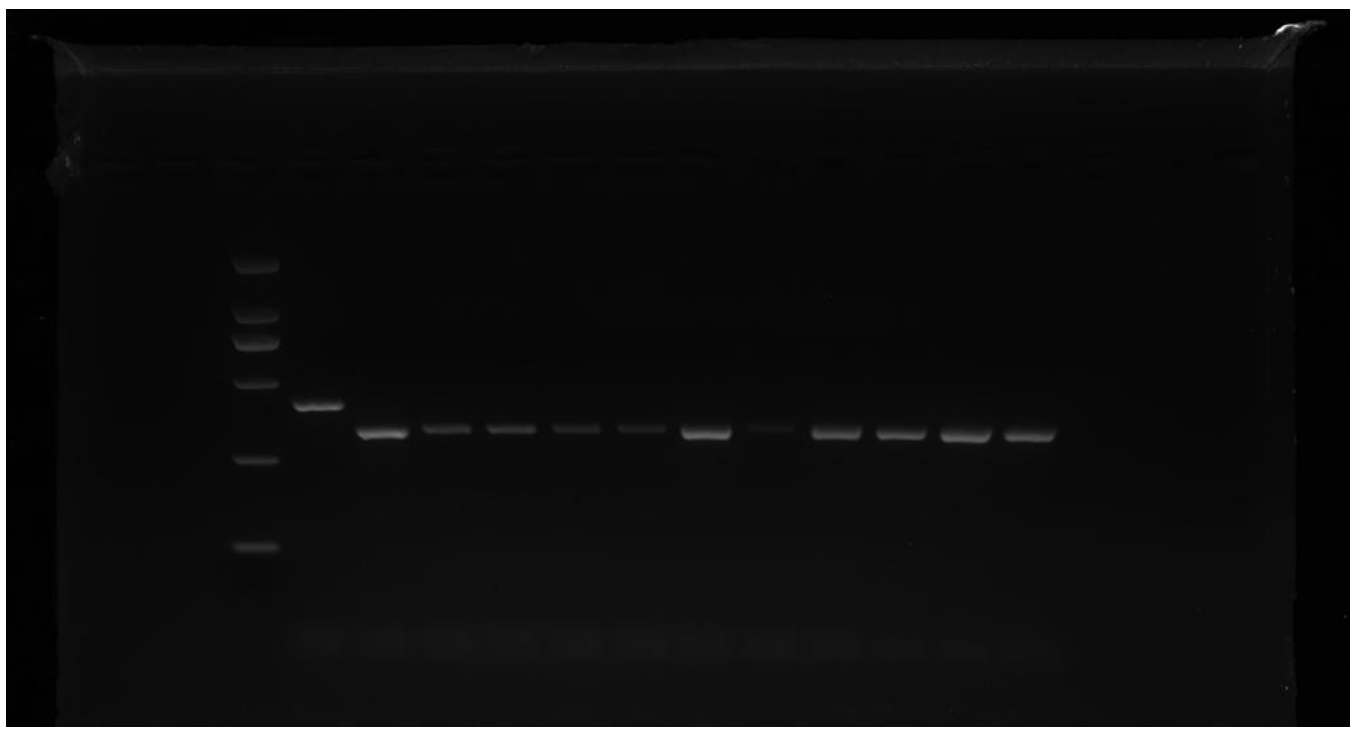

Chromosome5A: 235820973

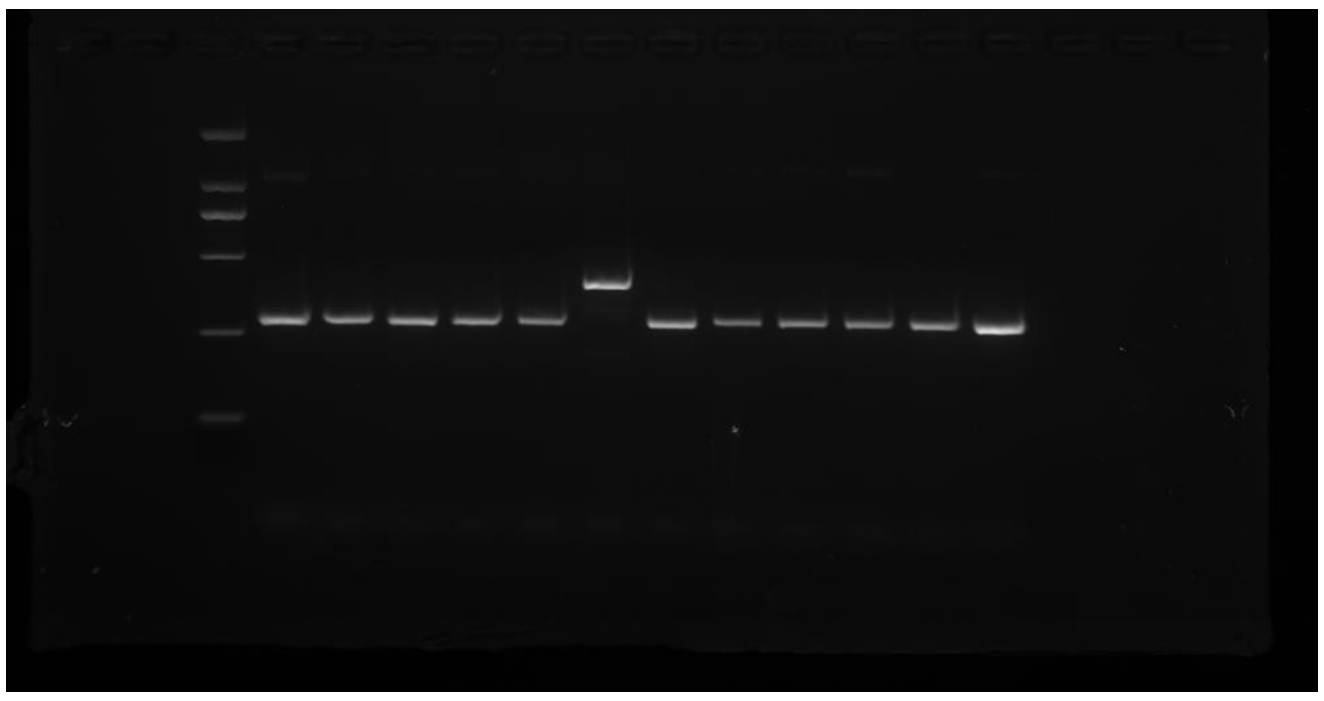

Chromosome5A: 445303589

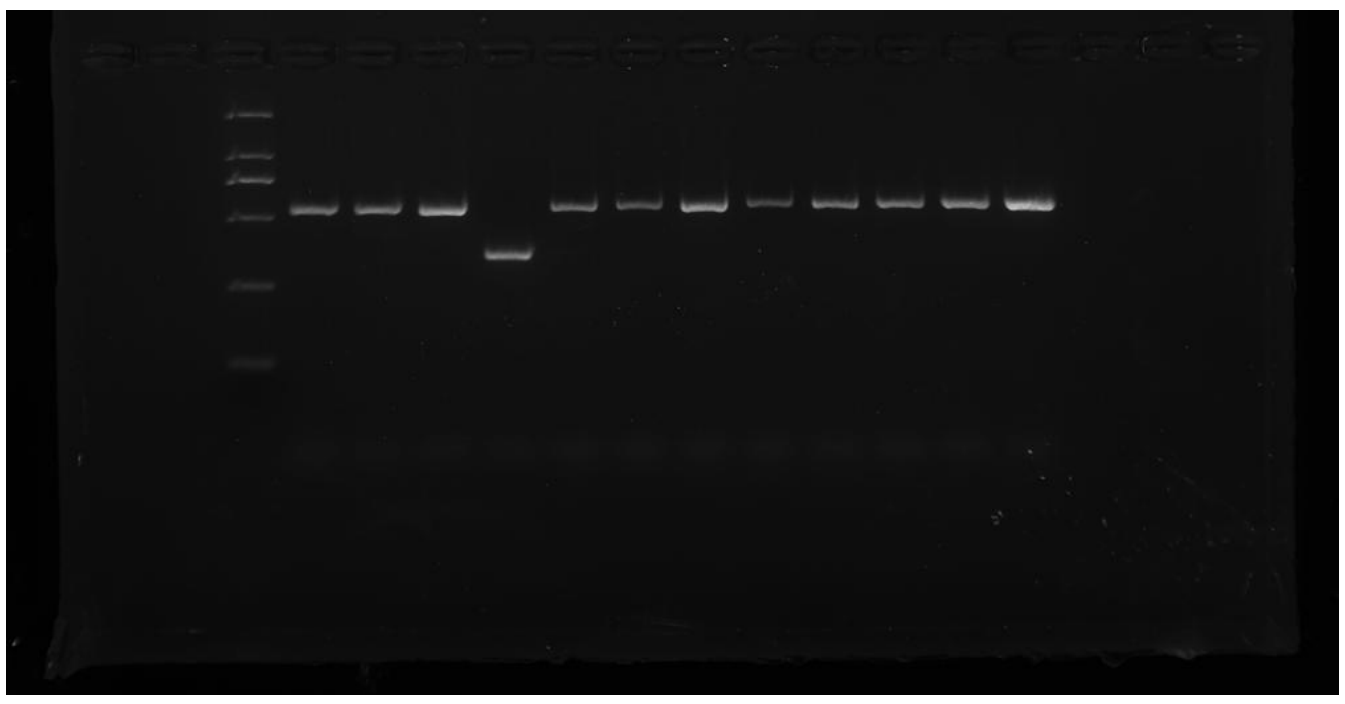

Chromosome5A: 699090173

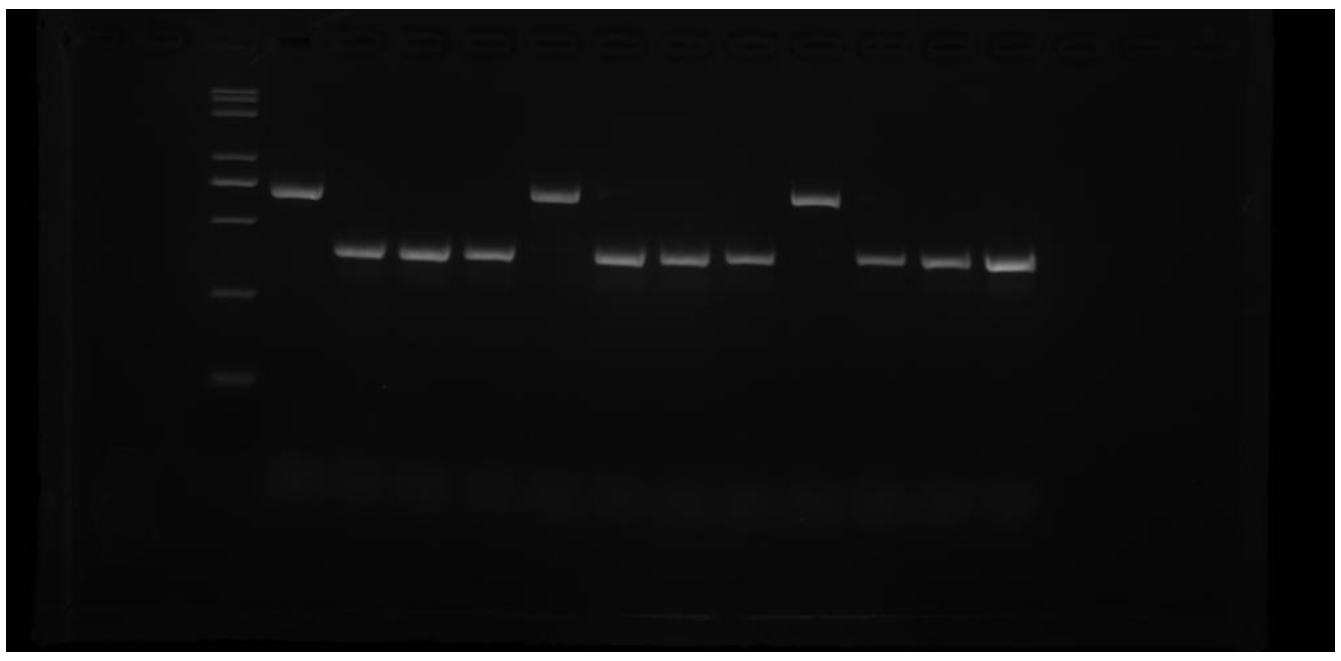

Chromosome5B: 106242451

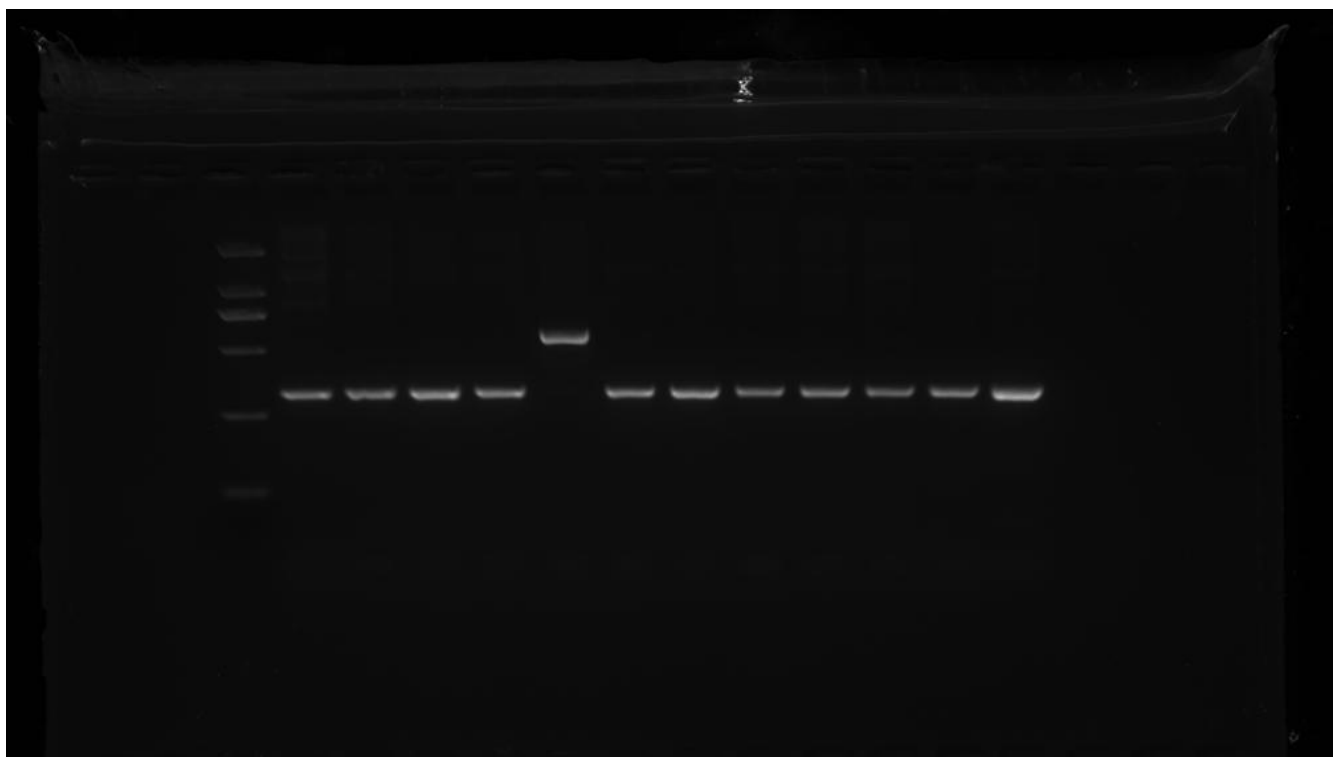

Chromosome5B: 218429433

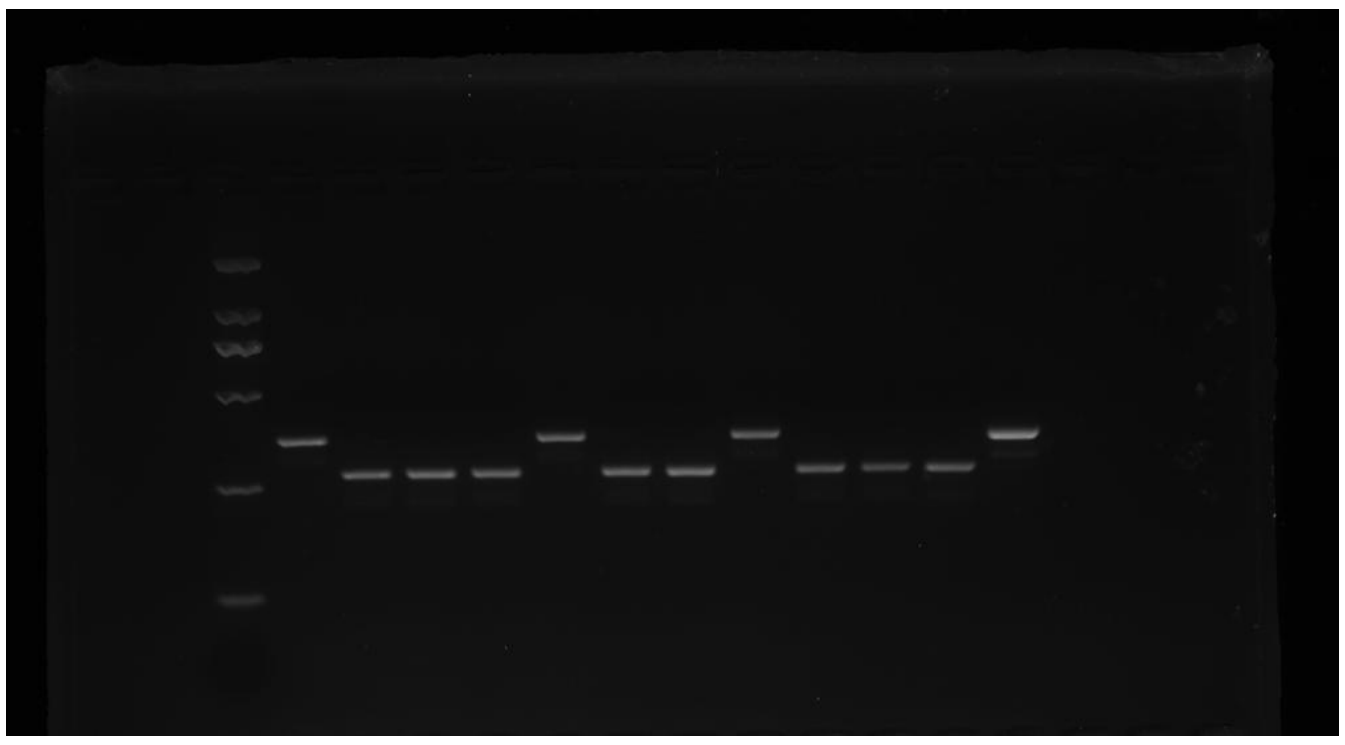

Chromosome 6A: 123065014

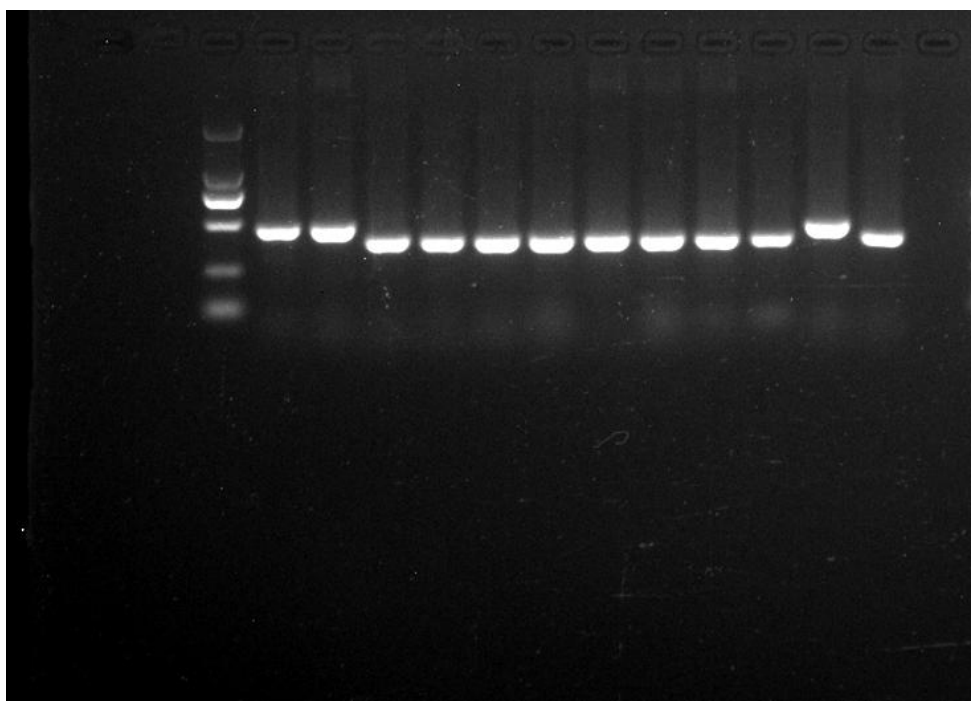

Chromosome 6A: 513512940

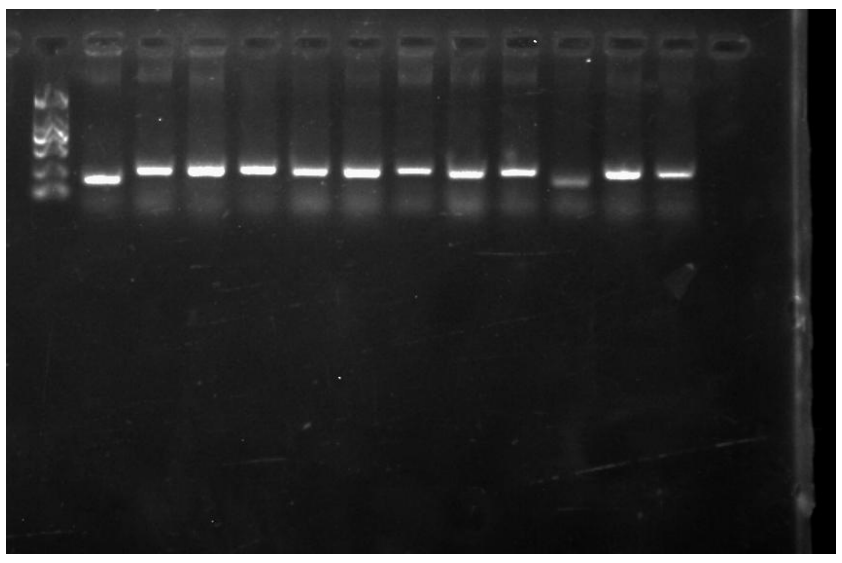

Chromosome 7A: 363833744

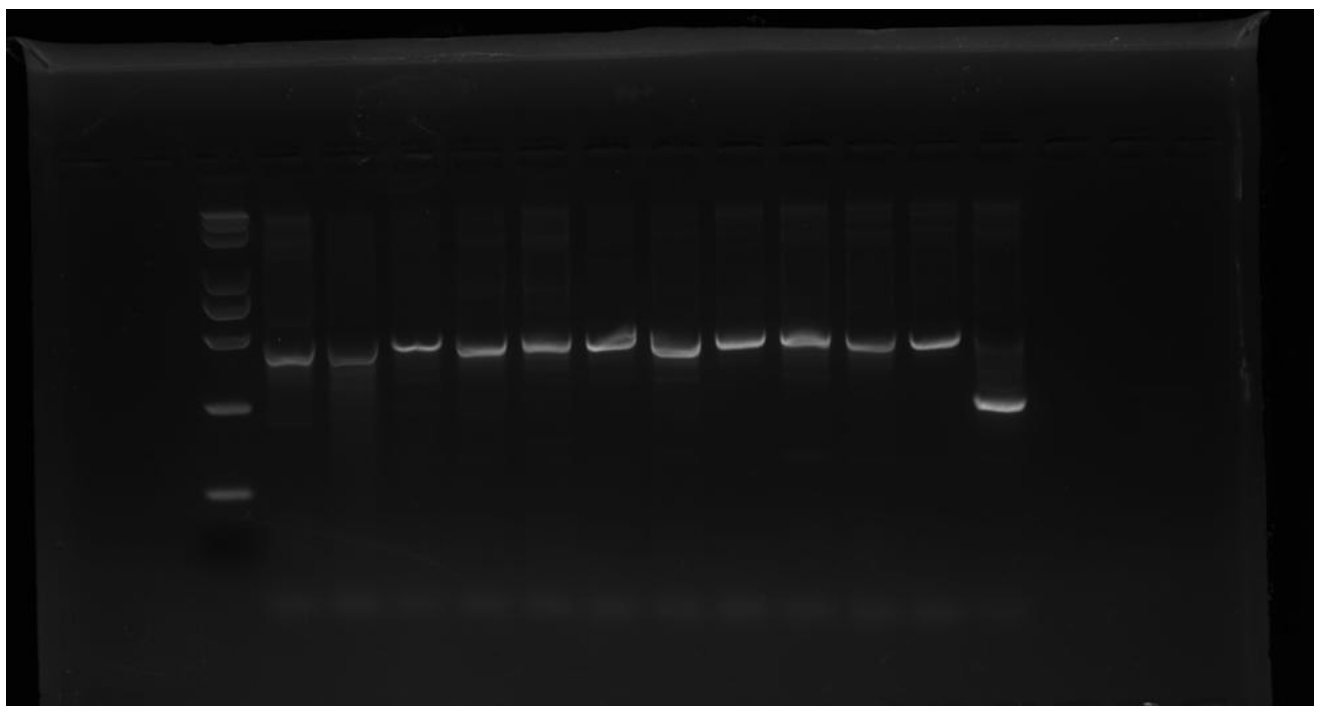

Supplementary Fig. 34 Full-length, unprocessed gel corresponding to Supplementary Fig. 19. Molecular weight markers are indicated on the left.

References

1. Liu, S. *et al.* A telomere-to-telomere genome assembly coupled with multi-omic data provides insights into the evolution of hexaploid bread wheat. *Nat. Genet.* **57**, 1008–1020 (2025).
2. Lee, T. I., Johnstone, S. E. & Young, R. A. Chromatin immunoprecipitation and microarray-based analysis of protein location. *Nat. Protoc.* **1**, 729–748 (2006).
3. Weintraub, A. S. *et al.* YY1 is a structural regulator of enhancer-promoter loops. *Cell* **171**, 1573-1588.e28 (2017).
4. De Coster, W., D'Hert, S., Schultz, D. T., Cruts, M. & Van Broeckhoven, C. NanoPack: visualizing and processing long-read sequencing data. *Bioinformatics* **34**, 2666–2669 (2018).
5. Chen, S. Ultrafast one-pass FASTQ data preprocessing, quality control, and deduplication using fastp. *Imeta* **2**, e107 (2023).
6. Liu, B. *et al.* Estimation of genomic characteristics by analyzing k-mer frequency in *de novo* genome projects. *arXiv* (2013).
7. Vurture, G. W. *et al.* GenomeScope: fast reference-free genome profiling from short reads. *Bioinformatics* **33**, 2202–2204 (2017).
8. Sun, H., Ding, J., Piednoël, M. & Schneeberger, K. *findGSE*: estimating genome size variation within human and *Arabidopsis* using k-mer frequencies. *Bioinformatics* **34**, 550–557 (2018).
9. Cheng, H., Concepcion, G. T., Feng, X., Zhang, H. & Li, H. Haplotype-resolved de novo assembly using phased assembly graphs with hifiasm. *Nat. Methods* **18**, 170–175 (2021).
10. Manni, M., Berkeley, M. R., Seppey, M., Simão, F. A. & Zdobnov, E. M. BUSCO update: Novel and streamlined workflows along with broader and deeper

phylogenetic coverage for scoring of eukaryotic, prokaryotic, and viral genomes. *Mol. Biol. Evol.* **38**, 4647–4654 (2021).

11. Zhou, C., McCarthy, S. A. & Durbin, R. YaHS: yet another Hi-C scaffolding tool. *Bioinformatics* **39**, btac808 (2023).
12. Durand, N. C. *et al.* Juicebox provides a visualization system for Hi-C contact maps with unlimited zoom. *Cell Syst.* **3**, 99–101 (2016).
13. Song, B. *et al.* AnchorWave: Sensitive alignment of genomes with high sequence diversity, extensive structural polymorphism, and whole-genome duplication. *Proc. Natl. Acad. Sci. U. S. A.* **119**, e2113075119 (2022).
14. Maccaferri, M. *et al.* Durum wheat genome highlights past domestication signatures and future improvement targets. *Nat. Genet.* **51**, 885–895 (2019).
15. Servant, N. *et al.* HiC-Pro: an optimized and flexible pipeline for Hi-C data processing. *Genome Biol.* **16**, 259 (2015).
16. Yang, G. *et al.* Combined GWAS and eGWAS reveals the genetic basis underlying drought tolerance in emmer wheat (*Triticum turgidum* L.). *New Phytol.* **242**, 2115–2131 (2024).
17. Avni, R. *et al.* Wild emmer genome architecture and diversity elucidate wheat evolution and domestication. *Science* **357**, 93–97 (2017).
18. Zhu, T. *et al.* Improved genome sequence of wild Emmer wheat Zavitan with the aid of optical maps. *G3 (Bethesda)* **9**, 619–624 (2019).
19. Chen, M. *et al.* Chromosome-level genome assembly of the Durum wheat cultivar Langdon. *Sci. Data* **12**, 1372 (2025).
20. Emms, D. M. & Kelly, S. OrthoFinder: phylogenetic orthology inference for comparative genomics. *Genome Biol.* **20**, 238 (2019).
21. Mendes, F. K., Vanderpool, D., Fulton, B. & Hahn, M. W. CAFE 5 models

variation in evolutionary rates among gene families. *Bioinformatics* **36**, 5516–5518 (2021).

22. Green, R. E. *et al.* A draft sequence of the Neandertal genome. *Science* **328**, 710–722 (2010).
23. Martin, S. H., Davey, J. W. & Jiggins, C. D. Evaluating the use of ABBA-BABA statistics to locate introgressed loci. *Mol. Biol. Evol.* **32**, 244–257 (2015).
24. Zhou, Y. *et al. Triticum* population sequencing provides insights into wheat adaptation. *Nat. Genet.* **52**, 1412–1422 (2020).
25. Hunter, J. D. Matplotlib: A 2D Graphics Environment. *Comput. Sci. Eng.* **9**, 90–95 (2007).
26. Jones, P. *et al.* InterProScan 5: genome-scale protein function classification. *Bioinformatics* **30**, 1236–1240 (2014).
27. Han, F., Lamb, J. C. & Birchler, J. A. High frequency of centromere inactivation resulting in stable dicentric chromosomes of maize. *Proc. Natl. Acad. Sci. U. S. A.* **103**, 3238–3243 (2006).
28. Tang, Z., Yang, Z. & Fu, S. Oligonucleotides replacing the roles of repetitive sequences pAs1, pSc119.2, pTa-535, pTa71, CCS1, and pAWRC.1 for FISH analysis. *J. Appl. Genet.* **55**, 313–318 (2014).
29. Yu, Z., Li, G., Zheng, Z., Wang, H. & Yang, Z. Characterization of new wheat-*Thinopyrum intermedium* derivative lines with superior genes for stripe rust and powdery mildew resistance. *Plants* **13**, 2333 (2024).
30. Lang, T. *et al.* Precise identification of wheat - *Thinopyrum intermedium* translocation chromosomes carrying resistance to wheat stripe rust in line Z4 and its derived progenies. *Genome* **61**, 177–185 (2018).
31. Jiang, W. *et al.* A universal karyotypic system for hexaploid and diploid *Avena*

species brings oat cytogenetics into the genomics era. *BMC Plant Biol.* **21**, 213 (2021).

32. Komuro, S., Endo, R., Shikata, K. & Kato, A. Genomic and chromosomal distribution patterns of various repeated DNA sequences in wheat revealed by a fluorescence in situ hybridization procedure. *Genome* **56**, 131–137 (2013).
33. Vasimuddin, M., Misra, S., Li, H. & Aluru, S. Efficient architecture-aware acceleration of BWA-MEM for multicore systems. in *2019 IEEE International Parallel and Distributed Processing Symposium (IPDPS)* 314–324 (IEEE, 2019).
34. Stamatakis, A. RAxML version 8: a tool for phylogenetic analysis and post-analysis of large phylogenies. *Bioinformatics* **30**, 1312–1313 (2014).
35. Kruskal, J. B. On the shortest spanning subtree of a graph and the traveling salesman problem. *Proc. Amer. Math. Soc.* **7**, 48–50 (1956).
36. Karyotis, V., Tsitseklis, K., Sotiropoulos, K. & Papavassiliou, S. Big data clustering via community detection and hyperbolic network embedding in IoT applications. *Sensors (Basel)* **18**, 1205 (2018).
37. Li, M., Li, D., Tang, Y., Wu, F. & Wang, J. CytoCluster: A cytoscape plugin for cluster analysis and visualization of biological networks. *Int. J. Mol. Sci.* **18**, 1880 (2017).
38. Shannon, P. *et al.* Cytoscape: a software environment for integrated models of biomolecular interaction networks. *Genome Res.* **13**, 2498–2504 (2003).
39. Kucera, M., Isserlin, R., Arkhangorodsky, A. & Bader, G. D. AutoAnnotate: A Cytoscape app for summarizing networks with semantic annotations. *F1000Res.* **5**, 1717 (2016).
40. He, F. *et al.* Exome sequencing highlights the role of wild-relative introgression in shaping the adaptive landscape of the wheat genome. *Nat. Genet.* **51**, 896–

904 (2019).

41. Cheng, H. *et al.* Frequent intra- and inter-species introgression shapes the landscape of genetic variation in bread wheat. *Genome Biol.* **20**, 136 (2019).
