# Supplementary material for: A pangenome of tetraploid wheat reveals the genetic architecture underlying domestication and genomic diversity for breeding
Source: Nat Genet. 2026 Jul 22;58(8):2027–39. doi: 10.1038/s41588-026-02678-9 (PMC13447107; doi:10.1038/s41588-026-02678-9)
Supplement: Supplementary file 4 [file 41588_2026_2678_MOESM2_ESM.pdf]

nature portfolio

Corresponding author(s): Shisheng Chen, Qing Sang, Xing Wang Deng, Hude Mao, Xiaojun Nie, Baoxing Song

Last updated by author(s): May 12, 2026

Reporting Summary

Nature Portfolio wishes to improve the reproducibility of the work that we publish. This form provides structure for consistency and transparency in reporting. For further information on Nature Portfolio policies, see our Editorial Policies and the Editorial Policy Checklist.

Statistics

For all statistical analyses, confirm that the following items are present in the figure legend, table legend, main text, or Methods section.

n/a	Confirmed	
☐	☒	The exact sample size (*n*) for each experimental group/condition, given as a discrete number and unit of measurement
☐	☒	A statement on whether measurements were taken from distinct samples or whether the same sample was measured repeatedly
☐	☒	The statistical test(s) used AND whether they are one- or two-sided<br>*Only common tests should be described solely by name; describe more complex techniques in the Methods section.*
☒	☐	A description of all covariates tested
☐	☒	A description of any assumptions or corrections, such as tests of normality and adjustment for multiple comparisons
☐	☒	A full description of the statistical parameters including central tendency (e.g. means) or other basic estimates (e.g. regression coefficient) AND variation (e.g. standard deviation) or associated estimates of uncertainty (e.g. confidence intervals)
☐	☒	For null hypothesis testing, the test statistic (e.g. *F*, *t*, *r*) with confidence intervals, effect sizes, degrees of freedom and *P* value noted<br>*Give P values as exact values whenever suitable.*
☒	☐	For Bayesian analysis, information on the choice of priors and Markov chain Monte Carlo settings
☒	☐	For hierarchical and complex designs, identification of the appropriate level for tests and full reporting of outcomes
☐	☒	Estimates of effect sizes (e.g. Cohen's *d*, Pearson's *r*), indicating how they were calculated

Our web collection on statistics for biologists contains articles on many of the points above.

Software and code

Policy information about availability of computer code

Data collection	No software used for data collection.
Data analysis	Genome assembly: SMRT Link v13.1, dorado v0.4.1, Fastp v0.23.2, NanoFilt v2.8.0, Kmerfreq (no versions, https://github.com/fanagislab/kmerfreq), GCE v1.0.2,Genomescope v1.0, findGSE v0.1.0, hifiasm v0.22.0-r689, minimap2 v2.24, YaHS v1.2a.1, Juicebox v1.11.08, HiC-Pro v3.1.0, BUSCO v5.4.6, Merqury v1.3, LTR_retriever v2.9.0, Verkko v2.0, NextDenovo v2.5.2, RagTag v2.1.0, AnchorWave v1.2.6, NextPolish v1.4.1, Bowtie2 v2.5.1, SAMtools v1.10, MACS3 v3.0.1, Deeptools v3.5.5, StainedGlass v0.6, NanoPack v1.1.1.<br>Genome annotation: Tandem Repeats Finder v4.09, EDTA v2.0.1, RepeatMasker v4.1.1, STAR v2.7.10b, StringTie v3.0.0, pbmm2 v1.17.0, GMAP v2024-11-20, TransDecoder v5.7.1, cufflinks v2.2.1, miniprot v0.12, Augustus v3.5.0, GeneMark-ET, v4.72_lic, EvidenceModeler v2.1.0, PASA v2.5.3, DIAMOND v2.1.8, InterProScan v5.73.<br>Pangenome analysis: DAGchainer (no version, https://vcru.wisc.edu/simonlab/bioinformatics/programs/dagchainer/dagchainer_documentation.html), SynPan (no versions, https://github.com/dongyawu/PangenomeEvolution?tab=readme-ov-file), BLAST v2.16.0, MAFFT v7.526, IQ-TREE2 v2.3.5, Astral v5.7.8, MCMCtreeR v4.10.7, NLR-Annotator v2.0, GeneTribe v1.2.1, TASSEL v5.2.93, PLINK v1.9, PanPop vNC2024, vt v0.57721, AnchorWave v1.2.6, BWA v0.7.17, OrthoFinder v2.5.5, CAFE v5, bcftools v1.20.<br>Population analysis: BWA-MEM2 v2.3, LUSH_toolkit-HC v2.2.0, vg v1.56.0, Paragraph v2.3, SnpEff v5.2, Beagle v5.4, RAxML v8.2.12, ADMIXTURE v1.3.0, PopLDdecay v3.42, GCTA v1.94.1, Cytoscape v3.6, CytoCluster plugin v2.1.0, AutoAnnotate plugin v1.5.2, XPCLR v1.1.2, VCFtools v0.1.16, BEDTools v2.31.1, GenWin 1.0, GEMMA v0.98.3, LDAK v5.2, ABBABABAwindows.py (no versions, https://github.com/simonhmartin/genomics_general).<br>Code associated with this project is available at Github (https://github.com/Lucio-Yang/Pan-genome) and Figshare (https://doi.org/10.6084/m9.figshare.32213433).

For manuscripts utilizing custom algorithms or software that are central to the research but not yet described in published literature, software must be made available to editors and reviewers. We strongly encourage code deposition in a community repository (e.g. GitHub). See the Nature Portfolio guidelines for submitting code & software for further information.

Data

Policy information about availability of data

All manuscripts must include a data availability statement. This statement should provide the following information, where applicable:

- Accession codes, unique identifiers, or web links for publicly available datasets
- A description of any restrictions on data availability
- For clinical datasets or third party data, please ensure that the statement adheres to our policy

Genome assemblies and high-confidence gene annotations generated in this study have been archived in the Tetraploid Wheat Pan-Genome Database (http://omicsplant.cn/TetraploidWheatPangenomeDBs/) and Zenodo (https://doi.org/10.5281/zenodo.18466336).
All raw sequencing reads generated from HiFi, ONT, Hi-C, RNA-seq, Iso-seq, ChIP-seq and whole-genome resequencing, as well as assembled genome sequences, have been deposited at the National Genomics Data Center (NGDC) under accession PRJCA043839 (https://ngdc.cncb.ac.cn/bioproject/browse/PRJCA043839).

Research involving human participants, their data, or biological material

Policy information about studies with human participants or human data. See also policy information about sex, gender (identity/presentation), and sexual orientation and race, ethnicity and racism.

Reporting on sex and gender	N/A
Reporting on race, ethnicity, or other socially relevant groupings	N/A
Population characteristics	N/A
Recruitment	N/A
Ethics oversight	N/A

Note that full information on the approval of the study protocol must also be provided in the manuscript.

Field-specific reporting

Please select the one below that is the best fit for your research. If you are not sure, read the appropriate sections before making your selection.

☒ Life sciences ☐ Behavioural & social sciences ☐ Ecological, evolutionary & environmental sciences

For a reference copy of the document with all sections, see nature.com/documents/nr-reporting-summary-flat.pdf

Life sciences study design

All studies must disclose on these points even when the disclosure is negative.

Sample size	Twelve tetraploid wheat accessions were selected for de novo genome assembly. The 736 (including 121 public data) accessions were used for populations.
Data exclusions	No data were excluded.
Replication	All of the experiments were repeated at least 3 times. The number of independent experiments or biological replicates is indicated in the figure legends.
Randomization	For phenotype evaluation, we randomly selected 30 plants of each accession for field traits, and randomly selected 5 plants of each accession for abiotic-related traits.
Blinding	Blinding is not apply to genome sequencing.

Reporting for specific materials, systems and methods

We require information from authors about some types of materials, experimental systems and methods used in many studies. Here, indicate whether each material, system or method listed is relevant to your study. If you are not sure if a list item applies to your research, read the appropriate section before selecting a response.

Materials & experimental systems

n/a	Involved in the study	
☐	☒	Antibodies
☒	☐	Eukaryotic cell lines
☒	☐	Palaeontology and archaeology
☒	☐	Animals and other organisms
☒	☐	Clinical data
☒	☐	Dual use research of concern
☐	☒	Plants

Methods

n/a	Involved in the study	
☐	☒	ChIP-seq
☒	☐	Flow cytometry
☒	☐	MRI-based neuroimaging

Antibodies

Antibodies used

The peptide of the specific N-terminal tail of TaCENH3, 'ARTKHPAVRKTK', was expressed in the pET28b vector fused to a His-tag in Escherichia coli BL-21. An anti-rabbit polyclonal antibody against this peptide was prepared and purified using rabbit serum by AtaGenix (Wuhan, China).

Validation

The TaCENH3 antibody has been widely used for ChIP on wheat in previous publications (Walkowiak et al., 2020; Liu et al., 2023).

Dual use research of concern

Policy information about dual use research of concern

Hazards

Could the accidental, deliberate or reckless misuse of agents or technologies generated in the work, or the application of information presented in the manuscript, pose a threat to:

No	Yes	
☒	☐	Public health
☒	☐	National security
☒	☐	Crops and/or livestock
☒	☐	Ecosystems
☒	☐	Any other significant area

Experiments of concern

Does the work involve any of these experiments of concern:

No	Yes	
☒	☐	Demonstrate how to render a vaccine ineffective
☒	☐	Confer resistance to therapeutically useful antibiotics or antiviral agents
☒	☐	Enhance the virulence of a pathogen or render a nonpathogen virulent
☒	☐	Increase transmissibility of a pathogen
☒	☐	Alter the host range of a pathogen
☒	☐	Enable evasion of diagnostic/detection modalities
☒	☐	Enable the weaponization of a biological agent or toxin
☒	☐	Any other potentially harmful combination of experiments and agents

Plants

Seed stocks

All tetraploid wheat accessions were kept in Peking University Institute of Advanced Agricultural Sciences.

Novel plant genotypes

Not applicable.

Authentication

Not applicable.

ChIP-seq

Data deposition

☒ Confirm that both raw and final processed data have been deposited in a public database such as GEO.

☒ Confirm that you have deposited or provided access to graph files (e.g. BED files) for the called peaks.

Data access links
May remain private before publication.

ChIP-seq data have been deposited in the Genome Sequence Archive (GSA) database (https://ngdc.cncb.ac.cn/gsa/) under accession PRJCA043839.

Files in database submission

IG77365_CENH3_1_paired.fq.gz
IG77365_CENH3_2_paired.fq.gz
IG77365_Input_1_paired.fq.gz
IG77365_Input_2_paired.fq.gz
IG99236_CENH3_1_paired.fq.gz
IG99236_CENH3_2_paired.fq.gz
IG99236_Input_1_paired.fq.gz
IG99236_Input_2_paired.fq.gz
Kronos_CENH3_1_paired.fq.gz
Kronos_CENH3_2_paired.fq.gz
Kronos_Input_1_paired.fq.gz
Kronos_Input_2_paired.fq.gz
NU00021_CENH3_1_paired.fq.gz
NU00021_CENH3_2_paired.fq.gz
NU00021_Input_1_paired.fq.gz
NU00021_Input_2_paired.fq.gz
NU01905_CENH3_1_paired.fq.gz
NU01905_CENH3_2_paired.fq.gz
NU01905_Input_1_paired.fq.gz
NU01905_Input_2_paired.fq.gz
NU01954_CENH3_1_paired.fq.gz
NU01954_CENH3_2_paired.fq.gz
NU01954_Input_1_paired.fq.gz
NU01954_Input_2_paired.fq.gz
PI294478_CENH3_1_paired.fq.gz
PI294478_CENH3_2_paired.fq.gz
PI294478_Input_1_paired.fq.gz
PI294478_Input_2_paired.fq.gz
PI330553_CENH3_1_paired.fq.gz
PI330553_CENH3_2_paired.fq.gz
PI330553_Input_1_paired.fq.gz
PI330553_Input_2_paired.fq.gz
XM000719_CENH3_1_paired.fq.gz
XM000719_CENH3_2_paired.fq.gz
XM000719_Input_1_paired.fq.gz
XM000719_Input_2_paired.fq.gz
XM000834_CENH3_1_paired.fq.gz
XM000834_CENH3_2_paired.fq.gz
XM000834_Input_1_paired.fq.gz
XM000834_Input_2_paired.fq.gz
XM001097_CENH3_1_paired.fq.gz
XM001097_CENH3_2_paired.fq.gz
XM001097_Input_1_paired.fq.gz
XM001097_Input_2_paired.fq.gz
Zavitan_CENH3_1_paired.fq.gz
Zavitan_CENH3_2_paired.fq.gz

	Zavitan_Input_1_paired.fq.gz<br>Zavitan_Input_2_paired.fq.gz
Genome browser session<br>(e.g. UCSC)	N/A

Methodology

Replicates	N/A. All samples were obtained from 3-week-old seedlings.
Sequencing depth	All files were 150 bp pair-end sequencing.<br>IG77365_CENH3: 64325652 bp<br>IG77365_Input: 39334628 bp<br>IG99236_CENH3: 59123698 bp<br>IG99236_Input: 38679324 bp<br>Kronos_CENH3: 92965618 bp<br>Kronos_Input: 24052518 bp<br>NU00021_CENH3: 59731688 bp<br>NU00021_Input: 43652906 bp<br>NU01905_CENH3: 67529096 bp<br>NU01905_Input: 60880106 bp<br>NU01954_CENH3: 78116698 bp<br>NU01954_Input: 25576872 bp<br>PI294478_CENH3: 71835060 bp<br>PI294478_Input: 35818206 bp<br>PI330553_CENH3: 64361608 bp<br>PI330553_Input: 42423566 bp<br>XM000719_CENH3: 71307552 bp<br>XM000719_Input: 36633660 bp<br>XM000834_CENH3: 57130194 bp<br>XM000834_Input 46030068 bp<br>XM001097_CENH3: 72485872 bp<br>XM001097_Input: 35452426 bp<br>Zavitan_CENH3: 74034868 bp<br>Zavitan_Input: 23602426 bp
Antibodies	Wheat CenH3 antibody.
Peak calling parameters	MACS3 was used to call CENH3 ChIP-seq peaks using input as a control with the settings: -f BAMPE. Adjacent significant CENH3-enriched regions within 1,000 kb were merged, and the most significant regions were determined as potential centromere regions.
Data quality	Adapters and low-quality reads in ChIP-seq data for 12 accessions were removed using Trimmomatic v0.39 with the parameters "ILLUMINACLIP: adapter.fa:2:30:10 MINLEN:36 SLIDINGWINDOW: 4:20". Quality-controlled reads were aligned to each genome using Bowtie283 with the parameters (--very-sensitive --no-mixed --no-discordant -k 10 -X 1000) and were further filtered by SAMtools v1.10 with the parameters (-F 1804 -f 2 -q 30).
Software	Bowtie2 v2.5.1, SAMtools v1.10, MACS3 v3.0.1, Deeptools v3.5.5, StainedGlass v0.6, BEDtools v2.31.1
