# Supplementary material for: A pangenome of tetraploid wheat reveals the genetic architecture underlying domestication and genomic diversity for breeding
Source: Nat Genet. 2026 Jul 22;58(8):2027–39. doi: 10.1038/s41588-026-02678-9 (PMC13447107; doi:10.1038/s41588-026-02678-9)
Supplement: Supplementary file 5 [file 41588_2026_2678_MOESM3_ESM.pdf]

nature portfolio

Peer Review File

A pangenome of tetraploid wheat reveals the genetic architecture underlying domestication and genomic diversity for breeding

Corresponding Author: Professor Baoxing Song

This manuscript has been previously reviewed at another journal. This document only contains information relating to versions considered at Nature Genetics.

Version 2:

Decision Letter:

13th Mar 2026

Dear Professor Song,

Your Article, "A pangenome of tetraploid wheat reveals the genetic architecture underlying domestication and genomic diversity for breeding" has now been seen by 2 referees. You will see from their comments below that while they find your work of interest, some important points are raised. We are interested in the possibility of publishing your study in Nature Genetics, but would like to consider your response to these concerns in the form of a revised manuscript before we make a final decision on publication.

We therefore invite you to revise your manuscript taking into account all reviewer and editor comments. Please highlight all changes in the manuscript text file. At this stage we will need you to upload a copy of the manuscript in MS Word .docx or similar editable format.

We are committed to providing a fair and constructive peer-review process. Do not hesitate to contact us if there are specific requests from the reviewers that you believe are technically impossible or unlikely to yield a meaningful outcome.

When revising your manuscript:

*1) Include a “Response to referees” document detailing, point-by-point, how you addressed each referee comment. If no action was taken to address a point, you must provide a compelling argument. This response will be sent back to the referees along with the revised manuscript.

*2) If you have not done so already please begin to revise your manuscript so that it conforms to our Article format instructions, available
here.
Refer also to any guidelines provided in this letter.

*3) Include a revised version of any required Reporting Summary: https://www.nature.com/documents/nr-reporting-summary.pdf
It will be available to referees (and, potentially, statisticians) to aid in their evaluation if the manuscript goes back for peer review.
A revised checklist is essential for re-review of the paper.

Please be aware of our guidelines on digital image standards.

EXTENDED DATA FIGURES

When re-submitting your manuscript, please ensure that any supplementary figures and tables that are crucial to the manuscript's conclusions are converted into Extended Data figures and tables to increase visibility of these data. Extended Data figures and tables are online-only (present in the online PDF and full-text HTML versions of the paper), peer-reviewed display items that provide essential background to the article but are not included in the main article due to space constraints. A maximum of ten Extended Data display items (figures and tables) is permitted.

Please use the link below to submit your revised manuscript and related files:

Link Redacted

Note: This URL links to your confidential home page and associated information about manuscripts you may have submitted, or that you are reviewing for us. If you wish to forward this email to co-authors, please delete the link to your homepage.

We hope to receive your revised manuscript within four to eight weeks. If you cannot send it within this time, please let us know.

Please do not hesitate to contact me if you have any questions or would like to discuss these revisions further.

Nature Genetics is committed to improving transparency in authorship. As part of our efforts in this direction, we are now requesting that all authors identified as 'corresponding author' on published papers create and link their Open Researcher and Contributor Identifier (ORCID) with their account on the Manuscript Tracking System (MTS), prior to acceptance. ORCID helps the scientific community achieve unambiguous attribution of all scholarly contributions. You can create and link your ORCID from the home page of the MTS by clicking on 'Modify my Springer Nature account'. For more information please visit please visit www.springernature.com/orcid.

We look forward to seeing the revised manuscript and thank you for the opportunity to review your work.

Sincerely,

Wei Li, PhD
Senior Editor
Nature Genetics
www.nature.com/ng

Reviewers' Comments:

Reviewer #1 (Remarks to the Author):

Summary of the Key Results The manuscript presents a comprehensive graph-based pangenome for tetraploid wheat (Triticum turgidum L.) constructed from 12 newly de novo assembled high-quality genomes covering all 10 recognized subspecies. By integrating these with resequencing data from 736 accessions, the authors identify 448 loci associated with 32 agronomic traits, including yield and abiotic stress tolerance. A significant highlight is the functional characterization of the HD-ZIP transcription factor HAT14-B, which is shown to enhance spikelet number and grain size.
Originality and Significance This work is highly significant as it provides the most comprehensive description of tetraploid wheat genomic diversity to date. While individual reference genomes for wild emmer (Zavitan) and durum (Svevo) existed, this study's pangenome approach captures an average of 0.25 million structural variations (SVs) per accession and identifies genetic potential largely unexploited in modern bread wheat breeding.

Data & Methodology The methodology is robust and of high quality. The use of PacBio HiFi, ONT (for Kronos), and Hi-C reads resulted in highly contiguous assemblies (average contig-N50 of 43.2 Mb). The integration of a massive resequencing dataset (97 Tb) into a graph-based pangenome is a technically impressive feat that overcomes the limitations of single-reference genomes. Centromere identification via CENH3 ChIP-seq adds an extra layer of structural validation.
Appropriate Use of Statistics and Treatment of Uncertainties The statistical approaches are standard and well-applied, including BUSCO (99% completeness) and LAI (19.19) for assembly quality , and Ka/Ks analysis for selection pressure. GWAS and population structure analyses (using RAxML, ADMIXTURE, and PCA) are conducted with appropriate linkage-disequilibrium pruning and p-value thresholds.

Conclusions The conclusions regarding the genetic architecture of domestication and the role of specific genes like HAT14 are well-supported by the multi-omic data. The identification of distinct clades for wild, domesticated, and free-threshing wheat clarifies previously ambiguous evolutionary trajectories.

Suggested Improvements Overall, I am satisfied with the revision, no further experiments are requested. I do request to indicate seed availability, at least for the 12 reference genomes. If seed source is elsewhere, it should acknowledge. The primary critique involves the framing of the brittle-rachis evolution. While the authors identify it as "convergent," existing

haplotype data suggests a "monophyletic then convergent" model.
Updating this section with current literature (For example - Tracing the origin of non-brittle rachis alleles in wheat https://www.biorxiv.org/content/10.64898/2026.02.26.708176v1) will ensure the paper accurately reflects the state of the field.

Clarity and Context The manuscript is lucidly written.
The abstract clearly defines the untapped potential of tetraploid wheat for bread wheat improvement. The introduction provides necessary context on the 800,000-year evolutionary history of the species, and the conclusions effectively relate the findings to global food security.

Reviewer #2 (Remarks to the Author):

Summary: The paper presents a pangenome of tetraploid wheat, which contributes the A and B genomes of bread wheat. I have previously reviewed an earlier version of this paper. The authors have tightened the focus and provide a clear case for the usefulness of the genomic resources created to the wider community.

Originality: as previously, the work reported is in line with the format of other genome/pangenome papers. The scale of the resource provides novelty, as does the HAT14-B validation.

Data & methodology: many of the previous reviewer comments (from genomics experts) identified issues with the data and analysis approaches and I defer to their expertise on this important area of the work.

Appropriate use of statistics: noting that one of the GWAS groups includes only 81 individuals it is likely this is statistically under-powered. Previously requested power calculations are not included. Further information is requested on the specific methods from previous studies used (detailed below).

Conclusions: are broadly robust based on the findings presented. Specific suggestions, as detailed below.

References: broadly appropriate. Specific suggestion, as below.

Clarity and context: on the whole suitable (specific suggestions, as below).

Specific comments:
- Abstract L30: is harnessing tetraploid genomic diversity “essential” or does it provide a currently under explored opportunity? Suggest re-phrasing.
- Introduction L90: to avoid over-reaching suggest re-phrasing to “with potentially broad implications….”
- Methods L1094-1095: please provide more information about what specific approaches from the previous studies (ref 148, ref 149) were used for confirming candidate QTLs.
- Results L147: as a non-genome expert I struggle to understand what the “pangenome is closed” statement means.
- Results L239: can you please define what is meant by “dynamic changes” in the context of subgenome relationships? It is not clear (to a non-expert) what dynamic changes refers to, and how this links to the “bias” presented in L243.
- Results L321-322: check the accuracy of the statement “all tetraploids” to reference the BBAA forms in this study.
- Results L460: as a breeder I struggle with the opening sentence here: using the genomic resource to find useful traits. The useful traits should be the lead (as they exist already, you’re just trying to locate/characterise them). Suggest re-phrasing. Likewise take care with the use of the term “our” for the resources – they are no inherently owned by the study so suggest rephrase to “the generated genomic resources”. Also L512 suggest changing “our” to “the generated”. I’m not clear that the evidence shows “facilitating wheat improvement”, rather it provides new opportunities for wheat improvement.
- Discussion L565-567: this sentence would benefit from further clarity to improve understanding of what the authors mean. Also, are the authors sure that the “disease-resistance genes” they have analysed represent the full picture of quantitative resistance/s?
- Discussion L590-592: this statement should include a reference to the recent Schreiber et al (2024) review: https://www.nature.com/articles/s41576-024-00691-4
- Discussion L593: “innovation” is not the right term to use here, and this sentence should be improved. L595: remove repeat of “genetics”. L596: suggest removing “overcoming genetic homogeneity” as there is quite a detailed literature in this area and it is not expanded in this manuscript. L599: replace “as if relying on” with “as compared to” or similar.
- Discussion L606-609: suggest moving this sentence upwards in the paragraph (after L602) before talking about the usefulness of the resource.
- Discussion L612: consider removing “and other Triticeae crops” as though interesting you are introducing a new concept in the final words of the paper.

Alison Bentley

Version 3:

Decision Letter:

Our ref: NG-A71270R2

17th Apr 2026

Dear Dr. Song,

Thank you for submitting your revised manuscript "A pangenome of tetraploid wheat reveals the genetic architecture underlying domestication and genomic diversity for breeding" (NG-A71270R2). It has now been seen by the original referees and their comments are below. The reviewers find that the paper has improved in revision, and therefore we'll be happy in principle to publish it in Nature Genetics, pending minor revisions to satisfy the referees' final requests and to comply with our editorial and formatting guidelines.

We are now performing detailed checks on your paper and will send you a checklist detailing our editorial and formatting requirements soon. Please do not upload the final materials and make any revisions until you receive this additional information from us.

Thank you again for your interest in Nature Genetics Please do not hesitate to contact me if you have any questions.

Sincerely,
Wei

Wei Li, PhD
Senior Editor
Nature Genetics
www.nature.com/ng

Reviewer #2 (Remarks to the Author):

Thanks for diligently addressing all of the points raised in my review of the previous version. In particular, I appreciate the willingness to conduct power analyses and to remove QTL where power calculations indicate population sizes are statistically underpowered.

I note that Reviewer 1 has requested clarity on seed availability for the 12 accessions, and the authors have indicated these are "available on request". I would ask the authors to consider if depositing the accessions in an international gene bank would facilitate greater downstream impact from the work. It is difficult to justify the value of publishing excellent genomic resources such as this without the foundation seed being available.

Alison Bentley

Open Access This Peer Review File is licensed under a Creative Commons Attribution 4.0 International License, which permits use, sharing, adaptation, distribution and reproduction in any medium or format, as long as you give appropriate credit to the original author(s) and the source, provide a link to the Creative Commons license, and indicate if changes were made.

In cases where reviewers are anonymous, credit should be given to 'Anonymous Referee' and the source.
The images or other third party material in this Peer Review File are included in the article's Creative Commons license, unless indicated otherwise in a credit line to the material. If material is not included in the article's Creative Commons license and your intended use is not permitted by statutory regulation or exceeds the permitted use, you will need to obtain permission directly from the copyright holder.
To view a copy of this license, visit https://creativecommons.org/licenses/by/4.0/

Authors' responses to Reviewers' Comments

We sincerely thank the Editor and Reviewers for their insightful feedback, which has greatly contributed to the improvement of our manuscript. Below is our detailed response.

Reviewers' Comments:

Reviewer #1 (Remarks to the Author):

Summary of the Key Results: The manuscript presents a comprehensive graph-based pangenome for tetraploid wheat (*Triticum turgidum* L.) constructed from 12 newly de novo assembled high-quality genomes covering all 10 recognized subspecies. By integrating these with resequencing data from 736 accessions, the authors identify 448 loci associated with 32 agronomic traits, including yield and abiotic stress tolerance. A significant highlight is the functional characterization of the HD-ZIP transcription factor HAT14-B, which is shown to enhance spikelet number and grain size.

Response: Thank you for your comprehensive summary of our manuscript. We greatly appreciate your recognition of the key findings and the careful consideration of our work.

Originality and Significance: This work is highly significant as it provides the most comprehensive description of tetraploid wheat genomic diversity to date. While individual reference genomes for wild emmer (Zavitan) and durum (Svevo) existed, this study's pangenome approach captures an average of 0.25 million structural variations (SVs) per accession and identifies genetic potential largely unexploited in modern bread wheat breeding.

Response: Thank you for your positive evaluation of the originality and significance of our work. We are pleased that our pangenome approach and the identification of structural variations provide valuable genetic resources for wheat breeding.

Data & Methodology: The methodology is robust and of high quality. The use of PacBio HiFi, ONT (for Kronos), and Hi-C reads resulted in highly contiguous assemblies (average contig-N50

of 43.2 Mb). The integration of a massive resequencing dataset (97 Tb) into a graph-based pangenome is a technically impressive feat that overcomes the limitations of single-reference genomes. Centromere identification via CENH3 ChIP-seq adds an extra layer of structural validation.

Response: Thank you for your positive feedback on our data and methodology. We are glad that you appreciate the high-quality assemblies and our approach of integrating large-scale resequencing data into the pangenome, as well as the centromere identification.

Appropriate Use of Statistics and Treatment of Uncertainties: The statistical approaches are standard and well-applied, including BUSCO (99% completeness) and LAI (19.19) for assembly quality, and Ka/Ks analysis for selection pressure. GWAS and population structure analyses (using RAxML, ADMIXTURE, and PCA) are conducted with appropriate linkage-disequilibrium pruning and p-value thresholds.

Response: Thank you for your positive feedback. We are glad that you appreciate our standard approaches for assembly quality assessment and GWAS analysis.

Conclusions: The conclusions regarding the genetic architecture of domestication and the role of specific genes like HAT14 are well-supported by the multi-omic data. The identification of distinct clades for wild, domesticated, and free-threshing wheat **clarifies previously ambiguous** evolutionary trajectories.

Response: Thank you for your positive feedback on our observations.

Suggested Improvements: Overall, I am satisfied with the revision, no further experiments are requested. I do request to indicate seed availability, at least for the 12 reference genomes. If seed source is elsewhere, it should acknowledge. **The primary critique involves the framing of the brittle-rachis evolution.** While the authors identify it as "convergent," existing haplotype data suggests a "monophyletic then convergent" model.

Updating this section with current literature (For example - Tracing the origin of non-brittle rachis alleles in wheat https://www.biorxiv.org/content/10.64898/2026.02.26.708176v1) will ensure the paper accurately reflects the state of the field.

Response: Thank you for your constructive feedback and suggestions for improvement. We appreciate your satisfaction with the revision. And we indicated seed availability for the 12 reference genomes, by “Seed of 12 tetraploid wheat accessions assembled in this study are available from the corresponding author upon request for research purposes.”. We have indicated in Supplemental Table 12 the sources for all seed accessions.

We also understand your critique regarding the framing of brittle-rachis evolution. We have updated this section to reflect an independent origins model based on existing haplotype data and incorporated the bioRxiv literature which reports two loss-of-function *Btr1-A* alleles emerged in wild emmer subpopulations. We modified any text that might lead to misunderstanding about this point.

Taking the discussion section for example, “Persian wheat is also markedly divergent from other free-threshing tetraploids and harbours a loss-of-function *Btr1-A* allele without the canonical 2-bp deletion, while having a 5,034-bp LTR/Copia insertion in the protein-coding region. A recent study indicates that independent loss-of-function alleles likely emerged in wild emmer populations (Cavalet-Giorsa et. al. 2026), highlighting the complexity of crop evolution.”.

Your insights help ensure that our paper accurately represents the current state of the field. Thank you again for your valuable input.

Clarity and Context: The manuscript is lucidly written.
The abstract clearly defines the untapped potential of tetraploid wheat for bread wheat improvement. The introduction provides necessary context on the 800,000-year evolutionary history of the species, and the conclusions effectively relate the findings to global food security.
Response: Thank you for your positive feedback. We are glad that you find the manuscript clearly written and that the abstract effectively highlights the untapped potential of tetraploid wheat for bread wheat improvement. We also appreciate your recognition of the introduction for providing necessary evolutionary context and of the conclusions for connecting our findings to global food security.

Reviewer #2 (Remarks to the Author):

Summary: The paper presents a pangenome of tetraploid wheat, which contributes the A and B genomes of bread wheat. I have previously reviewed an earlier version of this paper. The authors have tightened the focus and provide a clear case for the usefulness of the genomic resources created to the wider community.

Response: Thank you for your thoughtful feedback. We appreciate your recognition of the improvements made in the revised manuscript, particularly the clearer focus and the demonstrated usefulness of the genomic resources to the wider community. Your continued support and insights are invaluable to us.

Originality: as previously, the work reported is in line with the format of other genome/pangenome papers. The scale of the resource provides novelty, as does the HAT14-B validation.

Response: Thank you for your feedback. We appreciate your recognition of the novelty brought by the resource scale and the HAT14-B validation.

Data & methodology: many of the previous reviewer comments (from genomics experts) identified issues with the data and analysis approaches and I defer to their expertise on this important area of the work.

Response: Thank you for your feedback. We updated our analysis in response to the valuable comments from other reviewers. As we will see, he/she is satisfied with the revised approaches.

Appropriate use of statistics: noting that one of the GWAS groups includes only 81 individuals it is likely this is statistically under-powered. Previously requested power calculations are not included. Further information is requested on the specific methods from previous studies used (detailed below).

Response: We greatly appreciate your careful and constructive feedback on the statistical rigor of our GWAS analyses. We fully agree that the GWAS group with 81 individuals may indeed be underpowered. To address this concern, we conducted power calculations using different sample sizes.

Based on the phenotypic data, we calculated the narrow-sense heritability for each trait category: 0.75 on average for yield-related and plant architecture traits, and 0.25 on average for abiotic stress tolerance traits, as detailed and visualized in Supplementary Fig. 28. Further calculations revealed that the effect size of the leading QTL for yield-related and plant architecture traits was ($r^2 = 0.13$), while the effect size for abiotic stress traits was ($r^2 = 0.05$). The full results of this statistical power analysis, including power thresholds for varying QTL effect sizes and sample sizes, are summarized in Table 1 and illustrated in Fig. 1. The code used for these calculations is available at https://github.com/baoxingsong/GWASStatisticalPower.

Our power analysis confirmed that the GWAS results derived from 81 samples are indeed statistically underpowered. As the sample size increases, the statistical power improves significantly. In light of the low power indicated, we have removed any GWAS results with a population size smaller than 241 from the updated manuscript.

Once again, we thank you for your valuable feedback and insights.

Table 1. Statistical power analysis for different sample sizes and QTL effect size.

**Sample Size**	**r^2=0.01**	**r^2=0.03**	**r^2=0.05**	**r^2=0.07**	**r^2=0.09**	**r^2=0.11**	**r^2=0.12**	**r^2=0.13**	**r^2=0.14**
81	0.00001	0.00011	0.00068	0.00256	0.00739	0.01768	0.02590	0.03679	0.05082
132	0.00002	0.00058	0.00424	0.01717	0.04896	0.10952	0.15255	0.20412	0.26348
160	0.00003	0.00117	0.00898	0.03610	0.09848	0.20575	0.27519	0.35249	0.43462
241	0.00010	0.00557	0.04394	0.15646	0.34972	0.57515	0.67885	0.76815	0.84023
260	0.00013	0.00750	0.05833	0.19922	0.42181	0.65563	0.75370	0.83268	0.89207
373	0.00044	0.03062	0.20117	0.51213	0.78974	0.93594	0.96882	0.98601	0.99420

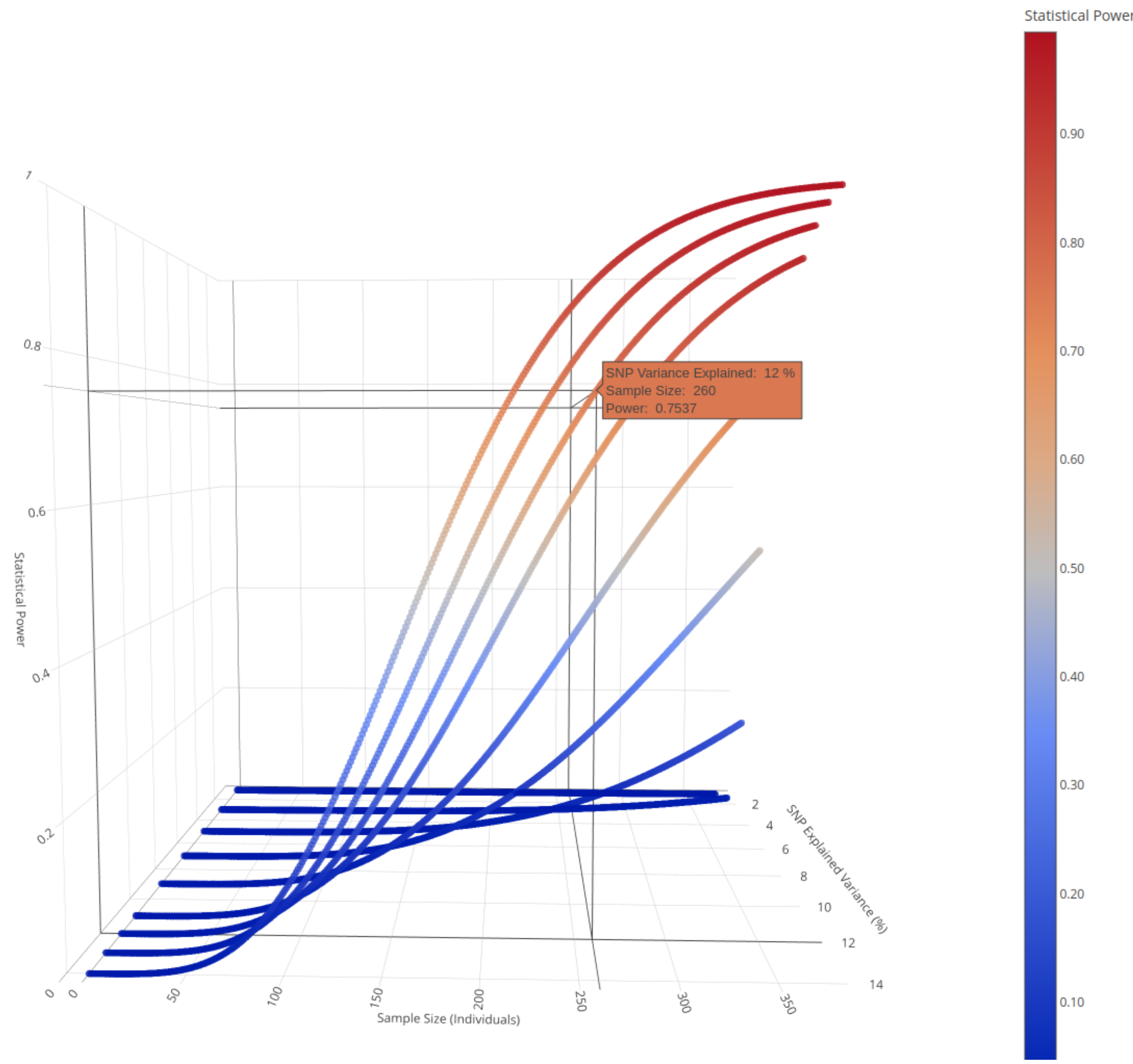

Fig. 1 Statistical power analysis for different sample sizes and effect size of QTL.

Conclusions: are broadly robust based on the findings presented. Specific suggestions, as detailed below.

Response: Thank you for your positive assessment of our conclusions. We have addressed the specific comments you mentioned below.

References: broadly appropriate. Specific suggestion, as below.

Response: Thank you for your positive assessment. We have made the specific adjustments you suggested, as detailed below.

Clarity and context: on the whole suitable (specific suggestions, as below).

Response: Thank you for your feedback. We have made the specific adjustments to improve clarity and context as suggested below.

Specific comments:

- **Abstract L30:** is harnessing tetraploid genomic diversity "essential" or does it provide a currently under explored opportunity? Suggest re-phrasing.

Response: Thank you for your constructive suggestion. We agree and have changed it to: "Harnessing its extensive genomic diversity presents a currently underutilized opportunity for enhancing agronomic traits, sustainability, and climatic adaptability in bread wheat." We believe the revised phrasing better reflects the current state of research in this area while emphasizing the importance of utilizing tetraploid wheat genomic diversity. Thank you again for your valuable suggestion.

- **Introduction L90:** to avoid over-reaching suggest re-phrasing to "with potentially broad implications…."

Response: Thank you for your suggestion. We agree to revise the phrasing to "with potentially broad implications."

- **Methods L1094-1095:** please provide more information about what specific approaches from the previous studies (ref 148, ref 149) were used for confirming candidate QTLs.

Response: We sincerely thank the reviewer for the valuable comment. We have revised the sentence in question and provided a more detailed description of the methods used to confirm candidate QTLs. Specifically, "following approaches described in previous studies (ref. 150, ref. 151), candidate QTLs and genes were identified through a multi-step procedure. First, within a 1-Mb region corresponding to the linkage disequilibrium decay distance, the signal with the lowest P-value was designated as the leading SNP, small indel, or structural variant (SV). Next, regions extending 1 Mb upstream and downstream of the leading variant, containing at least two statistically significant loci, were defined as candidate QTLs. Finally, genes located within the linkage disequilibrium regions corresponding to these QTLs were considered as candidate genes."

- **Results L147:** as a non-genome expert I struggle to understand what the "pangenome is closed" statement means.

Response: Thank you for your feedback. To clarify, "pangenome is closed" means that when additional genomes are added to the analysis, the number of novel genes discovered plateaus—indicating diminishing returns in gene discovery. This suggests that the current set of accessions adequately represents the genomic diversity of tetraploid wheat. To clarify this point, we rephrased it as "the tetraploid wheat pangenome approached a plateau", which is more frequently used.

- **Results L239:** can you please define what is meant by "dynamic changes" in the context of subgenome relationships? It is not clear (to a non-expert) what dynamic changes refers to, and how this links to the "bias" presented in **L243**.

Response: Thanks for raising this point. By "Dynamic changes" and " Bias", we mean asymmetric evolution between subgenomes, include things like rearrangements of chromosomes, chromosome length and unequal gene abundance.

To avoid the confusing, we rephased it as "Pan-genome data enable characterization of asymmetric evolution between subgenomes."

- **Results L321-322:** check the accuracy of the statement "all tetraploids" to reference the BBAA forms in this study.

Response: Thank you for your careful comment. Upon checking, we confirm that all 736 accessions in this study belong to BBAA tetraploid wheat (*Triticum turgidum* L., BBAA, 2n=4x=28). To avoid any potential misunderstanding, we have revised the text and changed "all tetraploids" to "these 736 accessions" to accurately reflect the scope of our study.

- **Results L460:** as a breeder I struggle with the opening sentence here: using the genomic resource to find useful traits. The useful traits should be the lead (as they exist already, you're just trying to locate/characterize them). Suggest re-phrasing. Likewise take care with the use of the term "our" for the resources – they are no inherently owned by the study so suggest rephrase to "the generated genomic resources". Also, **L512** suggest changing "our" to "the generated". I'm not clear that the

evidence shows “facilitating wheat improvement”, rather it provides new opportunities for wheat improvement.

Response: Thank you for your valuable suggestions. We have addressed all three points as follows:

L460: We agree that the useful traits should be the lead, as they already exist in the germplasm and we are simply trying to locate and characterize them. We have also changed "our" to "the generated" for the genomic resources. The revised sentence now reads:

"We evaluated 373 re-sequenced tetraploid wheat accessions for 32 diverse traits that encompass contemporary breeding goals—including seven plant-architecture traits, eight grain-related traits, and 17 abiotic-stress related traits—to identify variants associated with them using the generated genomic resources."

L512: We have modified "our" to "the generated" and changed **"facilitating wheat improvement"** to **"providing new opportunities for wheat improvement,"** as we agree that the evidence presented supports this more cautious interpretation.

We appreciate your careful review and helpful comments.

- Discussion L565-567: this sentence would benefit from further clarity to improve understanding of what the authors mean. Also, are the authors sure that the “disease-resistance genes” they have analyzed represent the full picture of quantitative resistance/s?

Response: Thank you very much for your suggestion. We sincerely apologize for the unclear expression in the original text and would like to further clarify:

The evolutionary analysis of gene families indicates that, compared to domesticated tetraploid wheat, genes were contracted in free-threshing tetraploid wheat. Enrichment analysis reveals that these contracted genes are significantly enriched in disease resistance-related GO terms.

Additionally, these genes represent only a portion of the genes involved in the contraction process. To avoid any misguidance, we modified the sentence as “Disease-resistance genes are enriched in contracted gene families from the domesticated to the free-threshing population.”.

Thank you again for your valuable feedback.

- Discussion L590-592: this statement should include a reference to the recent Schreiber et al (2024) review: https://www.nature.com/articles/s41576-024-00691-4

Response: Thank you for your valuable suggestion. We agree that incorporating the recent review by Schreiber et al. (2024) enhances this statement. We have revised the sentence as follows:

"These findings underscore the potential of pan-genomic approaches to reveal novel domestication variants and provide new targets for accelerated crop improvement and de novo domestication (Schreiber et al., 2024)."

We have ensured that the reference is properly cited in the manuscript. Thank you once again for your guidance, and we look forward to your further recommendations.

- Discussion L593: "innovation" is not the right term to use here, and this sentence should be improved. **L595:** remove repeat of "genetics". **L596:** suggest removing "overcoming genetic homogeneity" as there is quite a detailed literature in this area and it is not expanded in this manuscript. **L599:** replace "as if relying on" with "as compared to" or similar.

Response: Thank you very much for your constructive suggestions. We have revised the manuscript according to your comments:

L593: We have replaced "The innovation of wheat varieties" with " The development of new wheat varieties"

L595: We have removed the repeated word "genetic."

L596: We have removed "for overcoming genetic homogeneity" as suggested.

L599: We have replaced "as if relying on" with "as compared to."

Thank you again for your valuable feedback.

- Discussion L606-609: suggest moving this sentence upwards in the paragraph (after L602) before talking about the usefulness of the resource.

Response: Thank you very much for your suggestion. We have moved the sentence (L606-609) to after L602, which improves the logical flow of the discussion and better introduces the usefulness of the resource. Thank you again for your valuable suggestion.

- **Discussion L612:** consider removing “and other Triticeae crops” as though interesting you are introducing a new concept in the final words of the paper.

Response: Thank you very much for your suggestion. We have removed 'and other Triticeae crops' from Discussion in the revised version.

Alison Bentley

Authors' responses to Reviewers' Comments

Dear Dr. Wei Li,

Thank you for handling our manuscript titled "**A Pangenome of Tetraploid Wheat Reveals the Genetic Architecture Underlying Domestication and Genomic Diversity for Breeding" (NG-A71270R2)**. We appreciate your principled publication of this manuscript, and we are deeply grateful for all the valuable suggestions from the Editors and the Reviewers. We have revised our manuscript following all the points as **Reviewer 2** and checklist suggested.

Reviewers' Comments:

Reviewer #1:

None

Response:

We sincerely thank Reviewer #1 for the careful review and valuable guidance during the peer review process. We are deeply encouraged and honored to receive your recognition of our manuscript, which motivates us to further refine our research work with greater rigor and dedication. We will continue to uphold the high standards of academic research and make every effort to improve the manuscript to meet the journal's requirements.

Reviewer #2:

Remarks to the Author:

Thanks for diligently addressing all of the points raised in my review of the previous version. In particular, I appreciate the willingness to conduct power analyses and to remove QTL where power calculations indicate population sizes are statistically underpowered.

I note that Reviewer 1 has requested clarity on seed availability for the 12 accessions, and the authors have indicated these are "available on request". I would ask the authors to consider if depositing the accessions in an international gene bank would facilitate greater downstream impact from the work. It is difficult to justify the value of publishing

excellent genomic resources such as this without the foundation seed being available.

Alison Bentley

Response:

We greatly appreciate your positive comments and valuable suggestions. We highly thank you for recognizing our efforts in conducting power analyses and removing QTL loci with statistically underpowered population sizes.

In response to your thoughtful suggestion, we revised our data availability statement as "Seed of 12 tetraploid wheat accessions assembled in this study are available from the corresponding author upon request for research purposes and their germplasm identifiers in gene bank are provided in Supplementary Table 1.". We have expanded Supplementary Table 1 to include the direct gene bank URLs for each accession, facilitating access.

Once again, we would like to express our sincere gratitude for your precious time, meticulous evaluation and valuable constructive remarks. Your profound insights have contributed substantially to polishing and perfecting our manuscript.

We believe the key concerns have been well resolved and hope that the revised manuscript is accepted for publication.

Sincerely,

Baoxing Song, Xiaojun Nie, Hude Mao, Xing Wang Deng, and colleagues
baoxing.song@pku-iaas.edu.cn
